# Heteroaromatic Pyrazole-Based Carbohydrazones: Structure-Dependent Redox Activity, DNA-Associated Spectroscopic Behavior, and Multifunctional Biological Properties

**DOI:** 10.3390/molecules31122031

**Published:** 2026-06-10

**Authors:** Aliye Gediz Erturk, Ertuğrul Yiğit

**Affiliations:** Department of Chemistry, Faculty of Science & Arts, Ordu University, 52200 Ordu, Turkey; ertugrulygt.55@gmail.com

**Keywords:** pyrazole-based carbohydrazones, heteroaromatic substitution, antioxidant activity, photoprotective activity, CT-DNA-associated spectroscopic response, cytotoxicity, A431 epidermoid carcinoma cells, structure–activity relationship

## Abstract

Six novel pyrazole-based carbohydrazone derivatives (**3a**–**3f**) bearing structurally diverse heteroaromatic substituents were synthesized and characterized by ATR-FTIR, ^1^H NMR, APT-^13^C NMR, and HRMS analyses. Their multifunctional bioactivity was evaluated using antioxidant, photoprotective, CT-DNA-associated spectroscopic response, cytotoxicity, and scratch wound closure assays. Antioxidant activity was assessed by DPPH radical scavenging, Fe^2+^ chelation, and ferric thiocyanate (FTC) assays against appropriate reference standards, while photoprotective potential was determined by spectrophotometric SPF analysis using carrot seed oil as a reference. The benzothiazole-containing derivative (**3f**) showed the strongest DPPH scavenging activity, FTC antioxidant capacity, and photoprotective activity, while also producing one of the most pronounced CT-DNA-associated spectroscopic responses under the experimental conditions employed. In contrast, the benzimidazole derivative (**3e**) displayed the highest Fe^2+^ chelating activity among the synthesized compounds. In cell-based assays, the imidazole- and thiazole-containing derivatives (**3b** and **3c**) showed the most favorable balance between growth-inhibitory potency and selectivity toward A431 epidermoid carcinoma cells relative to HaCaT keratinocytes. Scratch assay results did not support direct anti-migratory activity under the tested conditions but indicated compound-dependent modulation of wound-closure-associated cellular responses. Overall, these findings demonstrate that heteroaromatic substitution strongly modulates redox behavior, CT-DNA-associated spectroscopic behavior, photophysical properties, and cytotoxic selectivity in pyrazole-based carbohydrazones, identifying this scaffold as a structurally tunable platform for further bioactivity optimization.

## 1. Introduction

Cancer remains a major global health challenge and continues to drive the search for structurally diverse small molecules with improved biological selectivity and multifunctional activity profiles [1,2]. In medicinal chemistry, heterocyclic compounds occupy a central role because heteroatom-containing ring systems frequently exhibit favorable pharmacokinetic properties together with broad biological activity [3]. Among these scaffolds, pyrazole derivatives have attracted considerable attention due to their reported anti-inflammatory, antimicrobial, antioxidant, and growth-inhibitory activities [4,5,6]. The pyrazole nucleus is particularly valuable because its aromatic nitrogen-containing framework can participate in hydrogen bonding, dipole interactions, and π-stacking with biological targets, thereby contributing to diverse pharmacological effects [4,7]. In addition, pyrazole-based compounds have been associated with modulation of oxidative stress pathways, apoptosis-related signaling, and DNA-interactive behavior in cancer-associated cellular systems [5,6].

Hydrazone and carbohydrazone derivatives also represent an important class of bioactive molecules with documented antioxidant, antimicrobial, antitubercular, and cytotoxic properties [8,9,10]. The azomethine (–C=N–) linkage present in hydrazone systems contributes to conformational flexibility and facilitates intermolecular interactions through hydrogen bonding and electron delocalization [8,11]. Furthermore, carbohydrazone-containing compounds may exhibit metal-chelating and redox-associated behavior, both of which are relevant to oxidative-stress-related cellular processes [12]. Because oxidative stress is closely associated with carcinogenesis, inflammation, DNA damage, and ultraviolet-induced skin injury, compounds capable of modulating redox processes remain important targets in the development of multifunctional biologically active molecules [13,14].

Hybrid pharmacophore design has emerged as an effective strategy for generating compounds with enhanced or diversified biological activity [15]. In particular, incorporation of heteroaromatic substituents into pyrazole–carbohydrazone systems may substantially influence electron distribution, lipophilicity, molecular planarity, and intermolecular interaction capacity [16]. Heteroaromatic systems such as pyrrole, imidazole, thiazole, benzimidazole, and benzothiazole are especially relevant because nitrogen- and sulfur-containing aromatic rings are frequently involved in biological recognition processes and may contribute to stabilization of ligand–biomolecule interactions through donor–acceptor mechanisms and π-conjugation effects [16,17]. Such structural modifications may therefore influence oxidative behavior, photophysical properties, and biological activity profiles in relevant cellular systems [18].

DNA-interactive small molecules remain important in medicinal chemistry because DNA binding may influence transcriptional processes, replication, and apoptosis-associated cellular responses [18]. Spectroscopic studies involving DNA are therefore frequently used as preliminary tools to examine ligand-induced spectral changes in the presence of nucleic acids and to explore potential biomolecular interactions [19]. In addition, antioxidant and photoprotective properties are increasingly investigated together with cytotoxic behavior in multifunctional heterocyclic systems, particularly in skin-associated cellular models that are continuously exposed to oxidative and ultraviolet-induced stress [14,20].

Human epidermoid carcinoma A431 cells are commonly used as a malignant keratinocyte model for growth-inhibitory studies, whereas HaCaT keratinocytes serve as a non-tumorigenic skin cell model for comparative selectivity evaluation [21,22]. Comparative assessment of compound activity in malignant and non-malignant keratinocyte systems may therefore provide insight into preferential cytotoxic behavior toward cancer-associated epidermal cells.

Based on these considerations, six novel pyrazole-based carbohydrazone derivatives (**3a**–**3f**) containing different heteroaromatic substituents were synthesized and structurally characterized using ATR-FTIR, ^1^H NMR, APT-^13^C NMR, and HRMS analyses. The synthesized compounds were subsequently evaluated for their antioxidant properties using DPPH radical scavenging, ferrous ion chelation, and ferric thiocyanate (FTC) assays together with spectrophotometric sun protection factor (SPF) determination. Spectroscopic responses observed in the presence of calf thymus DNA (CT-DNA) were evaluated using UV-Vis absorption and fluorescence spectroscopy. Furthermore, the biological effects of the compounds were examined in A431 and HaCaT cells using MTT viability and scratch assays in order to evaluate cytotoxic behavior and wound-closure-associated cellular responses. Particular emphasis was placed on examining how heteroaromatic substitution influenced redox activity, CT-DNA-associated spectroscopic behavior, and preferential activity toward malignant keratinocytes.

## 2. Results and Discussion

### 2.1. Chemistry

**Scheme 1:**
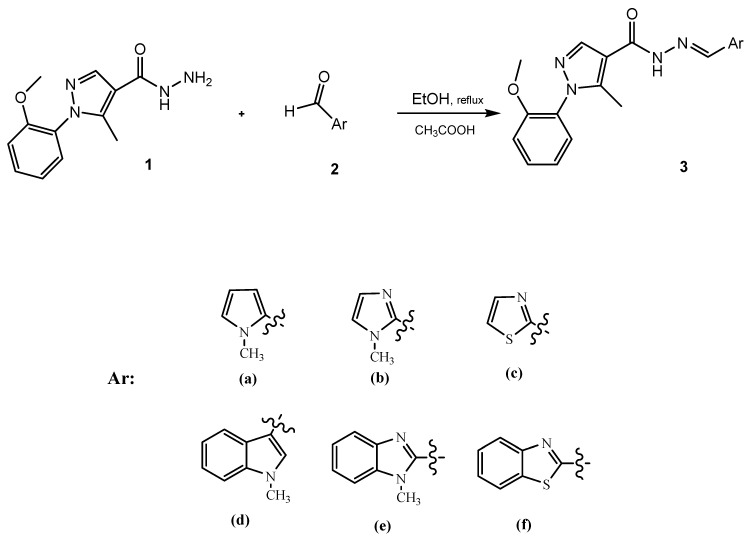
Synthetic route for the preparation of compounds **3a**–**3f** from pyrazole carbohydrazide **1** and heteroaromatic aldehydes **2a**–**2f**.

As shown in Scheme 1, the target hydrazone derivatives (**3a**–**3f**) were synthesized via condensation reactions between 1-(2-methoxyphenyl)-5-methyl-1H-pyrazole-4-carbohydrazide (**1**) and the corresponding heteroaromatic aldehydes (**2a**–**2f**), as illustrated in Scheme 1. The reaction proceeded smoothly in absolute ethanol under reflux conditions in the presence of a catalytic amount of glacial acetic acid. The formation of the hydrazone linkage (-C=N-NH-) follows a typical nucleophilic addition–elimination pathway, in which the terminal hydrazide -NH_2_ group attacks the carbonyl carbon of the aldehyde, followed by dehydration to afford the corresponding hydrazone product. All compounds were obtained in moderate to good yields (40–80%) as stable solid products. The reaction progress was monitored by thin-layer chromatography (TLC), and completion was confirmed by the disappearance of both aldehyde and hydrazide spots. Purification was achieved by successive washing (diethyl ether and cold ethanol) followed by recrystallization from acetonitrile. Chromatographic purification was intentionally avoided due to the known susceptibility of hydrazone linkages to partial hydrolysis or decomposition on silica surfaces, which can compromise product integrity and yield. The applied purification strategy provided analytically pure compounds, as confirmed by spectroscopic analyses.

The formation of the hydrazone (C=N) linkage may theoretically give rise to E/Z isomerism. However, the ^1^H NMR spectra of all compounds **3a**–**3f** displayed a single set of signals for the azomethine (CH=N) proton, indicating the presence of a single predominant isomer in solution. This observation is consistent with the preferential formation of the thermodynamically more stable E-isomer, commonly favored due to reduced steric repulsion and extended conjugation, as frequently reported for hydrazone and carbohydrazone derivatives [23,24].

The structures of compounds **3a**–**3f** were confirmed by FTIR (ATR), ^1^H NMR, APT-^13^C NMR, and HRMS analyses. The formation of the hydrazone linkage was supported by characteristic C=O and C=N stretching bands in the FTIR spectra, together with the appearance of a distinct azomethine proton signal in the ^1^H NMR spectra. The APT-^13^C NMR spectra further confirmed the presence of carbonyl and aromatic carbon environments, while HRMS data verified the molecular compositions of all compounds with high accuracy. Detailed spectroscopic analyses are presented in Section 2.2.

The variation in the heteroaromatic aldehyde moiety (pyrrole, imidazole, thiazole, indole, benzimidazole, and benzothiazole) did not significantly affect the course of the condensation reaction, indicating the robustness of the synthetic protocol. Moderate variations in reaction time were observed among the different heteroaromatic aldehydes. The indole derivative reacted most rapidly (3 h), whereas the pyrrole and imidazole derivatives required longer reaction times (5 h), and the remaining substrates afforded the products within 4 h. Overall, the results demonstrate that the developed synthetic approach is broadly applicable to structurally diverse heteroaromatic aldehydes.

### 2.2. Spectroscopy

#### 2.2.1. FTIR Spectroscopy

The FTIR (ATR) spectra of compounds **3a**–**3f** provided clear evidence for the successful formation of the hydrazone framework and the preservation of key functional groups (see Figures S1–S6 in the Supporting Information). All compounds exhibited broad absorption bands in the region of ~3130–3270 cm^−1^, which can be attributed to the N-H stretching vibration of the hydrazone moiety. The observed band broadening is consistent with the presence of intramolecular hydrogen bonding, which is known to influence N-H stretching frequencies in such systems [25]. Aromatic and heteroaromatic C-H stretching vibrations were observed in the range of 3100–3000 cm^−1^, while aliphatic C-H stretching bands corresponding to methyl and methoxy groups appeared in the region of ~2970–2839 cm^−1^, consistent with literature data for substituted aromatic compounds [26]. The presence of a conjugated amide carbonyl group was confirmed by strong absorption bands in the range of 1627–1674 cm^−1^. The variation in the position of the C=O stretching band among the compounds reflects differences in conjugation and electronic effects introduced by the heteroaromatic substituents, as commonly observed in hydrazone derivatives [27]. The formation of the azomethine linkage was further supported by absorption bands observed around ~1590–1610 cm^−1^, associated with azomethine (C=N) stretching. In some compounds (e.g., **3c**), these bands appear merged within the aromatic skeletal vibration region (~1576–1481 cm^−1^), which is typical for conjugated heteroaromatic systems where vibrational modes are strongly coupled [26,28].

Additional bands in the region of ~1550–1400 cm^−1^ were attributed to aromatic and heteroaromatic skeletal vibrations, consistent with characteristic ring stretching modes reported for substituted aromatic systems [26,29]. The C-O stretching vibrations of the methoxy substituent were consistently observed between ~1265–1010 cm^−1^, in agreement with the literature data for aryl ether functionalities [29]. In the lower frequency region (~870–650 cm^−1^), bands corresponding to out-of-plane bending vibrations of substituted aromatic and heteroaromatic C-H groups were observed, in accordance with established infrared spectral correlations [26,30]. In sulfur-containing derivatives (**3c** and **3f**), contributions in this region may also be associated with vibrational modes of heteroaromatic rings containing C-S bonds, although these assignments remain tentative due to overlapping vibrational features [28]. The observed variation in band positions across the series may reflect the influence of the heteroaromatic substituents on the degree of conjugation within the hydrazone framework. Overall, the FTIR data are in good agreement with the proposed structures and confirm the successful formation of the targeted hydrazone derivatives.

#### 2.2.2. ^1^H NMR Spectroscopy

The ^1^H NMR spectra of compounds **3a**–**3f** were consistent with the proposed hydrazone structures (see Figures S7–S12 in the Supporting Information). A characteristic singlet corresponding to the azomethine (CH=N) proton was observed in the range of δ 8.02–8.71 ppm, while the hydrazide NH proton resonated significantly downfield at δ 11.19–14.47 ppm, in agreement with reported data for conjugated hydrazone systems [23,31]. The pronounced variation in NH chemical shift across the series reflects differences in the local electronic environment and hydrogen-bonding interactions. In particular, **3b** exhibited an unusually downfield NH signal (δ 14.47 ppm), suggesting a highly deshielded environment. Considering that the measurements were performed in DMSO-d_6_, this behavior is best interpreted as the combined effect of conjugation and hydrogen-bonding interactions rather than a single well-defined intramolecular hydrogen bond [32].

The azomethine proton also showed substituent-dependent shifts. The most downfield signals were observed for **3c** (δ 8.62), **3d** (δ 8.53), **3e** (δ 8.54), and **3f** (δ 8.71), whereas **3b** (δ 8.02) appeared significantly upfield. These differences indicate that the heteroaromatic substituents modulate the electron density of the hydrazone linkage, with more electronically conjugated or anisotropically deshielding systems leading to downfield shifts [33]. Only a single set of signals was observed for both NH and CH=N protons in all compounds, indicating the presence of a single predominant solution isomer. This behavior is consistent with the preferential observation of the thermodynamically favored *E*-isomer, commonly reported for hydrazone derivatives [31].

The aromatic/heteroaromatic region further supported the assigned structures. In **3a**, the pyrrole ring exhibited signals at δ 6.11–6.96 ppm with J ≈ 4 Hz, consistent with vicinal coupling in substituted pyrrole systems. In **3c**, the thiazole protons appeared as doublets (J = 3.0 Hz), reflecting reduced coupling efficiency in a heteroaromatic ring containing adjacent N and S atoms [34]. For the fused systems, distinct deshielding effects were observed. In **3f**, the benzothiazole derivative, aromatic protons adjacent to the fused heterocycle resonated at δ 8.16–8.05 ppm, consistent with the electron-deficient nature and anisotropic deshielding of the benzothiazole ring [35]. A similar but less pronounced effect was observed for the benzimidazole derivative (**3e**), where the presence of two nitrogen atoms within the fused heteroaromatic system contributes to electron withdrawal and deshielding, as reflected in the relatively downfield azomethine (δ 8.54 ppm) and N-methyl (δ 4.07 ppm) signals [32]. The substituent methyl groups were clearly identified: OCH_3_ at δ 3.79–3.87 ppm and CH_3_ at δ 2.28–2.33 ppm. In N-methyl-substituted derivatives (**3a**, **3b**, **3d**, **3e**), the NCH_3_ signals appeared at δ 3.78–4.07 ppm, with the most downfield value in **3e**, consistent with increased electron withdrawal in the benzimidazole system. Overall, the ^1^H NMR data confirm the proposed structures and reveal clear substituent-dependent electronic effects within the hydrazone framework.

#### 2.2.3. APT-^13^C NMR Spectroscopy

The APT-^13^C NMR spectra of compounds **3a**–**3f** were consistent with the proposed hydrazone structures, enabling differentiation of protonated and quaternary carbons and further supporting the formation of the conjugated system (see Figures S13–S18 in the Supporting Information). A characteristic signal corresponding to the amide carbonyl (C=O) carbon was observed in the range of δ 159.39–165.76 ppm, in agreement with reported values for conjugated hydrazone derivatives [24,31]. The slight downfield shift observed in sulfur-containing derivatives (**3c** and **3f**) is consistent with increased conjugation and electron-withdrawing effects of the heteroaromatic substituents [36]. Signals attributed to azomethine and aromatic sp^2^ carbons were distributed over the range of δ ~110–150 ppm, reflecting the conjugated hydrazone–heteroaromatic framework. The ipso carbon of the methoxy-substituted aromatic ring was consistently observed at δ ~154–155 ppm, confirming the presence of the anisole moiety across the series. The methoxy carbon (OCH_3_) resonated in a narrow range of δ 53.6–56.8 ppm, while N-methyl carbons (when present) appeared at δ ~29–36 ppm. The pyrazole methyl group gave a consistent signal at δ ~10–11 ppm in all compounds. Overall, the APT-^13^C NMR data are in good agreement with literature values for structurally related hydrazone systems and provide further confirmation of the proposed molecular structures.

#### 2.2.4. HRMS Analysis

The molecular formulas of compounds **3a**–**3f** were further supported by high-resolution mass spectrometry (HRMS, ESI, positive mode). The experimentally observed protonated molecular ions [M+H]^+^ were in excellent agreement with the calculated exact masses. For instance, **3a** showed a peak at *m*/*z* 338.16151 (calcd 338.16115), while **3f** was observed at *m*/*z* 392.11735 (calcd 392.11757). All measured masses were within ±5 ppm error, indicating high mass accuracy and providing strong support for the proposed molecular formulas. The complete HRMS data for compounds **3a**–**3f**, including the expanded molecular-ion spectra (Figures S19–S24) and complete HRMS peak-list data (Tables S1–S6), are provided in the Supporting Information. The observed agreement between calculated and experimental exact masses is consistent with standard HRMS-based molecular formula verification in electrospray ionization analyses and with reports on structurally related hydrazone derivatives [36,37,38].

### 2.3. In Vitro Biological Activity Evaluation

The biological potential of the synthesized pyrazole-based hydrazone derivatives (**3a**–**3f**) was investigated through a combination of antioxidant, photoprotective, CT-DNA-associated spectroscopic, and cell-based assays. The design of these compounds is based on the condensation of a carbohydrazide scaffold with structurally diverse heteroaromatic aldehydes (N-methylpyrrole, N-methylimidazole, thiazole, N-methylindole, N-methylbenzimidazole, and benzothiazole), aiming to generate conjugated hydrazone systems with modulated electronic properties. The incorporation of different heteroaromatic moieties is expected to influence electron distribution, resonance stabilization, and consequently the radical scavenging capacity of the resulting hydrazone framework.

The antioxidant activities of compounds **3a**–**3f** were evaluated using DPPH radical scavenging, ferrous ion chelation, and ferric thiocyanate (FTC) total antioxidant capacity assays. In each assay, two standard reference compounds were included for comparison. For antioxidant assays, measurements were performed at concentrations ranging from 0.5 to 10.0 µg/mL, in triplicate, and the results are expressed as mean ± standard deviation (SD). The dose-dependent behavior of the compounds was also statistically evaluated.

The photoprotective potential of the compounds was assessed using the same concentration range (0.5–10.0 µg/mL) using the spectrophotometric sun protection factor (SPF) method, with carrot seed oil employed as a reference standard.

CT-DNA-associated spectroscopic responses were further evaluated using UV-Vis absorption and fluorescence spectroscopy. The compounds were studied at fixed concentrations in the range of 25–45 µM in the presence of increasing CT-DNA concentrations in order to monitor DNA-associated spectral changes under physiologically relevant buffer conditions.

In addition, the biological effects of the compounds were evaluated in A431 human epidermoid carcinoma cells and HaCaT immortalized human keratinocytes by cell viability (MTT) and wound closure assays to assess their potential cytotoxic and migration-associated cellular responses.

#### 2.3.1. DPPH Radical Scavenging Activity Assay

**Figure 1:**
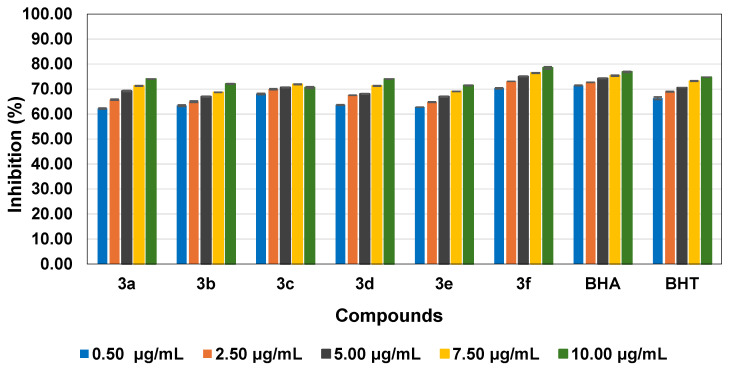
In vitro DPPH radical scavenging activities (% inhibition) of compounds **3a**–**3f** at different concentrations (0.50–10.00 µg/mL), compared with **BHA** and **BHT** as reference antioxidants. Data are expressed as mean ± standard deviation (SD) of three replicate measurements (*n* = 3). Error bars represent SD values. Statistical analysis was performed using two-way ANOVA followed by Tukey’s HSD post hoc test. Detailed statistical groupings are provided in Table S2.

The DPPH radical scavenging activities of compounds **3a**–**3f** were evaluated at 0.50–10.00 µg/mL using butylated hydroxyanisole (BHA) and butylated hydroxytoluene (BHT) as reference antioxidants (Figure 1, Table S8). The tested concentrations (0.5–10.0 µg/mL) correspond to approximately 1.1–8.7 µM (0.0011–0.0287 mM), depending on the molecular weight of each derivative; detailed conversions are provided in Table S7 (Supporting Information). All tested derivatives exhibited pronounced radical scavenging activity throughout the studied range, with inhibition values already exceeding 60% at the lowest concentration, indicating strong intrinsic antioxidant capacity. Accordingly, IC_50_ values could not be determined within the selected concentration window. Two-way ANOVA performed on arcsine-transformed inhibition data revealed significant effects of compound [*F*(7, 80) = 3792.68, *p* < 0.0001], concentration [*F*(4, 80) = 6881.06, *p* < 0.0001], and their interaction [*F*(28, 80) = 116.61, *p* < 0.0001], demonstrating that the magnitude of the concentration-dependent response varied across the tested structures. Because several groups exhibited very low within-group variance, one-way ANOVA followed by Tukey’s HSD test was additionally applied at each concentration level to further resolve pairwise differences between groups. Statistically significant differences between groups were observed at all concentration levels (*p* < 0.0001). At 10.00 µg/mL, compound **3f** displayed the highest DPPH scavenging activity (78.50 ± 0.48%), followed by **BHA** (76.91 ± 0.19%) and **BHT** (74.81 ± 0.00%). Compounds **3a** and **3d** showed identical mean inhibition values (74.05 ± 0.00%), whereas **3b** (72.07 ± 0.11%), **3e** (71.44 ± 0.11%), and **3c** (70.61 ± 0.33%) were less active. Tukey’s HSD test indicated that **3f** was significantly more active than all other tested groups, including the reference antioxidants, while **3a** and **3d** did not differ significantly from each other. Based on the highest tested concentration, the activity order was: **3f** > **BHA** > **BHT** > **3a** = **3d** > **3b** > **3e** > **3c**.

DPPH scavenging in this series can be interpreted through hydrogen atom transfer (HAT) and single electron transfer (SET) mechanisms, modulated by electronic structure and substituent effects [39,40,41,42]. The hydrazone core provides a conjugated framework for radical stabilization; however, activity is strongly influenced by the attached heteroaromatic system [43]. The superior performance of **3f** is attributed to the benzothiazole moiety, which enhances π-conjugation and introduces a polarizable sulfur atom that facilitates electron donation and stabilizes radical intermediates [44,45,46]. The thiazole derivative **3c** shows comparatively lower activity at higher concentration, suggesting limited delocalization relative to the fused benzothiazole system [47]. In contrast, N-methylated derivatives (**3a**, **3b**, **3e**) exhibit reduced activity, likely due to diminished lone-pair participation and hydrogen-donating capacity [48,49]. The indole-containing compound **3d** displays intermediate behavior, consistent with partial stabilization by its π-system but reduced effectiveness due to N-methyl substitution [50]. Overall, these results indicate that sulfur-containing, highly conjugated heteroaromatic systems enhance radical scavenging efficiency, whereas N-methylation generally attenuates antioxidant performance.

#### 2.3.2. Fe^2+^ Chelating Activity Assay

**Figure 2:**
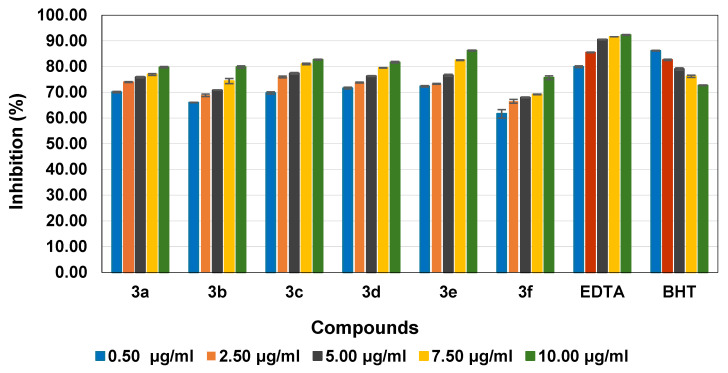
In vitro Fe^2+^ chelating activities (% inhibition) of compounds **3a**–**3f** at different concentrations (0.50–10.00 µg/mL), compared with **EDTA** and **BHT** as reference compounds. Data are expressed as mean ± standard deviation (SD) of three replicate measurements (*n* = 3). Error bars represent SD values. Statistical analysis was performed using two-way ANOVA followed by Tukey’s HSD post hoc test. Detailed statistical groupings are provided in Table S3.

The ferrous ion (Fe^2+^) chelating activities of compounds **3a**–**3f** were evaluated at 0.50–10.00 µg/mL using EDTA and BHT as reference compounds (Figure 2, Table S9). All synthesized derivatives exhibited concentration-dependent increases in chelating activity, indicating their ability to interfere with Fe^2+^-ferrozine complex formation and thereby inhibit metal-catalyzed oxidative processes. Two-way ANOVA performed on arcsine-transformed inhibition data revealed significant effects of compound [*F*(7, 80) = 3474.85, *p* < 0.0001], concentration [*F*(4, 80) = 1881.34, *p* < 0.0001], and their interaction [*F*(28, 80) = 295.47, *p* < 0.0001], demonstrating that the magnitude of the concentration-dependent response varied across the tested structures. Because several groups exhibited very low within-group variance, one-way ANOVA followed by Tukey’s HSD test was additionally applied at each concentration level to further resolve pairwise differences between groups. Statistically significant between-group differences were observed at all concentration levels (*p* < 0.0001). At 10.00 µg/mL, compound **3e** displayed the highest Fe^2+^ chelating activity among the synthesized derivatives (86.24 ± 0.20%), followed by **3c** (82.71 ± 0.17%) and **3d** (81.68 ± 0.30%). Compounds **3b** (79.85 ± 0.43%) and **3a** (79.74 ± 0.26%) showed comparable moderate activity, whereas **3f** exhibited lower chelating capacity (75.68 ± 0.75%). As expected, **EDTA** demonstrated the highest activity overall (92.29 ± 0.17%), while **BHT** showed the lowest value at this concentration (72.72 ± 0.10%). Tukey’s HSD analysis indicated that **3e** was significantly more active than all other synthesized derivatives, while **3a** and **3b** did not differ significantly from each other. Based on the highest tested concentration, the activity order was: **EDTA** > **3e** > **3c** > **3d** > **3b** ≈ **3a** > **3f** > **BHT**.

From a mechanistic perspective, the Fe^2+^ chelation assay differs fundamentally from radical scavenging assays, as it reflects the ability of a ligand to compete with ferrozine for Fe^2+^ binding rather than to quench a free radical directly [40]. Accordingly, chelating efficiency is governed mainly by the identity, number, and spatial arrangement of donor atoms. The superior performance of **3e** can be rationalized by the benzimidazole moiety, which provides nitrogen-rich donor sites favorable for Fe^2+^ coordination. This interpretation is consistent with the Hard and Soft Acids and Bases concept, according to which Fe^2+^, as a borderline acid, generally shows favorable interactions with nitrogen- and oxygen-donor ligands [51]. The hydrazone framework itself may also contribute through bidentate coordination via imine nitrogen and carbonyl oxygen, and potentially stronger chelation when additional heteroatom donors are suitably positioned [52]. In contrast, the lower activity of the benzothiazole derivative **3f** suggests that sulfur-containing heteroaromatic systems, although advantageous for DPPH radical stabilization, are less effective for Fe^2+^ coordination under the present assay conditions. The decreasing response of BHT with increasing concentration further supports its limited true chelating ability and suggests assay interference or weak coordination rather than efficient metal binding [53,54].

#### 2.3.3. Total Antioxidant Capacity Assay (FTC Method)

**Figure 3:**
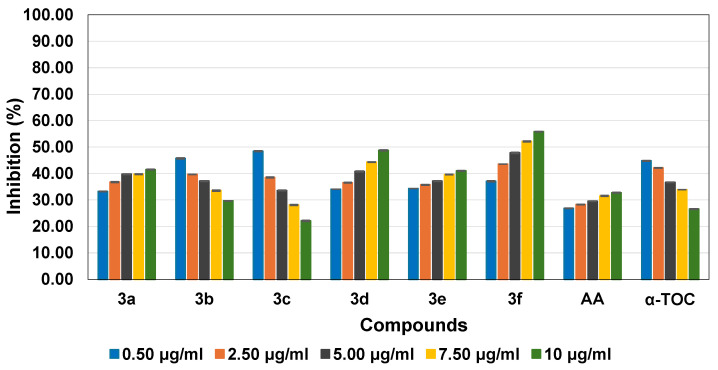
In vitro total antioxidant capacities (% inhibition) of compounds **3a**–**3f** determined by the FTC method at 36 h in a linoleic acid emulsion system, at different concentrations (0.50–10.00 µg/mL), compared with **AA** and **α-Toc** as reference antioxidants. Data are expressed as mean ± standard deviation (SD) of three replicate measurements (*n* = 3). Error bars represent SD values. Statistical analysis was performed using two-way ANOVA followed by Tukey’s HSD post hoc test. Detailed statistical groupings are provided in Table S4. Higher inhibition values indicate stronger suppression of lipid hydroperoxide formation.

The total antioxidant capacities of compounds **3a**–**3f** were evaluated by the ferric thiocyanate (FTC) method in a linoleic acid emulsion system, using ascorbic acid (**AA**) and α-tocopherol (**α-Toc**) as reference antioxidants (Figure 3, Tables S10 and Figure S25). In this assay, inhibition values reflect the suppression of primary lipid hydroperoxide formation at the selected time point (36 h). All tested compounds exhibited measurable antioxidant activity; however, their concentration-dependent behavior varied across the series. In particular, compounds **3d** and **3f** showed increasing inhibition with concentration, whereas **3b**, **3c**, and **α-Toc** displayed decreasing trends at higher doses. Two-way ANOVA performed on arcsine-transformed inhibition data revealed significant effects of compound [*F*(7, 80) = 21,739.24, *p* < 0.0001], concentration [*F*(4, 80) = 184.99, *p* < 0.0001], and their interaction [*F*(28, 80) = 7394.88, *p* < 0.0001], demonstrating that the FTC response strongly depends on both structural features and concentration. One-way ANOVA followed by Tukey’s HSD test revealed statistically significant differences among the tested groups (*p* < 0.0001). At 10.00 µg/mL, compound **3f** exhibited the highest total antioxidant capacity (55.88 ± 0.09%), followed by **3d** (48.79 ± 0.14%). Compounds **3a** (41.51 ± 0.12%) and **3e** (41.02 ± 0.12%) showed comparable intermediate activity and did not differ significantly from each other, whereas **AA** (32.77 ± 0.12%), **3b** (29.66 ± 0.08%), **α-Toc** (26.58 ± 0.12%), and **3c** (22.11 ± 0.16%) were less active. Tukey’s HSD analysis indicated that **3f** was significantly more active than all other groups. The activity order at the highest tested concentration was: **3f** > **3d** > **3a** ≈ **3e** > **AA** > **3b** > **α-Toc** > **3c**.

Time-dependent absorbance profiles at 10 µg/mL confirmed these findings. The control exhibited the characteristic increase in absorbance up to 36 h, followed by a decline at later time points, reflecting the formation and subsequent decomposition of primary lipid hydroperoxides. Under identical conditions, **3f** and **3d** consistently showed the lowest absorbance values, indicating the strongest inhibition of lipid peroxidation, whereas **3c** remained the least effective (Figure 3, Table S11). Mechanistically, the FTC assay differs from radical-scavenging and metal-chelation assays because it evaluates the inhibition of lipid peroxidation in a heterogeneous system. Therefore, antioxidant performance is governed not only by redox properties but also by lipophilicity, phase distribution, and the ability to interrupt chain propagation in the lipid phase [55,56]. Within this context, the superior activity of **3f** can be attributed to the benzothiazole moiety, which provides extended conjugation and favorable lipid-phase compatibility, enabling efficient chain-breaking antioxidant activity. The strong performance of **3d** suggests that the indole scaffold also supports effective stabilization of radical intermediates in lipid environments. In contrast, the relatively weak activity of **3c** indicates that thiazole-containing derivatives, although effective in radical scavenging assays, are less efficient in suppressing lipid peroxidation under FTC conditions. The decreasing trend observed for **α-Toc** at higher concentrations is consistent with literature reports describing concentration-dependent antioxidant/prooxidant balance in metal-containing lipid systems [57,58,59].

The present findings are consistent with literature reports demonstrating that hydrazone-based and heteroaromatic conjugated systems can effectively suppress lipid peroxidation, particularly when electron delocalization is combined with favorable phase distribution properties [60]. In this context, the superior FTC performance of compounds **3f** and **3d** suggests that benzothiazole- and indole-containing hydrazones are better suited for inhibiting lipid-phase oxidative propagation than more polar or less effectively conjugated analogs. Overall, these results indicate that FTC activity in this series is governed not solely by donor atom chemistry but also by the combined effects of conjugation, aromatic stabilization, and lipid-phase compatibility.

##### Comparative Analysis of Antioxidant Assays and Structure–Activity Relationships

The antioxidant properties of the synthesized pyrazole-based carbohydrazone derivatives (**3a**–**3f**) were systematically evaluated using three complementary in vitro assays: DPPH radical scavenging, Fe^2+^ ion chelation, and total antioxidant capacity (FTC method). Although all compounds exhibited measurable activity across the three systems, their activity profiles diverged depending on the assay type, reflecting the fundamentally distinct underlying mechanisms.

In the DPPH assay, compound **3f** (benzothiazole-containing derivative) exhibited the highest radical scavenging activity, followed by **3a**/**3d**, whereas **3c** showed comparatively lower performance. In contrast, Fe^2+^ chelation results revealed a different trend, with **3e** (benzimidazole-containing derivative) displaying the strongest chelating activity among the synthesized compounds, approaching that of **EDTA**. Similarly, in the FTC assay, which evaluates inhibition of lipid peroxidation, **3f** and **3d** were identified as the most effective compounds, while **3c** remained the least active at higher concentrations. These differences can be rationalized by the distinct chemical principles governing each assay. The DPPH method primarily reflects hydrogen atom transfer (HAT) and single electron transfer (SET) mechanisms, favoring π-conjugated systems capable of stabilizing radical intermediates. In this context, the superior performance of **3f** is consistent with the benzothiazole scaffold, which enhances electron delocalization and stabilizes radical species through resonance involving both nitrogen and sulfur atoms. This behavior is well supported by literature reports on benzothiazole-based hydrazones with high radical scavenging efficiency [44,45].

In contrast, Fe^2+^ chelation is governed by coordination chemistry rather than redox reactivity. The hydrazone core (-C=N-NH-C(=O)-) provides bidentate coordination sites, while heteroaromatic substituents contribute additional donor atoms. The dominance of **3e** in this assay is therefore consistent with the benzimidazole moiety, which offers two nitrogen donor atoms and favorable coordination geometry. Similar structure–activity relationships have been reported for benzimidazole-based hydrazones and related ligands exhibiting strong Fe^2+^ binding capacity [50,52].

The FTC assay evaluates the inhibition of lipid peroxidation in a heterogeneous system and is therefore strongly influenced by lipophilicity and phase distribution. Under these conditions, compound **3f** exhibited the highest inhibitory activity, followed by **3d**, indicating an enhanced ability to suppress chain propagation in the lipid phase. Compounds **3a** and **3e** showed moderate activity, whereas **3b** and **3c** were less effective. This behavior can be attributed to the conjugated aromatic structures of these derivatives and their improved compatibility with lipid environments, which facilitate interactions with lipid radicals. Such findings are consistent with reports showing that conjugated hydrazone derivatives containing heteroaromatic systems effectively inhibit lipid peroxidation [55,60].

A preliminary Pearson correlation analysis based on the 10 µg/mL data (Table S13) revealed a strong positive correlation between DPPH and FTC activities (*r* = 0.854, *p* = 0.030), whereas Fe^2+^ chelation showed an inverse correlation with DPPH activity (*r* = −0.821, *p* = 0.045). These observations further support that radical scavenging/lipid peroxidation inhibition and metal-chelation processes are governed by partially distinct structural determinants in this compound series. Due to the limited dataset (*n* = 6), these correlations should be interpreted as preliminary structure–activity indicators rather than statistically robust generalizations. Importantly, no single compound dominated across all antioxidant mechanisms. While **3f** showed strong performance in both DPPH and FTC assays, it exhibited comparatively lower Fe^2+^ chelating ability. Conversely, **3e** was highly effective in metal chelation but showed only moderate activity in radical scavenging and lipid peroxidation systems. This divergence highlights the inherently multidimensional nature of antioxidant activity and underscores the necessity of employing multiple complementary assays.

Overall, the structure–activity relationship emerging from this study indicates that sulfur-containing heterocycles (benzothiazole) enhance radical scavenging and lipid-phase antioxidant activity, whereas nitrogen-rich heterocycles (benzimidazole) favor metal coordination processes. More broadly, antioxidant performance in this series is governed by the interplay of conjugation, donor atom availability, and physicochemical compatibility with the reaction environment, rather than a single dominant structural feature.

#### 2.3.4. Photoprotective Activity Assay

**Figure 4:**
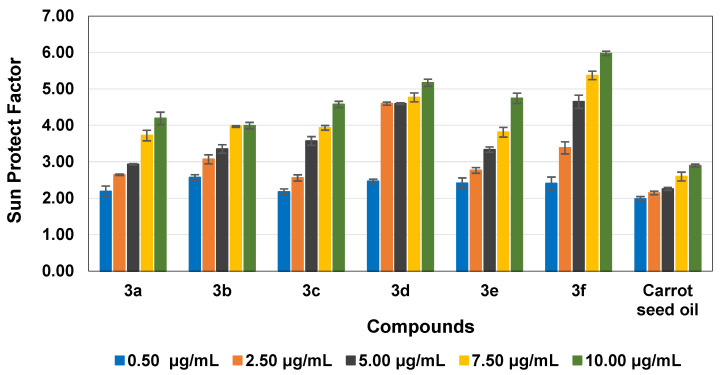
In vitro sun protection factor (SPF) values of compounds **3a**–**3f** and carrot seed oil (0.50–10.00 µg/mL), determined by the Mansur UV spectrophotometric method. Values represent mean ± standard deviation (*n* = 3). Statistical differences among groups were evaluated by one-way ANOVA followed by Tukey’s HSD test (*p* < 0.05).

The photoprotective properties of the synthesized pyrazole-based carbohydrazone derivatives (**3a**–**3f**) were evaluated by determining their sun protection factor (SPF) values using the Mansur spectrophotometric method in the UV-B region (290–320 nm) (Figure 4, Table S12). All compounds showed concentration-dependent increases in SPF, indicating progressively stronger UV absorption at higher concentrations. Two-way ANOVA revealed significant effects of compound [*F*(6, 70) = 571.79, *p* < 0.0001], concentration [*F*(4, 70) = 1439.71, *p* < 0.0001], and their interaction [*F*(24, 70) = 41.41, *p* < 0.0001], demonstrating that the SPF response was strongly dependent on both structure and dose. One-way ANOVA followed by Tukey’s HSD test revealed statistically significant differences among the tested groups at all concentrations (*p* < 0.001). At 10.00 µg/mL, compound **3f** displayed the highest SPF value (5.98 ± 0.06), followed by **3d** (5.17 ± 0.10), **3e** (4.74 ± 0.14), and **3c** (4.57 ± 0.09). Compounds **3a** (4.19 ± 0.17) and **3b** (3.99 ± 0.09) showed lower but still measurable photoprotective activity and did not differ significantly from each other, whereas carrot seed oil gave the lowest SPF value (2.89 ± 0.04). Based on the highest tested concentration, the SPF ranking was: **3f** > **3d** > **3e** > **3c** > **3a** ≈ **3b** > carrot seed oil. Tukey’s HSD analysis confirmed that 3f was significantly superior to all other tested groups at 10.00 µg/mL.

The Mansur method is a rapid and widely used in vitro approach that estimates SPF from UV absorbance data weighted across the erythemal UV-B range and is commonly applied for preliminary screening of photoprotective candidates [61,62]. In this context, the concentration-dependent increase observed for all synthesized derivatives is consistent with enhanced UV-B absorption arising from the conjugated hydrazone framework and heteroaromatic substituents. Classical spectroscopic studies on hydrazones have shown that their UV absorption is governed by π-π* and n-π* transitions, and that heteroatoms and conjugation can shift absorption toward longer wavelengths and increase band intensity [63,64]. From a structure–property perspective, the superior SPF performance of **3f** and **3d** suggests that benzothiazole and N-methylindole substituents provide the most favorable electronic delocalization and UV absorption characteristics within this series. The presence of sulfur in the benzothiazole ring (**3f**) may further enhance polarizability and contribute to broader and more intense absorption, consistent with reports describing benzothiazole derivatives as multifunctional antioxidant and photoprotective agents [44]. Similarly, indole-containing systems have been associated with effective UV absorption due to their extended π-electron systems and favorable electronic distribution [65].

Related hydrazone-based systems further support this interpretation. Benzofuran, benzimidazole, and indole hydrazones have been reported to exhibit meaningful photoprotective activity, with some derivatives showing UV-filter performance comparable to conventional sunscreen candidates [66,67]. In line with these findings, the present results highlight that heteroaromatic substitution combined with a conjugated hydrazone bridge plays a critical role in enhancing UV absorption efficiency. To the best of our knowledge, photoprotective studies on pyrazole-based carbohydrazones bearing the present heteroaromatic substitution pattern remain limited. The closest precedents concern related hydrazone families rather than this specific scaffold. Therefore, the present results extend photoprotective hydrazone chemistry into a less-explored pyrazole-based carbohydrazone framework and suggest that this scaffold may represent a promising platform for the development of multifunctional UV-absorbing agents.

The relatively low SPF values observed for carrot seed oil are consistent with previous reports, where plant-derived oils evaluated alone generally exhibit modest UV-B protection and are more appropriately considered as auxiliary components rather than primary UV filters [62]. Taken together, these findings indicate that photoprotective behavior in this compound series is governed primarily by conjugation length, heteroatom-assisted electronic delocalization, and aromatic UV-absorbing character. Within this framework, compound **3f** emerges as the most promising photoprotective candidate, while **3d** and **3e** also demonstrate meaningful SPF enhancement. More broadly, these results position pyrazole-based carbohydrazones as structurally versatile candidates for the development of compounds combining UV-filtering potential with complementary antioxidant properties.

##### Relationship Between SPF and Antioxidant Parameters

To examine whether photoprotective behavior is associated with antioxidant performance, SPF values were compared with DPPH radical scavenging, Fe^2+^ chelation, and FTC total antioxidant capacity using the 10.00 µg/mL data obtained for the six synthesized derivatives (**3a**–**3f**). Reference standards were excluded because they represent chemically distinct comparators across the different assays.

A Pearson correlation analysis showed that DPPH and FTC were positively correlated (*r* = 0.854, *p* = 0.030), whereas DPPH and Fe^2+^ chelation showed a statistically significant negative correlation (*r* = −0.821, *p* = 0.045). By contrast, FTC and Fe^2+^ chelation were only weakly and non-significantly related (*r* = −0.431, *p* = 0.393). SPF showed moderate but non-significant positive correlations with both DPPH (*r* = 0.751, *p* = 0.085) and FTC (*r* = 0.751, *p* = 0.085), while it was weakly and inversely related to Fe^2+^ chelation (*r* = −0.394, *p* = 0.440). Due to the small dataset (*n* = 6), these correlations should be interpreted as preliminary indicators of structure–activity trends rather than statistically robust generalizations. Pearson correlation coefficients are summarized in Table S13.

These relationships are chemically plausible and consistent with the observed structure–activity trends. The positive DPPH-FTC association is consistent with the strong activity of compound **3f** in both assays, suggesting that structural features favoring radical stabilization may also enhance inhibition of lipid-phase oxidation. In contrast, the inverse relationship between DPPH and Fe^2+^ chelation reflects the different structural requirements of the two assays: sulfur-containing, highly conjugated systems favor radical scavenging, whereas nitrogen-rich donor environments favor metal coordination [44,45,50,52].

The SPF correlations further indicate that UV protection in this series is only partially aligned with antioxidant behavior, with moderate but non-significant positive correlations observed for DPPH and FTC. This is consistent with the broader literature, which emphasizes that SPF primarily reflects UV absorption, whereas antioxidant assays probe redox or coordination processes; overlap is possible but not obligatory [61,62,68,69].

Accordingly, the present compounds exhibit partially overlapping functional tendencies. Compounds **3f** and **3d** perform strongly in redox- and lipid peroxidation-related antioxidant systems, while **3f**, **3d**, and **3e** also display the highest SPF values within the series, with **3f** representing the most balanced multifunctional profile. In contrast, compound **3e** is distinguished primarily by its strong Fe^2+^ chelation capacity rather than by dominance in radical scavenging or photoprotective performance. Taken together, these findings support the conclusion that photoprotective capacity and antioxidant activity are related but mechanistically non-equivalent properties, and that their co-optimization depends on scaffold-specific electronic and physicochemical characteristics rather than a single universal descriptor.

#### 2.3.5. UV-Vis Spectroscopic Response of Compounds **3a**–**3f** in the Presence of CT-DNA

The spectroscopic responses of compounds **3a**–**3f** in the presence of calf thymus DNA (CT-DNA) were investigated by UV-Vis absorption spectroscopy in Tris-HCl buffer (10 mM, pH 7.4) containing 50 mM NaCl. The concentration of CT-DNA was determined spectrophotometrically at 260 nm using an extinction coefficient of 6600 M^−1^ cm^−1^. DNA concentrations reported throughout this study are expressed as nucleotide phosphate (base) concentrations rather than base-pair concentrations [70]. Absorption spectra were recorded following incremental addition of CT-DNA (0–15 μM) to fixed concentrations of the compounds (25–45 μM), and an equilibration period of 35 min was allowed before each measurement.

UV-Vis spectroscopy is widely employed as an initial method for monitoring spectral perturbations observed in the presence of DNA. However, absorption changes alone are generally insufficient for rigorous determination of binding modes and typically require complementary biophysical techniques such as circular dichroism, viscosity measurements, or thermal denaturation studies for mechanistic confirmation [18,71,72]. Upon incremental addition of CT-DNA, most compounds exhibited measurable changes in their absorption spectra, indicating that the presence of CT-DNA was associated with measurable changes in the absorption spectra of the pyrazole-based carbohydrazone scaffold. The observed responses differed among the derivatives, indicating that heteroaromatic substitution influences the spectroscopic behavior of the compounds in the presence of CT-DNA (Table 1). For compounds **3a** and **3b**, the principal absorbance changes occurred within the intrinsic CT-DNA absorption region (240–250 nm), where the contribution of DNA itself precluded reliable interpretation of compound-specific spectral responses. Although minor absorbance changes were observable at wavelengths above 300 nm (Figures S26 and S27), these responses were considered insufficient for meaningful comparative evaluation. Consequently, these derivatives were excluded from the comparative qualitative analysis presented in Table 1.

Compound **3c** exhibited a modest increase in absorbance with minimal wavelength displacement. In contrast, compounds **3d** and **3f** displayed pronounced decreases in absorbance accompanied by bathochromic shifts, whereas compound **3e** showed an increase in absorbance with only minor wavelength changes. These observations indicate that the heteroaromatic substituent substantially influences the optical response of the carbohydrazone framework in the presence of CT-DNA.

Among the series, compounds **3d** and **3f** exhibited the most pronounced spectral changes following CT-DNA addition. The larger aromatic frameworks present in these derivatives may contribute to the observed spectroscopic responses; however, this interpretation remains tentative because no independent measurements were performed to directly evaluate the contribution of molecular planarity, aggregation behavior, or binding stoichiometry under the experimental conditions employed. Accordingly, the UV-Vis results are discussed in terms of CT-DNA-induced spectral changes rather than quantitative binding parameters or specific interaction modes.

**Figure 5:**
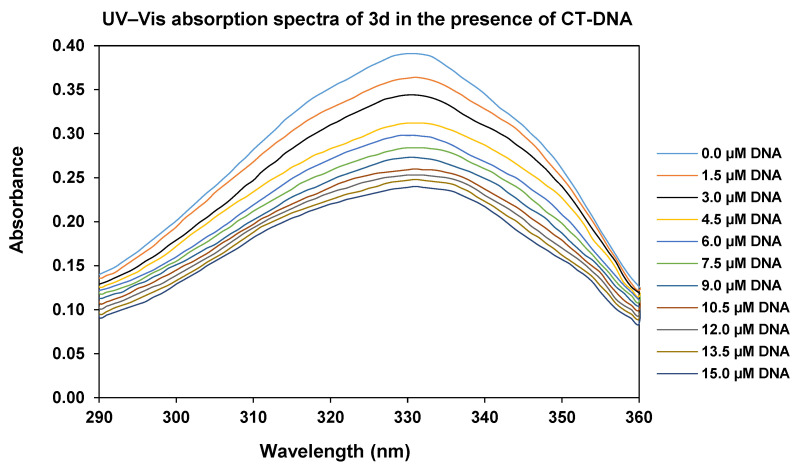
UV-Vis absorption spectra of compound **3d** recorded upon incremental addition of CT-DNA (0.0–15.0 µM) in Tris-HCl buffer (10 mM, pH 7.4) containing 50 mM NaCl at 37 °C. The concentration of **3d** was fixed at 25 µM. A concentration-dependent decrease in absorbance accompanied by a bathochromic shift was observed upon increasing CT-DNA concentration.

**Figure 6:**
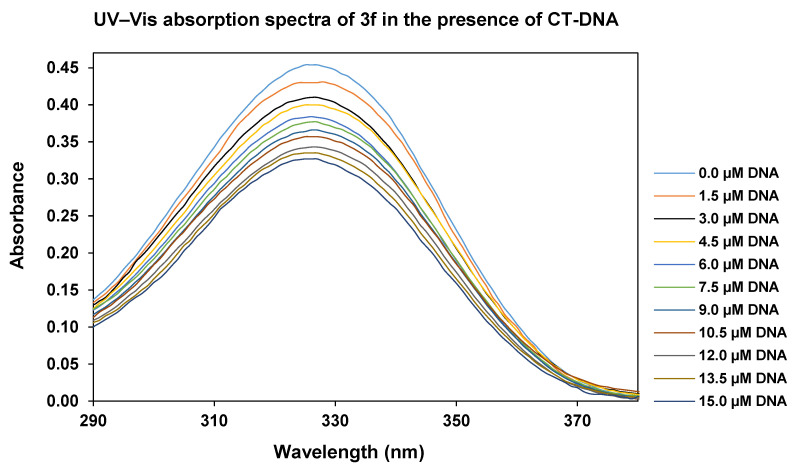
UV-Vis absorption spectra of compound **3f** recorded upon incremental addition of CT-DNA (0.0–15.0 µM) in Tris-HCl buffer (10 mM, pH 7.4) containing 50 mM NaCl at 37 °C. The concentration of **3f** was fixed at 25 µM. A concentration-dependent decrease in absorbance accompanied by a bathochromic shift was observed upon increasing CT-DNA concentration.

In light of the limitations associated with the DNA concentration range employed in the present study and the absence of saturation behavior, quantitative binding constants and binding-mode assignments were not derived from the UV-Vis data. Therefore, the results are presented as qualitative spectroscopic evidence of CT-DNA-associated absorbance changes rather than definitive measurements of quantitative DNA interaction parameters. Representative spectra for compounds **3d** and **3f** are shown in Figure 5 and Figure 6, respectively, while the complete spectral dataset for all compounds is provided in the Supporting Information (Figures S26–S31).

#### 2.3.6. Fluorescence Spectroscopic Response of Compounds **3a**–**3f** in the Presence of CT-DNA

Fluorescence spectroscopy was employed as a complementary technique to evaluate changes in the emission behavior of compounds **3a**–**3f** following incremental addition of calf thymus DNA (CT-DNA) under physiological buffer conditions (10 mM Tris-HCl, 50 mM NaCl, pH 7.4). Fluorescence spectroscopy is widely used as a sensitive method for monitoring ligand-induced changes in the local environment of fluorescent molecules. Fluorescence enhancement observed following DNA addition has been attributed to several possible factors, including reduced solvent exposure, restricted intramolecular motion, changes in fluorophore microenvironment, and other environment-dependent photophysical processes [18,73]. However, absorption and fluorescence spectroscopy provide primarily preliminary information regarding ligand–DNA interactions, and complementary techniques such as circular dichroism, viscosity measurements, or thermal denaturation studies are generally required for a more rigorous evaluation of interaction mechanisms and DNA conformational effects [71,72].

Because the excitation wavelengths employed for compounds **3a** and **3b** (240 and 248 nm, respectively) overlap with the intrinsic absorption region of CT-DNA, and because fluorescence measurements were performed at relatively high compound concentrations (25–45 μM), these datasets may be susceptible to inner-filter and self-absorption effects [73]. Therefore, fluorescence responses obtained for compounds **3a** and **3b** were not included in the comparative analysis and were interpreted only as qualitative observations.

**Figure 7:**
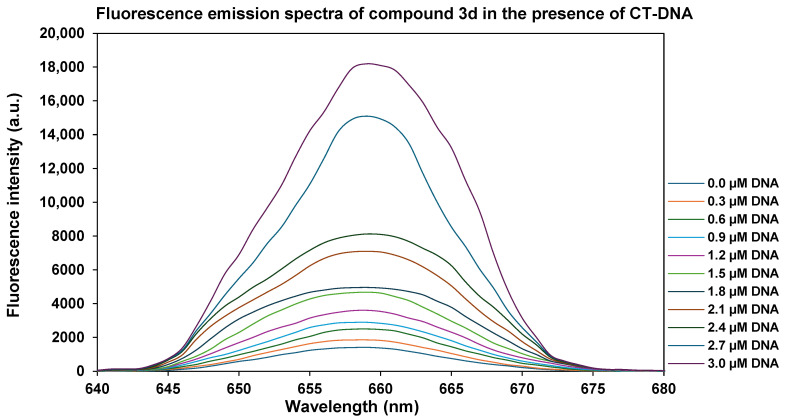
Fluorescence emission spectra of compound **3d** recorded upon incremental addition of CT-DNA (0.0–3.0 μM) in Tris-HCl buffer (10 mM, pH 7.4) containing 50 mM NaCl at 37 °C. The concentration of **3d** was fixed at 25 μM, and fluorescence emission was monitored around λ_em_ ≈ 659 nm following excitation at λ_ex_ = 330 nm. The spectra show pronounced fluorescence enhancement upon increasing CT-DNA concentration.

**Figure 8:**
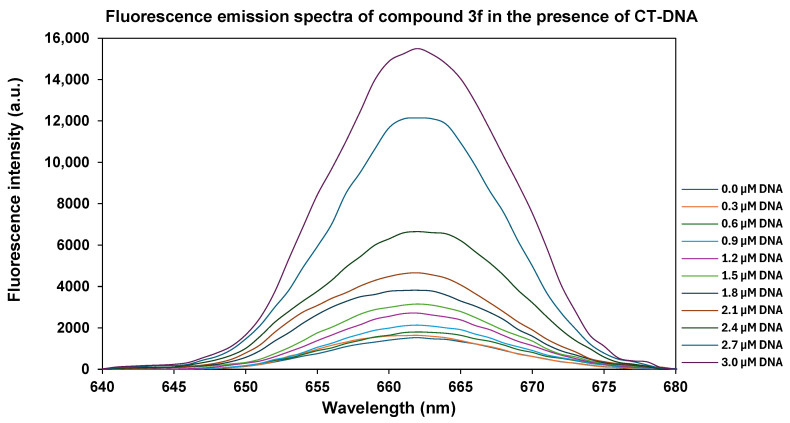
Fluorescence emission spectra of compound **3f** recorded upon incremental addition of CT-DNA (0.0–3.0 μM) in Tris-HCl buffer (10 mM, pH 7.4) containing 50 mM NaCl at 37 °C. The concentration of **3f** was fixed at 25 μM, and fluorescence emission was monitored around λ_em_ ≈ 662 nm following excitation at λ_ex_ = 330 nm. The spectra show pronounced fluorescence enhancement upon increasing CT-DNA concentration.

Representative fluorescence emission spectra of compounds **3d** and **3f** are presented in Figure 7 and Figure 8, respectively, while the complete fluorescence dataset for compounds **3c**–**3**f is provided in the Supporting Information (Figures S32–S35).

Upon incremental addition of CT-DNA, compounds **3c**–**3f** exhibited concentration-dependent fluorescence enhancement (F/F_0_ > 1), indicating that the experimental addition of CT-DNA was associated with measurable changes in the emission properties. The magnitude of the fluorescence response varied among the derivatives, as reflected by the maximum F/F_0_ values (Table 2). Compound **3c** showed a lower maximum F/F_0_ value (2.47), whereas compounds **3d**, **3e**, and **3f** showed higher maximum F/F_0_ values of 12.82, 9.01, and 10.13, respectively.

The fluorescence responses observed for compounds **3c**–**3f** indicate that heteroaromatic substitution affects the emission behavior of the carbohydrazone framework in the presence of CT-DNA. Among the evaluated derivatives, compounds **3d** and **3f** showed the largest relative fluorescence increases, whereas compound **3c** showed a comparatively smaller response. Compound **3e** displayed an intermediate behavior. The larger aromatic frameworks and greater electronic delocalization present in compounds **3d** and **3f** may contribute to the enhanced spectroscopic responses observed under the experimental conditions employed. These observations are consistent with the structure-dependent spectroscopic responses observed in the UV-Vis experiments.

Because fluorescence intensity changes may arise from multiple factors, including changes in local microenvironment, conformational restriction, aggregation phenomena, and DNA-associated interactions, the observed fluorescence responses should not be interpreted as direct measurements of binding affinity or specific binding mode [18,73]. Furthermore, the DNA concentration range employed in the present study did not achieve saturation conditions. Consequently, quantitative binding parameters were not derived from the fluorescence data, and the observed responses are presented as qualitative spectroscopic evidence of CT-DNA-associated fluorescence perturbation.

Overall, the fluorescence results demonstrate that CT-DNA influences the emission behavior of compounds **3c**–**3f** under the experimental conditions employed. Additional studies employing optimized titration ranges, saturation analysis, and complementary biophysical techniques would be required to establish quantitative binding parameters and elucidate the molecular origin of the observed fluorescence responses.

##### Relationship Between CT-DNA-Associated Spectroscopic Responses, Antioxidant Activity, and Photoprotective Properties

Comparison of the spectroscopic, antioxidant, and photoprotective data reveals several structure-dependent trends across the synthesized compound series. Although these observations do not imply a direct mechanistic relationship, compounds **3d** and **3f** exhibited both pronounced CT-DNA-associated spectroscopic responses and strong antioxidant performance across multiple assay systems.

Extended aromatic conjugation and heteroaromatic substitution have frequently been associated with altered electronic properties and diverse biological activities in hydrazone-based systems [8,74]. In the present series, the relatively large aromatic systems present in compounds **3d** and **3f** may contribute to both their favorable antioxidant performance and their pronounced spectroscopic responses in the presence of CT-DNA.

In contrast, Fe^2+^ chelation followed a different trend. Compound **3e** displayed comparatively strong metal-chelating activity despite exhibiting less pronounced spectroscopic responses than compounds **3d** and **3f**. This observation suggests that metal coordination and CT-DNA-associated spectroscopic behavior are governed by different structural factors. Whereas spectroscopic responses appear to be influenced largely by aromatic conjugation and electronic delocalization, metal-chelating activity is more strongly dependent on donor atom availability and coordination geometry.

The photoprotective results likewise showed only partial correspondence with the spectroscopic data. SPF performance appeared to be influenced predominantly by UV absorption characteristics rather than by antioxidant activity or CT-DNA-associated spectroscopic responses. This finding is consistent with the well-established dependence of photoprotective behavior on electronic absorption profiles and spectral coverage within the UV region.

Taken together, the results indicate that antioxidant activity, metal-chelating ability, photoprotective performance, and CT-DNA-associated spectroscopic responses represent interconnected yet mechanistically distinct properties. Although no direct causal relationships can be established from the present data, the observed trends suggest that aromatic conjugation, heteroatom composition, and electronic distribution collectively influence multiple physicochemical characteristics of the pyrazole-based carbohydrazone scaffold.

#### 2.3.7. Cell Viability Assays

The biological activity of the synthesized pyrazole-based carbohydrazone derivatives (**3a**–**3f**) was evaluated using MTT viability assays in A431 epidermoid carcinoma cells and HaCaT keratinocytes. Cell viability (%) was calculated by normalizing treated absorbance values to the corresponding untreated control within each cell line and exposure period. Representative viability profiles illustrating distinct cytotoxicity and selectivity patterns are presented in the main text, while complete dose- and time-dependent viability profiles for all compounds are provided in the Supplementary Materials (Figures S36–S41).

Across the series, treatment caused concentration- and time-dependent changes in metabolic viability, with higher concentrations causing stronger reductions in MTT signal. Several compounds also showed non-monotonic low-dose responses in which viability approached or exceeded control levels before declining at higher concentrations. This biphasic behavior is consistent with transient metabolic adaptation or hormetic-like responses preceding cytotoxic transition. Overall, compounds **3a**–**3f** differed in cytotoxic potency and selectivity toward A431 cells relative to HaCaT cells.

##### Overall Cytotoxic Trends and Comparative Activity

All synthesized derivatives exhibited measurable growth-inhibitory activity in both A431 and HaCaT cells, although response magnitude and timing varied among compounds. Three-way ANOVA identified concentration as the primary experimental factor associated with viability reduction of cytotoxic response across the series (*p* < 0.001 for all compounds), confirming progressive reductions in viability with increasing dose. Depending on the analog, significant effects of exposure time, cell line, and higher-order interactions were also seen, indicating that biological response depended on the combined experimental context rather than concentration alone (Table S14).

A common pattern involved mild low-dose metabolic stimulation followed by progressive cytotoxicity at higher concentrations and longer exposures. In several groups, especially A431 cells, low concentrations caused viability values near or slightly above control before marked reductions at intermediate and high doses. Similar non-monotonic profiles have been reported in tetrazolium-based assays for compounds that initially perturb mitochondrial metabolism or redox balance before progression toward reduced metabolic viability [75].

These findings should be interpreted in the context of the MTT assay, which measures tetrazolium reduction as an indicator of metabolic activity rather than direct cell number. Thus, transient increases in viability may reflect enhanced mitochondrial NAD(P)H-dependent reduction capacity or compensatory metabolic activation rather than true proliferation [75,76]. However, at higher concentrations, all compounds reduced MTT signal intensity, consistent with impaired metabolic viability and progressive cytotoxicity.

##### Differential Cellular Responses: A431 vs. HaCaT

Distinct response patterns were seen between malignant A431 cells and non-malignant HaCaT keratinocytes. A431 cells showed greater early-response variability, more frequent low-dose increases in metabolic viability, and earlier cytotoxicity at intermediate concentrations. These effects were especially evident for compounds **3a**, **3b**, and **3c,** which caused transient increases in viability before marked suppression at higher doses, possibly reflecting transient metabolic adaptation responses in carcinoma cells.

HaCaT cells generally showed more stable responses at lower concentrations and shorter exposures, with cytotoxicity becoming more evident after prolonged exposure or at higher concentrations. This differential sensitivity was most apparent for compounds **3a**, **3b**, and **3e**, which caused earlier and more pronounced suppression of A431 viability relative to HaCaT cells.

Despite these differences, high concentrations, especially 1 mM, consistently caused marked viability loss in both cell lines across nearly all compounds. Thus, although several analogs showed preferential cytotoxic activity under specific conditions, high-dose cytotoxicity was not exclusively cancer-selective. Selectivity depended strongly on compound structure, exposure duration, and concentration, supporting substituent-dependent modulation of biological response within the carbohydrazone scaffold series.

##### Compound-Specific Activity Profiles

**Figure 9:**
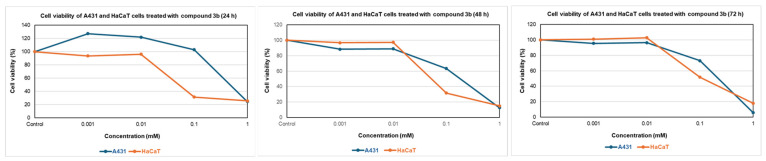
Effect of compound **3b** on cell viability in A431 epidermoid carcinoma cells and HaCaT immortalized keratinocytes after 24, 48, and 72 h exposure. Cell viability was determined using the MTT assay and expressed as percentage viability relative to the corresponding untreated control. Values are presented as mean ± SD (*n* = 4).

Among the tested compounds, compound **3b** demonstrated strong concentration-dependent cytotoxicity in both cell lines, with significantly greater suppression of A431 viability relative to HaCaT cells. Three-way ANOVA revealed significant effects of concentration (*p* < 0.0001), exposure time (*p* < 0.0001), and cell line (*p* = 0.0021), together with a significant concentration × cell line interaction (*p* = 0.0048). At 1 mM, compound **3b** produced significant viability reductions across all exposure periods in A431 cells (*p* < 0.0001). HaCaT cells also exhibited substantial viability loss at high concentration, although suppression remained less pronounced than in A431 cells during extended exposure. IC_50_ and *SI* analyses demonstrated improved selectivity with prolonged treatment, with the most favorable selectivity profile observed at 72 h. Among the tested compounds, **3b** exhibited one of the strongest combinations of cytotoxic potency and cancer-cell selectivity (Figure 9). Complete numerical datasets, including mean ± SD values derived from four independent replicates, are provided in Table S15 of the Supplementary Materials.

**Figure 10:**
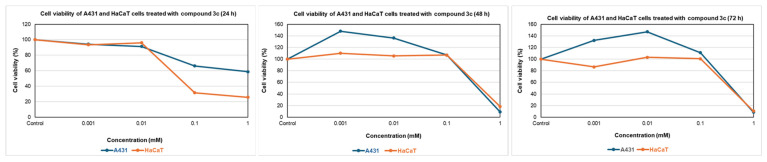
Effect of compound **3c** on cell viability in A431 epidermoid carcinoma cells and HaCaT immortalized keratinocytes after 24, 48, and 72 h exposure. Cell viability was determined using the MTT assay and expressed as percentage viability relative to the corresponding untreated control. Values are presented as mean ± SD (*n* = 4).

Compound **3c** displayed strong growth-inhibitory activity, with pronounced time- and concentration-dependent viability reductions, especially in A431 cells. Significant effects of concentration (*p* < 0.0001), exposure time (*p* < 0.0001), and cell line (*p* = 0.0017) were seen, together with a significant concentration × cell line interaction (*p* = 0.0032). The strongest effects occurred at 1 mM during 48–72 h exposure, where A431 viability approached near-background absorbance levels. HaCaT cells also showed significant viability loss at high concentration, although suppression remained greater in A431 cells. IC_50_ and SI analyses indicated moderate-to-high cancer-cell selectivity, with the most favorable selectivity window at 72 h. Overall, **3c** combined strong cytotoxic potency with sustained preferential activity (Figure 10).

**Figure 11:**
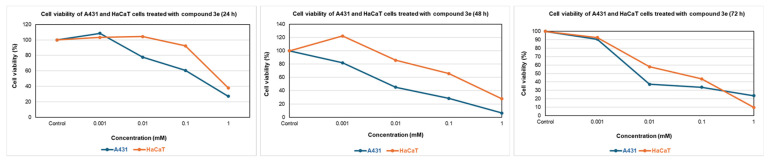
Effect of compound **3e** on cell viability in A431 epidermoid carcinoma cells and HaCaT immortalized keratinocytes after 24, 48, and 72 h exposure. Cell viability was determined using the MTT assay and expressed as percentage viability relative to the corresponding untreated control. Values are presented as mean ± SD (*n* = 4).

Compound **3e** exhibited pronounced concentration- and time-dependent cytotoxicity with variable selectivity across exposure periods. Three-way ANOVA showed significant effects of concentration (*p* < 0.0001), exposure time (*p* < 0.0001), and cell line (*p* = 0.0063), as well as a significant concentration × cell line interaction (*p* = 0.0027). The strongest cytotoxic effects were seen at 1 mM, especially during 48 h exposure, when A431 viability approached near-background levels in several replicates. IC_50_ and *SI* analyses showed the most favorable selectivity at 48 h, with strong preferential suppression of A431 cells relative to HaCaT cells. However, this selectivity was not maintained at later exposure periods, suggesting reduced selectivity during prolonged exposure and possible delayed HaCaT sensitivity. Overall, **3e** combined strong growth-inhibitory activity with context-dependent selectivity (Figure 11).

The remaining compounds (**3a**, **3d**, and **3f**) demonstrated comparable concentration-dependent cytotoxic behavior with varying degrees of selectivity and are presented in the Supplementary Materials (Figures S36–S41).

##### Structure–Activity Relationship (SAR) Interpretation

Comparative analysis of viability data, IC_50_ values, selectivity indices (*SIs*), and statistical response patterns identified several structure–activity relationship (SAR) trends across carbohydrazone derivatives (**3a**–**3f**). Although all compounds shared the same core scaffold, differences in cytotoxic potency, temporal activity, and cancer-cell selectivity indicated that heteroaromatic substituent variation influenced biological response. Several compounds showed preferential activity toward A431 cells, whereas others caused broader cytotoxicity in both malignant and non-malignant keratinocytes. These findings suggest that relatively small structural modifications altered the balance between growth-inhibitory potency and cancer-cell selectivity. The comparative profiles of compounds (**3a**–**3f**) are summarized in Table 3.

Activity classification was based on relative IC_50_ values and maximal reductions in viability across the tested concentration range and exposure periods. Selectivity was evaluated using *SI* = IC_50_(HaCaT)/IC_50_(A431), where *SI* > 1 indicates preferential cytotoxicity toward A431 cells. Qualitative descriptors such as moderate and strong reflect comparative trends within the compound series rather than fixed absolute thresholds.

Overall, compounds **3b** and **3c** showed the most consistent balance between cytotoxic potency and preferential activity toward A431 cells, especially during prolonged exposure. Compound **3a** displayed moderate but stable selectivity, whereas **3d** exhibited broader cytotoxicity toward both cell lines, reducing overall selectivity. Compound **3f** retained substantial growth-inhibitory activity but showed reduced selectivity because of concurrent HaCaT toxicity. Although **3e** exhibited favorable selectivity at 48 h, this effect was not maintained at 72 h, when increased HaCaT sensitivity markedly reduced the *SI*, indicating temporal instability.

The most favorable activity profiles were associated with compounds containing imidazole (**3b**), thiazole (**3c**), and benzimidazole (**3e**) substituents, suggesting that heterocycle identity contributed substantially to biological response modulation within the pyrazole–carbohydrazone scaffold. Compound **3b**, bearing an imidazole moiety, demonstrated one of the strongest combinations of cytotoxic potency and sustained preferential suppression of A431 cells during prolonged exposure. Imidazole-containing scaffolds are widely recognized in medicinal chemistry because their amphoteric character, hydrogen-bonding capability, and electron-rich aromatic system can support diverse biological activities and favorable pharmacological interactions [77]. In the present scaffold series, these features may have contributed to the favorable potency–selectivity balance observed for **3b**.

Compound **3c**, containing a thiazole ring, also showed sustained time-dependent cytotoxicity together with moderate selectivity toward A431 cells. Thiazole-containing heterocycles are frequently incorporated into anticancer-oriented hybrid molecules because the sulfur–nitrogen heteroaromatic system can influence lipophilicity, electronic distribution, and intermolecular binding behavior. Related pyrazoline-thiazole derivatives have been reported to exhibit growth-inhibitory activity and VEGFR-2 inhibitory potential, supporting the biological relevance of this heterocyclic combination in anticancer scaffold design [78]. The sustained activity observed for **3c** during extended exposure may therefore reflect beneficial electronic and physicochemical contributions associated with the thiazole substituent.

Compound **3e**, incorporating a benzimidazole moiety, also demonstrated strong growth-inhibitory behavior, particularly at 48 h exposure. Benzimidazole-containing derivatives are well established in medicinal chemistry and have frequently been associated with anticancer activity because their fused bicyclic aromatic framework can support favorable target interactions and biologically relevant binding behavior [79,80]. In the present series, benzimidazole incorporation may have contributed to the pronounced short-term suppression of A431 viability observed for **3e**. However, the marked reduction in selectivity at 72 h suggests that increased potency was accompanied by delayed toxicity toward HaCaT cells, indicating that benzimidazole fusion alone was insufficient to maintain sustained cancer-cell selectivity in this structural context.

Collectively, these findings indicate that substituent variation within the heteroaromatic moiety modulates biological response in this scaffold series. Differences in potency and selectivity across compounds **3a**–**3f** are consistent with substituent-dependent effects on physicochemical and electronic properties, although these parameters were not directly quantified. Similar structure-dependent growth-inhibitory trends have been reported for hydrazone- and pyrazole-containing systems, where heteroaromatic substitution, lipophilicity, hydrogen-bonding capacity, and electronic distribution contribute substantially to biological response behavior [8].

Interestingly, compounds with stronger cytotoxic responses did not show corresponding migration-inhibitory activity in scratch assays, suggesting that growth-inhibitory potency and migration-related phenotypes may be regulated independently. These findings emphasize the context-dependent biological behavior of the synthesized derivatives and support further mechanistic investigation.

##### IC_50_ and Selectivity Analysis

To quantitatively evaluate cytotoxic potency and cancer-cell selectivity, IC_50_ values were estimated from MTT dose–response profiles for A431 and HaCaT cells following 24, 48, and 72 h exposure to compounds **3a**–**3f**. Selectivity indices were calculated as *SI* = IC_50_(HaCaT)/IC_50_(A431), where *SI* > 1 indicates preferential cytotoxicity toward A431 cells. Together, these parameters provide a comparative framework for distinguishing compounds with favorable in vitro potency–selectivity profiles from analogs showing broader nonspecific cytotoxicity (Table 4).

IC_50_ values were estimated from MTT viability data using log-dose interpolation of concentration–response profiles. When IC_50_ values exceeded the highest tested concentration, they were reported as >1 mM, and *SI* values were presented as minimum estimates where applicable. ND indicates not determinable. Because IC_50_ values are strongly influenced by cell line, assay type, exposure duration, and compound solubility, these values were interpreted comparatively within the present compound series rather than as direct potency equivalents to previously reported heteroaromatic anticancer scaffolds.

Overall, IC_50_ values generally decreased with increasing exposure time, indicating enhanced cytotoxicity during prolonged treatment. Compounds **3b** and **3c** showed the most consistent balance between cytotoxic potency and preferential A431 suppression across multiple exposure periods. Compound **3b**, bearing an imidazole substituent, showed improved selectivity at 48 h and retained preferential A431 activity at 72 h. This profile is consistent with the broad medicinal relevance of imidazole-containing scaffolds, whose amphoteric character, hydrogen-bonding capacity, and electron-rich heteroaromatic system can support diverse biological interactions and pharmacological activity [77].

Compound **3c**, containing a thiazole ring, also displayed a favorable time-dependent profile, with measurable selectivity toward A431 cells at both 48 and 72 h. Thiazole-containing hybrid molecules are frequently explored in anticancer scaffold design because the sulfur–nitrogen heteroaromatic system can influence electronic distribution, lipophilicity, and target-interaction potential. Related pyrazoline-thiazole derivatives have been reported to show anticancer activity and enzyme-inhibitory potential, supporting the relevance of this heterocyclic motif in growth-inhibitory compound design [78].

Compound **3e** showed the highest apparent selectivity at 48 h, with an *SI* of approximately 36.47, suggesting strong preferential suppression of A431 cells under these conditions. However, this effect was not maintained at 72 h, when increased HaCaT sensitivity reduced the *SI* to 0.17. Benzimidazole derivatives are well established as bioactive medicinal scaffolds and have been widely investigated for anticancer activity, including 2-substituted benzimidazole derivatives with cytotoxic potential [79,80]. In the present series, the benzimidazole moiety may have contributed to the strong short-term activity of **3e**, but the delayed HaCaT toxicity indicates that this substituent did not provide sustained cancer-cell selectivity.

In contrast, compounds **3d** and **3f** showed strong cytotoxicity but weak selectivity because HaCaT IC_50_ values were equal to or lower than those observed in A431 cells at several time points. These findings suggest that high cytotoxic potency alone is insufficient to define a favorable anticancer profile and that selectivity must be interpreted alongside time-dependent toxicity toward non-malignant keratinocytes. Compound **3a** displayed moderate but more stable selectivity, particularly at 48 h, although its overall potency was lower than that of **3b**, **3c**, and **3e**.

Taken together, the IC_50_ and *SI* analyses indicate that imidazole-bearing **3b** and thiazole-bearing **3c** represent the most balanced analogs in terms of sustained potency and preferential A431 activity, whereas benzimidazole-bearing **3e** represents a highly active but temporally unstable analog. These findings are consistent with broader SAR observations reported for hydrazone- and pyrazole-containing systems, where heteroaromatic substitution, hydrogen-bonding capacity, electronic distribution, and lipophilicity can strongly influence growth-inhibitory and antioxidant behavior [8].

##### Mechanistic Considerations

Although the molecular mechanisms underlying cytotoxicity were not directly investigated, the concentration- and time-dependent response patterns observed in the present study suggest a multiphasic biological response involving early metabolic adaptation followed by progressive cytotoxicity. In several treatment groups, especially at 0.001–0.01 mM, viability approached or slightly exceeded control levels, most notably in A431 cells during prolonged exposure. Because MTT reduction primarily reflects mitochondrial and cytosolic NAD(P)H-dependent metabolic activity rather than direct cell number, these transient increases may reflect compensatory metabolic activation or altered tetrazolium reduction efficiency rather than true proliferative enhancement [75,76].

Similar biphasic or hormetic-like responses have been reported in tetrazolium-based viability assays and are commonly associated with adaptive stress signaling, transient metabolic compensation, and altered mitochondrial redox activity [81,82]. In cancer cells, low-level chemical stress may initially stimulate survival-associated metabolic pathways before prolonged or high-intensity exposure shifts the cellular response toward irreversible injury. Such adaptive behavior is also broadly consistent with the dynamic stress-response and metabolic plasticity characteristics now recognized as important features of malignant cells [83].

At higher concentrations and longer exposure periods, all compounds reduced MTT signal intensity, consistent with impaired metabolic viability and progressive cellular stress. The pronounced decline observed at 0.1–1 mM, particularly for compounds **3b**, **3c**, and **3e**, suggests that prolonged exposure exceeded compensatory capacity and shifted cells toward pronounced cytotoxic response. These patterns are consistent with cellular stress responses that may involve mitochondrial dysfunction, oxidative imbalance, and stress-associated apoptotic signaling. Sustained viability reductions during 48–72 h exposure further support delayed stress-mediated cytotoxicity rather than exclusively acute injury.

Differences in selectivity between A431 and HaCaT cells may also reflect distinct metabolic or stress-response characteristics. The greater sensitivity of A431 cells to several compounds may be associated with altered oxidative metabolism, increased redox vulnerability, or reduced tolerance to treatment-induced cellular stress, all of which are commonly associated with malignant transformation and tumor-associated metabolic reprogramming [83]. However, the molecular basis of these differential responses was not directly evaluated in the present study.

Collectively, compounds **3a**–**3f** support a model in which low-dose exposure may initially induce adaptive metabolic responses, whereas prolonged or high-dose exposure shifts cells toward pronounced cytotoxic response. Future studies incorporating intracellular ROS measurements, mitochondrial membrane potential analysis, Hoechst 33,342/propidium iodide staining, caspase-3/7 activation assays, BAX/BCL-2 expression analysis, and p38/MAPK signaling evaluation will be necessary to clarify the underlying mechanisms and distinguish cytostatic from cytotoxic effects.

#### 2.3.8. Scratch Assay-Based Wound Closure Analysis

The effects of compounds **3a**–**3f** on wound closure were evaluated using a scratch, or wound-healing, assay in A431 cells and HaCaT keratinocytes. This assay is widely used to assess collective wound closure dynamics, which may reflect contributions from migration, adhesion, survival, and residual proliferative effects [84,85]. Wound closure was quantified at 14 h and 18 h relative to the initial wound area at 0 h for each replicate. Data are expressed as mean ± SD from three independent biological replicates.

##### Overall Trends in Wound Closure

**Figure 12:**
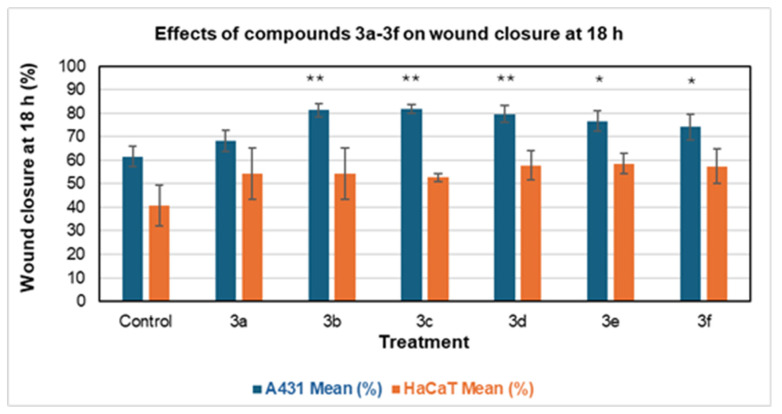
Effect of compounds **3a**–**3f** on wound closure in A431 and HaCaT cells at 18 h. Wound closure (%) was calculated relative to the initial wound area at 0 h and is presented as mean ± SD from three independent biological replicates (*n* = 3). Experiments were performed under serum-free conditions in the presence of mitomycin C to minimize proliferative contributions; therefore, wound closure responses are interpreted primarily as migration-associated wound closure dynamics rather than direct proliferative effects. Statistical significance was determined using Welch’s *t*-test to compare each treated group with the corresponding control within each cell line. *p* < 0.05 (*) and *p* < 0.01 (**) indicate significant differences versus control.

Across the compound series, no consistent inhibition of wound closure was observed at the tested concentration of 10 µM. Instead, several compounds increased wound closure relative to the corresponding control, particularly in A431 cells at 18 h (Figure 12). These findings indicate that, under the present experimental conditions, compounds **3a**–**3f** did not demonstrate anti-migratory activity. Rather, the observed increases in wound closure suggest context-dependent effects on migration-associated wound closure dynamics. Complete time-dependent wound closure profiles for all compounds in A431 and HaCaT cells are provided in the Supplementary Materials (Figures S42–S47). The increased wound closure observed in A431 cells contrasts with the cytotoxic effects observed at higher concentrations in MTT assays, suggesting concentration- and context-dependent biological responses.

##### Compound-Specific Wound Closure Behavior

**Figure 13:**
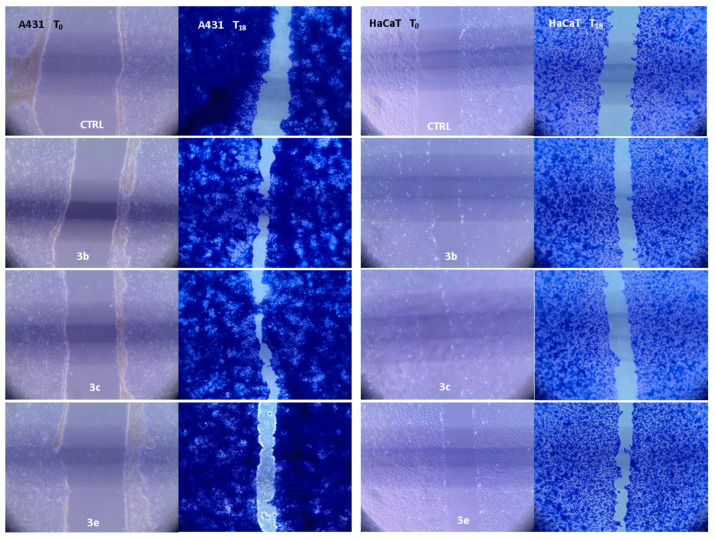
Representative wound-healing images of A431 and HaCaT cells treated with compounds **3b**, **3c**, and **3e** (10 µM) under serum-free conditions in the presence of mitomycin C. Images were acquired immediately after scratch generation (0 h) and after 18 h incubation. Increased wound closure was observed in treated A431 cells relative to the corresponding control groups, whereas more modest differences were observed in HaCaT cells.

Among the evaluated derivatives, compounds **3b**, **3c**, and **3e** displayed the most notable wound closure responses in A431 cells and were therefore selected for comparative interpretation (Figure 13). Interestingly, these compounds also represented the most biologically active analogs in the MTT cytotoxicity assays, indicating that growth-inhibitory potency and wound closure behavior did not show a direct linear relationship under the present experimental conditions.

Compound **3b** did not inhibit wound closure in A431 cells. No significant difference was observed at 14 h (*p* = 0.571); however, wound closure increased significantly at 18 h (81.2 ± 2.8%, *p* = 0.0046) relative to the corresponding control (61.5 ± 4.3%). In HaCaT cells, closure values were consistently higher than control but did not reach statistical significance. These findings indicate enhanced late-stage wound closure dynamics in A431 cells.

Compound **3c** produced the strongest and most sustained increase in A431 wound closure among the tested compounds. Closure increased significantly at both 14 h (69.4 ± 4.0%, *p* = 0.0045) and 18 h (81.6 ± 1.9%, *p* = 0.0070) compared with control values. In HaCaT cells, wound closure also exceeded control levels but did not reach significance. These findings suggest that the thiazole-containing analog **3c** was associated with enhanced wound closure behavior under the tested conditions.

Compound **3e** also increased wound closure in A431 cells at both 14 h (60.8 ± 3.7%, *p* = 0.033) and 18 h (76.6 ± 4.4%, *p* = 0.013). In HaCaT cells, closure values were elevated relative to control and approached statistical significance at 18 h (*p* = 0.053). Notably, compound **3e** also exhibited the strongest short-term selectivity in MTT assays at 48 h, indicating that pronounced cytotoxic selectivity and enhanced wound closure behavior may coexist under different exposure conditions.

The remaining compounds (**3a**, **3d**, and **3f**) demonstrated variable effects on wound closure and are presented in the Supplementary Materials (Figures S42–S47). Collectively, these findings suggest that wound closure responses at low non-lethal concentrations may reflect adaptive or context-dependent cellular behavior distinct from the cytotoxic effects observed at higher concentrations in MTT assays.

##### Comparative Interpretation of Wound Closure Behavior

Across the compound series, none of the derivatives produced sustained inhibition of wound closure under the tested conditions. Instead, several compounds, particularly **3c**, **3d**, **3e**, and **3f**, significantly increased wound closure in A431 cells, especially at 18 h. In HaCaT cells, similar trends toward increased closure were observed but generally did not reach statistical significance.

Although serum-free conditions and mitomycin C were used to minimize proliferative contributions, minor effects arising from proliferation, stress-associated or adaptive signaling responses, or altered cellular stress responses cannot be completely excluded, as wound-healing assays may reflect combined influences of migration, proliferation, adhesion dynamics, and cell survival [86,87]. Consequently, increased wound closure should be interpreted cautiously as migration-associated wound closure behavior rather than as definitive evidence of enhanced migratory capacity alone.

Given the MTT cytotoxicity profiles, it is also possible that sub-cytotoxic exposure conditions triggered adaptive or compensatory cellular responses that influenced wound closure dynamics. This interpretation is supported by the fact that the scratch assay was performed at 10 µM, whereas the MTT assay evaluated a broader concentration range extending up to 1 mM; therefore, the wound closure and cytotoxicity assays reflect different concentration-dependent biological response windows. Similar low-dose adaptive responses have been described in hormesis and cellular stress adaptation models, where mild chemical stress transiently enhances survival-associated or metabolically adaptive cellular behavior before higher-dose exposure induces a pronounced cytotoxic response [83,84]. In malignant cells, such responses may additionally reflect stress-associated metabolic plasticity and altered signaling behavior, features now recognized as important components of cancer-cell adaptation and phenotypic flexibility [83].

Interestingly, compounds **3b**, **3c**, and **3e** demonstrated the most favorable growth-inhibitory profiles in MTT assays while simultaneously failing to suppress wound closure under sub-cytotoxic migration conditions. Divergence between cytotoxic and migration-associated cellular responses has been reported for several anticancer scaffolds, indicating that growth-inhibitory potency does not necessarily predict suppression of wound closure dynamics under all exposure conditions [88]. These findings emphasize the context-dependent biological behavior of the present carbohydrazone series and further suggest that migration-associated responses and cytotoxic signaling may involve partially distinct cellular response pathways.

Collectively, these findings indicate context-dependent biological behavior in which cytotoxicity and wound closure responses are not directly coupled under all exposure conditions. Nevertheless, because the present study did not include assays specifically designed to distinguish migration from proliferation-related wound closure effects, the mechanistic basis of the observed responses remains speculative. Future studies incorporating transwell migration assays, invasion assays, cytoskeletal staining, EMT-marker analysis, and real-time live-cell imaging would help clarify the relationship between cytotoxicity, migration-associated behavior, and adaptive stress signaling in this scaffold series [85,86,87].

## 3. Materials and Methods

### 3.1. Materials and Physical Measurements

All reagents and solvents were purchased from commercial suppliers, including Merck (Darmstadt, Germany), Sigma-Aldrich/Merck, and Fisher Scientific (Waltham, MA, USA), and used without further purification unless otherwise specified. Compound **1** (1-(2-methoxyphenyl)-5-methyl-1*H*-pyrazole-4-carbohydrazide) was obtained commercially from Sigma-Aldrich/Merck (Cat. No. L317810) and used without further purification. Melting points were determined in open glass capillaries using an Electrothermal IA9200 melting point apparatus (Staffordshire, UK) and are uncorrected. ATR-FTIR spectra were recorded on a Shimadzu Affinity-1 spectrophotometer (Duisburg, Germany) using the attenuated total reflectance (ATR) method in the range of 4000–400 cm^−1^. ^1^H NMR spectra were recorded on a Varian Mercury-400 spectrometer (Palo Alto, CA, USA) at 400 MHz, and APT-^13^C NMR spectra were recorded on the same instrument at 100 MHz using DMSO-d_6_ as solvent. Chemical shifts (δ) are reported in parts per million (ppm), and coupling constants (J) are given in Hertz (Hz). Signal multiplicities are designated as follows: s, singlet; d, doublet; t, triplet; m, multiplet; and br, broad. High-resolution mass spectrometry (HRMS) analyses were performed on an Agilent 6530B LC-QTOF mass spectrometer (Santa Clara, CA, USA) operating in positive electrospray ionization mode (ESI+). Data acquisition was carried out using the MassHunter Acquisition Software (6200 series TOF/6500 series Q-TOF, version 11.0.203.0). Instrument calibration status was verified prior to analysis (IRM calibration status: Success), and the tune mass range was set to 3200 *m*/*z*. Fragmentor voltage and collision energy values obtained from the analytical reports were 90 V and 0 eV, respectively. Mass measurements were evaluated with an accuracy of ±5 ppm. Reaction progress was monitored by thin-layer chromatography (TLC) on silica gel 60 F254 aluminum plates (Merck) using ethyl acetate/petroleum ether (3:2, *v*/*v*) as the mobile phase, and spots were visualized under UV light at 254 nm. UV-Vis spectra for CT-DNA-associated spectroscopic studies were recorded on a Shimadzu UV-1800 spectrophotometer (Kyoto, Japan), and fluorescence measurements were carried out on a Shimadzu RF-6000 spectrofluorophotometer (Kyoto, Japan). For antioxidant activity assays and SPF determination, absorbance values were recorded using a BioTek Epoch 2 microplate reader (Winooski, VT, USA). A431 (human epidermoid carcinoma) and HaCaT (immortalized human keratinocyte) cell lines were originally obtained from Furman University (Greenville, SC, USA) and maintained in our laboratory. Cells were used at low passage numbers throughout the experiments.

### 3.2. General Protocol for the Synthesis of Pyrazole-Based Carbohydrazone Derivatives (**3a**–**3f**)

The target compounds (**3a**–**3f**) were synthesized via condensation of carbohydrazide 1 with the corresponding aldehydes (**2a**–**2f**). In a typical procedure, the appropriate aldehyde (0.305 mmol) was dissolved in absolute ethanol (4 mL), and an equimolar amount of carbohydrazide **1** (0.305 mmol) was added slowly under stirring. After a homogeneous mixture was obtained, glacial acetic acid (3 drops) was added as a catalyst. The reaction mixture was refluxed for 3–5 h, and the progress of the reaction was monitored by TLC. Upon completion of the reaction, as indicated by the disappearance of the starting materials, the mixture was allowed to cool to room temperature, and the precipitated solid was collected by filtration. The crude products were washed successively with diethyl ether and cold ethanol, dried, and further purified by repeated recrystallization from acetonitrile. The purification process was continued until a single spot was observed on TLC, and the identity and purity of the isolated compounds were further supported by spectroscopic analyses. The synthetic route for compounds **3a**–**3f** is shown in Scheme 1.

1-(2-Methoxyphenyl)-5-methyl-N′-[(1-methyl-1H-pyrrol-2-yl)methylene]-1H-pyrazole-4-carbohydrazide (**3a**)

**Yield**: 50% (51.40 mg); **Time**: 5 h; **mp**: 247–248 °C (decomp.); **R*f*** = 0.35 (ethyl acetate/petroleum ether, 3:2). **FTIR (ATR, ν, cm^−1^)**: 3220 (br, N–H), 3100–3000 (aromatic/heteroaromatic C–H), 2947, 2839 (aliphatic C–H), 1643 (C=O), 1597 (C=N), 1566–1419 (aromatic/heteroaromatic skeletal vibrations), 1257 (C–O), 1111, 1047 (C–O), 810, 740, 648 (aromatic C–H out-of-plane bending). **^1^H NMR (400 MHz, DMSO-*d_6_*, δ ppm)**: 11.20 (s, 1H, NH), 8.31 (s, 1H, CH=N), 8.12 (s, 1H, pyrazole-H), 7.54 (t, *J* = 7.4 Hz, 1H, Ar–H), 7.35 (m, 1H, Ar–H), 7.27 (d, *J* = 8.4 Hz, 1H, Ar–H), 7.12 (t, *J* = 7.6 Hz, 1H, Ar–H), 6.96 (d, *J* = 4.0 Hz, 1H, pyrrole-H), 6.49 (d, *J* = 4.0 Hz, 1H, pyrrole-H), 6.11 (t, *J* = 4.1 Hz, 1H, pyrrole-H), 3.87 (s, 3H, OCH_3_), 3.79 (s, 3H, NCH_3_), 2.28 (s, 3H, CH_3_). **^13^C NMR (100 MHz, DMSO-*d_6_*, δ ppm)**: 159.45 (C=O), 154.53 (Ar–O ipso-C), 144.28, 139.99, 138.91, 131.45, 129.26, 128.15, 127.69, 127.51, 121.21, 114.42, 113.66, 112.62, 108.74, 53.63 (OCH_3_), 36.28 (NCH_3_), 11.10 (CH_3_). **HRMS (ESI)**: calcd for C_18_H_20_N_5_O_2_^+^ [M+H]^+^ 338.16115; found 338.16151.

1-(2-Methoxyphenyl)-5-methyl-N′-[(1-methyl-1H-imidazol-2-yl)methylene]-1H-pyrazole-4-carbohydrazide (**3b**)

**Yield**: 40% (34.22 mg); **Time**: 5 h; **mp**: 199–201 °C; **R*f*** = 0.10 (ethyl acetate/petroleum ether, 3:2). **FTIR (ATR, ν, cm^−1^)**: 3130 (N–H), 3100–3000 (aromatic/heteroaromatic C–H), 2947, 2839 (aliphatic C–H), 1674 (C=O), ca. 1600 (C=N), 1558–1411 (aromatic/heteroaromatic skeletal vibrations), 1262 (C–O), 1134, 1018 (C–O), 763, 655 (aromatic C–H out-of-plane bending). **^1^H NMR (400 MHz, DMSO-*d_6_*, δ ppm)**: 14.47 (s, 1H, NH), 8.02 (s, 1H, CH=N), 7.68 (s, 1H, pyrazole-H), 7.55 (t, *J* = 7.9 Hz, 1H, Ar–H), 7.42–7.36 (m, 3H, Ar–H and heteroaromatic H), 7.28 (d, *J* = 8.3 Hz, 1H, Ar–H), 7.13 (t, *J* = 7.6 Hz, 1H, Ar–H), 3.80 (s, 3H, OCH_3_), 3.78 (s, 3H, NCH_3_), 2.33 (s, 3H, CH_3_). **^13^C NMR (100 MHz, DMSO-*d_6_*, δ ppm)**: 159.86 (C=O), 154.54 (Ar–O ipso-C), 144.62, 141.37, 138.64, 131.61, 129.26, 128.52, 127.35, 127.09, 123.87, 121.76, 121.25, 113.11, 56.25 (OCH_3_), 33.50 (NCH_3_), 11.23 (CH_3_). **HRMS (ESI)**: calcd for C_17_H_19_N_6_O_2_^+^ [M+H]^+^ 339.15640; found 339.15642.

1-(2-Methoxyphenyl)-5-methyl-N′-(thiazol-2-ylmethylene)-1H-pyrazole-4-carbohydrazide (**3c**)

**Yield**: 65% (36.27 mg); **Time**: 4 h; **mp**: 192–193 °C; **R*f*** = 0.33 (ethyl acetate/petroleum ether, 3:2). **FTIR (ATR, ν, cm^−1^)**: 3220 (br, N–H), 3100–3000 (aromatic/heteroaromatic C–H), 2970, 2885, 2839 (aliphatic C–H), 1643 (C=O), 1576–1481 (aromatic/heteroaromatic skeletal vibrations, including contributions from C=N stretching vibrations), 1280 (C–O), 1126, 1018 (C–O), 887, 743, 694 (aromatic C–H out-of-plane bending). **^1^H NMR (400 MHz, DMSO-*d_6_*, δ ppm)**: 11.83 (s, 1H, NH), 8.62 (s, 1H, CH=N), 8.18 (s, 1H, pyrazole-H), 7.96 (d, *J* = 3.0 Hz, 1H, thiazole-H), 7.84 (d, *J* = 3.0 Hz, 1H, thiazole-H), 7.55 (t, *J* = 8.1 Hz, 1H, Ar–H), 7.37 (m, 1H, Ar–H), 7.28 (d, *J* = 8.6 Hz, 1H, Ar–H), 7.13 (t, *J* = 7.5 Hz, 1H, Ar–H), 3.79 (s, 3H, OCH_3_), 2.30 (s, 3H, CH_3_). **^13^C NMR (100 MHz, DMSO-*d_6_*, δ ppm)**: 164.94 (C=O), 160.46, 154.51 (Ar–O ipso-C), 144.47, 143.75, 138.85, 136.54, 131.57, 127.38, 122.24, 122.16, 121.25, 119.65, 113.06, 56.77 (OCH_3_), 10.72 (CH_3_). **HRMS (ESI)**: calcd for C_16_H_16_N_5_O_2_S^+^ [M+H]^+^ 342.10192; found 342.10199.

1-(2-Methoxyphenyl)-5-methyl-N′-[(1-methyl-1H-indol-3-yl)methylene]-1H-pyrazole-4-carbohydrazide (**3d**)

**Yield**: 70% (49.47 mg); **Time**: 3 h; **mp**: 250–251 °C (decomp.); **R*f*** = 0.20 (ethyl acetate/petroleum ether, 3:2). **FTIR (ATR, ν, cm^−1^)**: 3180 (br, N–H), 3100–3000 (aromatic/heteroaromatic C–H), 2939, 2839 (aliphatic C–H), 1627 (C=O), 1604 (C=N), 1566–1465 (aromatic/heteroaromatic skeletal vibrations), 1280 (C–O), 1118, 1018 (C–O), 879, 740 (aromatic C–H out-of-plane bending). **^1^H NMR (400 MHz, DMSO-*d_6_*, δ ppm)**: 11.19 (s, 1H, NH), 8.53 (s, 1H, CH=N), 8.31 (d, *J* = 7.9 Hz, 1H, Ar–H), 8.16 (s, 1H, pyrazole-H), 7.80 (s, 1H, indole-H), 7.57–7.50 (m, 3H, Ar–H), 7.37–7.26 (m, 2H, Ar–H), 7.22–7.11 (m, 2H, Ar–H), 3.83 (s, 3H, OCH_3_), 3.80 (s, 3H, NCH_3_), 2.32 (s, 3H, CH_3_). **^13^C NMR (100 MHz, DMSO-*d_6_*, δ ppm)**: 159.39 (C=O), 154.57 (Ar–O ipso-C), 144.15, 143.41, 138.98, 138.01, 133.99, 131.43, 129.30, 127.59, 125.21, 123.13, 122.56, 121.22, 121.02, 113.92, 113.08, 111.33, 110.62, 54.17 (OCH_3_), 29.51 (NCH_3_), 11.12 (CH_3_). **HRMS (ESI)**: calcd for C_22_H_22_N_5_O_2_^+^ [M+H]^+^ 388.17680; found 388.17675.

1-(2-Methoxyphenyl)-5-methyl-N′-[(1-methyl-1H-benzo[d]imidazol-2-yl)methylene]-1H-pyrazole-4-carbohydrazide (**3e**)

**Yield**: 62% (47.78 mg); **Time**: 4 h; **mp**: 228–230 °C; **R*f*** = 0.23 (ethyl acetate/petroleum ether, 3:2). **FTIR (ATR, ν, cm^−1^)**: 3260–3200 (br, N–H), 3100–3000 (aromatic/heteroaromatic C–H), 2924, 2846 (aliphatic C–H), 1651 (C=O), 1604 (C=N), 1558–1404 (aromatic/heteroaromatic skeletal vibrations), 1265 (C–O), 1134, 1018 (C–O), 732, 632 (aromatic C–H out-of-plane bending). **^1^H NMR (400 MHz, DMSO-*d_6_*, δ ppm)**: 11.86 (s, 1H, NH), 8.54 (s, 1H, CH=N), 8.22 (s, 1H, pyrazole-H), 7.99 (d, *J* = 7.8 Hz, 1H, Ar–H), 7.85 (d, *J* = 7.8 Hz, 1H, Ar–H), 7.77 (m, 1H, Ar–H), 7.58–7.47 (m, 3H, Ar–H and heteroaromatic H), 7.30 (t, *J* = 7.5 Hz, 1H, Ar–H), 7.08 (m, 1H, heteroaromatic H), 4.07 (s, 3H, NCH_3_), 3.81 (s, 3H, OCH_3_), 2.33 (s, 3H, CH_3_). **^13^C NMR (100 MHz, DMSO-*d_6_*, δ ppm)**: 163.60 (C=O), 154.51 (Ar–O ipso-C), 147.40, 142.96, 141.25, 134.25, 131.67, 131.58, 129.27, 127.37, 124.18, 124.02, 122.85, 121.25, 120.92, 119.95, 113.10, 111.01, 56.27 (OCH_3_), 30.72 (NCH_3_), 11.26 (CH_3_). **HRMS (ESI)**: calcd for C_21_H_21_N_6_O_2_^+^ [M+H]^+^ 389.17205; found 389.17230.

N′-(Benzo[d]thiazol-2-ylmethylene)-1-(2-methoxyphenyl)-5-methyl-1H-pyrazole-4-carbohydrazide (**3f**)

**Yield**: 80% (51.24 mg); **Time**: 4 h; **mp**: 217–218 °C; **R*f*** = 0.43 (ethyl acetate/petroleum ether, 3:2). **FTIR (ATR, ν, cm^−1^)**: 3270 (br, N–H), 3100–3000 (aromatic/heteroaromatic C–H), 2924, 2846 (aliphatic C–H), 1643 (C=O), 1597 (C=N), 1518–1458 (aromatic/heteroaromatic skeletal vibrations), 1265 (C–O), 1172, 1026 (C–O), 871, 748, 694 (aromatic C–H out-of-plane bending). **^1^H NMR (400 MHz, DMSO-*d_6_*, δ ppm)**: 12.06 (s, 1H, NH), 8.71 (s, 1H, CH=N), 8.16 (d, *J* = 7.7 Hz, 1H, Ar–H), 8.05 (d, *J* = 7.9 Hz, 1H, Ar–H), 7.58–7.50 (m, 3H, Ar–H), 7.39 (d, *J* = 7.5 Hz, 1H, Ar–H), 7.29 (d, *J* = 8.4 Hz, 1H, Ar–H), 7.14 (t, *J* = 7.8 Hz, 1H, Ar–H), 3.81 (s, 3H, OCH_3_), 2.32 (s, 3H, CH_3_). **^13^C NMR (100 MHz, DMSO-*d_6_*, δ ppm)**: 165.76 (C=O), 160.19, 154.51 (Ar–O ipso-C), 153.63, 143.57, 141.23, 139.09, 134.38, 131.63, 129.28, 127.33, 127.14, 126.91, 122.94, 122.65, 121.29, 120.06, 113.11, 56.27 (OCH_3_), 11.35 (CH_3_). **HRMS (ESI)**: calcd for C_20_H_18_N_5_O_2_S^+^ [M+H]^+^ 392.11757; found 392.11735.

### 3.3. Biological Assays

All biological assays were performed using mass-based concentrations (µg/mL). The corresponding molar concentrations (µM or mM), calculated from the molecular weight of each derivative, are provided in Table S1.

#### 3.3.1. DPPH Radical Scavenging Activity

The DPPH radical scavenging activity of the compounds was evaluated according to the method of Blois with minor modifications [89]. Briefly, 125 μL of each test solution at five different concentrations (0.50–10.00 μg/mL) was mixed with 75 μL of a 0.25 mM DPPH solution in ethanol. The mixtures were incubated for 60 min at room temperature in the dark, and the absorbance was then recorded at 517 nm using a microplate reader (Epoch 2, BioTek, Winooski, VT, USA). BHT (butylated hydroxytoluene) and BHA (butylated hydroxyanisole) were used as positive controls, while ethanol without DPPH was used as the blank. The percentage inhibition of DPPH radicals was calculated using Equation (1):

Inhibition (%) = [(*A*_control_ − *A*_sample_)/*A*_control_] × 100 (1)

#### 3.3.2. Ferrous Ion (Fe^2+^) Chelating Activity

The Fe^2+^-chelating activity of the compounds was determined using the procedure described by Decker and Welch, with minor modifications [90]. For each assay, 0.25 mL of the compound solution (0.50–10.00 μg/mL) was combined with 0.925 mL of deionized water and 0.025 mL of FeCl_2_ (2.0 mM), and the mixture was allowed to stand at room temperature for 30 min. Subsequently, 0.05 mL of ferrozine (5.0 mM) was added, the solution was mixed thoroughly, and incubation was continued for an additional 25 min at room temperature. The blank consisted of FeCl_2_ and deionized water without ferrozine to correct for background absorbance unrelated to Fe^2+^-ferrozine complex formation. EDTA (ethylenediaminetetraacetic acid) and BHT were employed as reference standards, while the control contained FeCl_2_, ferrozine, and deionized water. Absorbance was recorded at 562 nm, and the percentage inhibition of Fe^2+^ ions was calculated according to Equation (1).

#### 3.3.3. Total Antioxidant Capacity (TAC)

The inhibition of lipid peroxidation was evaluated using the ferric thiocyanate (FTC) method described by Mitsuda et al. and later adapted in antioxidant studies [91,92]. For each assay, appropriate volumes of the test solutions at the desired concentrations were mixed with 0.04 M phosphate buffer (pH 7.4) and a Tween 20-linoleic acid emulsion prepared in the same buffer, to give a final reaction volume of 1.0 mL. The mixtures were incubated at 37 °C. At 10 h intervals, 10 μL aliquots were withdrawn from the reaction mixtures and mixed with 470 μL ethanol, followed by addition of FeCl_2_ (20 mM) and ammonium thiocyanate (30%, *w*/*v*). After 5 min at room temperature, absorbance was measured at 500 nm. This procedure was repeated at 10 h intervals until the control sample reached its maximum absorbance. Two additional measurements were recorded after the absorbance of the control began to decline, and the experiment was then terminated. α-Tocopherol and ascorbic acid were used as standards, while an ethanol-FeCl_2_-ammonium thiocyanate mixture served as the blank. The percentage inhibition of lipid peroxidation was calculated using Equation (1).

#### 3.3.4. In Vitro Sun Protection Factor (SPF)

The photoprotective activity of the synthesized compounds was determined using the UV spectrophotometric method described by Mansur et al. [93]. Solutions of the test compounds at different concentrations (0.50–10.00 µg/mL) were prepared in an ethanol:water mixture (3:2, *v*/*v*). The absorbance of each solution was recorded between 290 and 320 nm, at 5 nm intervals, using ethanol:water (3:2, *v*/*v*) as the solvent blank. The in vitro sun protection factor (SPF) was calculated according to the Mansur Equation (2):

(2) $$SPF\;value=CF\sum_{290}^{320}EE\left(\lambda \right)\;\times \;I\left(\lambda \right)\;\times \;A(\lambda )$$

In this equation, A(λ) is the measured absorbance at wavelength λ, I (λ) is the normalized solar intensity spectrum, EE (λ) is the erythemal effect spectrum, and CF is the correction factor (CF = 10). The constant EE × I (λ) values for each wavelength were taken from the data reported by Sayre et al. [94]. Natural carrot seed oil was used as the reference standard.

#### 3.3.5. CT-DNA-Associated Spectroscopic Studies

##### UV-Vis Absorption Studies

CT-DNA-associated spectroscopic studies were performed using UV-Vis absorption spectroscopy. All experiments were carried out in 10 mM Tris-HCl buffer containing 50 mM NaCl (pH 7.4). Calf thymus DNA (CT-DNA) was dissolved in the same buffer and allowed to equilibrate at 4 °C for 24 h. The purity of DNA was verified by the A_260_/A_280_ ratio (1.87), indicating minimal protein contamination. DNA concentrations were determined spectrophotometrically using an extinction coefficient of 6600 M^−1^ cm^−1^ and are reported as nucleotide (base) concentrations [70]. Stock solutions of the compounds (1 × 10^−3^ M) were prepared in DMSO and diluted with buffer to the desired working concentrations. The final DMSO concentration in all experiments was maintained below 1% (*v*/*v*). Absorption spectra were recorded in quartz cuvettes while increasing concentrations of CT-DNA were added to compound solutions. After each addition, samples were incubated at 37 °C for 30 min before measurement. Changes in spectral profiles were monitored qualitatively to evaluate CT-DNA-associated absorption responses.

##### Fluorescence Spectroscopic Studies

Fluorescence spectroscopy was employed as a complementary method to evaluate CT-DNA-associated emission responses. The excitation wavelength for each compound was selected individually according to its absorption characteristics (248–330 nm). Emission spectra were recorded under the same buffer conditions used for the UV-Vis experiments. All solutions were prepared in 10 mM Tris-HCl buffer containing 50 mM NaCl at pH 7.4. Stock solutions of the compounds were prepared in DMSO, and the final DMSO concentration in all experiments was maintained below 1% (*v*/*v*). Fluorescence measurements were performed by adding increasing concentrations of CT-DNA (0–3 µM) to solutions containing fixed concentrations of the compounds (25–45 µM). After each addition, the solutions were mixed gently and incubated at 37 °C for 30 min prior to measurement. Fluorescence responses were evaluated by comparing emission intensity changes relative to the corresponding DNA-free control. These measurements were used for qualitative comparison of CT-DNA-associated fluorescence behavior rather than for quantitative determination of binding constants.

#### 3.3.6. Cell Cultures

Human keratinocyte cell lines A431 human epidermoid carcinoma cells and HaCaT immortalized human keratinocytes were maintained as monolayer cultures in Dulbecco’s Modified Eagle Medium (DMEM) supplemented with 10% fetal bovine serum (FBS). To prevent contamination, gentamicin (1%) and amphotericin B (2 µg/mL) were added to the culture medium. Cells were incubated at 37 °C in a humidified cell culture incubator with 5% CO_2_. Subculturing was performed every third day at a 1:10 ratio. After aspirating the culture medium, cells were rinsed once with PBS and then incubated with Trypsin-EDTA for 5 min at 37 °C to facilitate detachment. Detached cells were collected into 15 mL conical tubes, centrifuged at 200 *g* for 2 min, and resuspended in warm complete medium. Total cell counts and viability were assessed using the Countess^®^ Automated Cell Counter (Invitrogen, Grand Island, NY, USA), and cells were diluted to the appropriate seeding concentration before being returned to the incubator.

#### 3.3.7. Cell Viability Assay

A431 and HaCaT cells were seeded into 96-well plates at 5000 cells per well and incubated overnight in complete growth medium. Test compounds were initially dissolved in DMSO to prepare 1 M primary stock solutions. Intermediate 10 mM working stock solutions were subsequently prepared and diluted in culture medium to obtain final treatment concentrations of 0.001, 0.01, 0.1, and 1 mM. The final DMSO concentration was maintained at 0.1% (*v*/*v*) in all treatment groups, including vehicle controls. No clinically used antitumor reference drug was included in this preliminary screening study, because the primary aim was to comparatively evaluate the relative cytotoxic activities of the synthesized derivatives under identical experimental conditions. Cells were treated for 24, 48, or 72 h prior to MTT analysis. Control wells received 0.1% DMSO only. At the end of each treatment period, MTT dye (5 mg/mL, Sigma, St. Louis, MO, USA) was added to each well, and cells were incubated for 4 h. After incubation, the medium was removed, and the resulting formazan crystals were solubilized with 100 µL of DMSO. Absorbance was measured at 570 nm using a microplate reader. Percent cell viability was calculated according to Equation (3):

Cell Viability (%) = (*A*_sample_/*A*_control_) × 100 (3)

To minimize random variation, all experiments were conducted in quadruplicate. Data were analyzed using the General Linear Model to assess the effects of cell line, concentration, and time, as well as their interactions.

#### 3.3.8. Scratch Assay-Based Wound Closure Assay

Migration assays were conducted using 12-well plates marked with a fine line on the bottom surface. A431 and HaCaT cells were allowed to reach approximately 80% confluence. Proliferation was then inhibited by incubation with serum-free medium containing mitomycin C (2 µg/mL) for 2 h prior to scratch induction. After mitomycin C pretreatment, two linear scratch wounds were created per well using a P2 pipette tip. Wells were washed three times with 37 °C PBS. Cells were then treated with medium containing 0.01 mM of the respective compound and 2 µg/mL mitomycin C. Scratch wounds were photographed immediately. Images were acquired at 0, 14, and 18 h. After 24 h of treatment, cells were removed from the incubator and fixed with 0.5 mL of ice-cold methanol for 10 min at −20 °C. Methanol was then aspirated, and cells were stained with 0.5 mL crystal violet for 10 min at room temperature. Excess stains were removed via gentle washing several times with deionized water. Plates were air-dried, and images were captured at 10× magnification. For each well, four images were taken at pre-marked intersections. To ensure reproducibility and minimize random variation, all conditions were tested in triplicate wells, yielding a total of 12 images per treatment condition. Wound area was quantified using FIJI/ImageJ (software version 1.54s, National Institutes of Health, Bethesda, MD, USA) by manually outlining the cell-free region, and percent closure was calculated relative to the 0 h time point. Scratch closure for each image was normalized to its control using the following Equation (4):

Scratch Closure (%) = ((*A*_0_ − *A*_t_)/*A*_0_) × 100 (4)

Data were analyzed using the General Linear Model to assess the effects of treatment, time, and cell line on wound closure behavior.

### 3.4. Statistical Analysis

Antioxidant (DPPH, Fe^2+^ chelation, FTC-TAC) and SPF assays were performed in triplicate. For DPPH, percentage inhibition values were arcsine-square-root transformed prior to analysis. Antioxidant and SPF data were analyzed by two-way ANOVA to evaluate the effects of compound and concentration, and Tukey’s HSD post hoc test was used for multiple comparisons. Model assumptions were evaluated using Shapiro–Wilk and Levene tests. Pearson correlation analysis was used to assess associations among SPF and antioxidant endpoints at 10.00 µg/mL.

For the MTT viability assays, absorbance values obtained from treated wells were normalized to the corresponding untreated control group within each cell line and exposure period, and expressed as percentage cell viability. Cytotoxicity was subsequently calculated as the percentage reduction relative to control viability. Data were analyzed using a three-way analysis of variance (ANOVA) to evaluate the effects of cell line, exposure time, and compound concentration, including their interaction terms. When significant differences were detected, post hoc multiple comparisons were performed using Tukey’s honestly significant difference (HSD) test. Assumptions of normality and homogeneity of variance were evaluated using the Shapiro–Wilk and Levene’s tests, respectively. For percentage-based datasets, arcsine square-root transformation was applied prior to ANOVA to improve variance stabilization. IC_50_ values were estimated by nonlinear regression analysis using concentration–response curves, and selectivity indices (SI) were calculated by comparing IC_50_ values between HaCaT and A431 cells.

For the scratch wound closure assays, wound areas were measured at 0, 14, and 18 h using image-based analysis, and migration-associated wound closure responses were calculated relative to the initial wound area at 0 h. Lower residual wound area values were interpreted as increased wound closure under the experimental conditions. Statistical evaluation was performed using factorial ANOVA/General Linear Model approaches to assess the effects of cell line, treatment condition, and time on wound closure responses. Pairwise comparisons between each treated group and the corresponding control were performed using Welch’s *t*-test within each cell line and time point. Tukey’s HSD test was used for post hoc pairwise comparisons when appropriate. Data are presented as mean ± standard deviation (SD), and statistical significance was accepted at *p* < 0.05. All statistical analyses were performed using IBM SPSS Statistics for Windows, Version 26.0 (IBM Corp., Armonk, NY, USA).

## 4. Conclusions

In this study, six novel pyrazole-based carbohydrazone derivatives bearing different heteroaromatic substituents were successfully synthesized and structurally characterized using ATR-FTIR, ^1^H NMR, APT-^13^C NMR, and HRMS analyses. The synthesized compounds were evaluated through a multifunctional screening approach involving antioxidant, photoprotective, CT-DNA-associated spectroscopic, cytotoxicity, and scratch wound closure assays. The results demonstrated that heteroaromatic substitution influenced the behavior of the pyrazole–carbohydrazone scaffold across multiple experimental systems.

Distinct substituent-dependent activity profiles were observed among the synthesized derivatives. The benzothiazole-containing derivative **3f** exhibited the strongest DPPH radical scavenging activity, ferric thiocyanate antioxidant capacity, and SPF response, while also showing a pronounced CT-DNA-associated spectroscopic response under the experimental conditions employed. In contrast, the benzimidazole derivative **3e** displayed the highest Fe^2+^ chelation activity, indicating that nitrogen-rich heteroaromatic systems may contribute favorably to metal-chelating behavior. Cell-based studies further demonstrated that the imidazole- and thiazole-containing derivatives **3b** and **3c** provided the most favorable balance between growth-inhibitory potency and selectivity toward A431 epidermoid carcinoma cells relative to HaCaT keratinocytes. Scratch assay findings did not support direct anti-migratory activity under the tested conditions but suggested compound-dependent modulation of migration-associated wound closure behavior.

Overall, the present findings demonstrate that heteroaromatic modification of pyrazole-based carbohydrazones provides an effective strategy for tuning redox behavior, CT-DNA-associated spectroscopic behavior, photoprotective properties, and cytotoxic selectivity. These results highlight the pyrazole–carbohydrazone framework as a structurally adaptable platform for future bioactivity-oriented optimization studies. Further investigations involving optimized DNA-spectroscopic titration conditions, apoptosis-related pathways, intracellular ROS generation, protein-expression analyses, and expanded cellular models may help clarify the molecular basis of the observed biological responses.

## Figures and Tables

**Table 1:** Qualitative UV-Vis spectral responses of compounds **3c**–**3f** following incremental addition of CT-DNA in Tris-HCl buffer (pH 7.4). Abbreviations: ↑, increase in absorbance; ↓, decrease in absorbance.

Compound	Monitoring Wavelength (nm)	Spectral Response Observed Upon CT-DNA Addition
**3c**	322	↑ Mild increase in absorbance
**3d**	330	↓ Decrease in absorbance with bathochromic shift
**3e**	330	↑ Mild increase in absorbance with minimal wavelength shift
**3f**	330	↓ Pronounced decrease in absorbance with bathochromic shift

**Table 2:** Relative fluorescence emission responses observed upon incremental addition of CT-DNA to compounds **3c**–**3f** under physiological buffer conditions (10 mM Tris-HCl, 50 mM NaCl, pH 7.4). Fluorescence responses are presented as maximum F/F_0_ values, where F_0_ and F correspond to emission intensities measured in the absence and presence of CT-DNA, respectively.

Compound	λex (nm)	λem (nm)	Maximum F/F_0_	Observed Fluorescence Response
**3c**	323	647	2.47	Moderate fluorescence enhancement
**3d**	329	659	12.82	Marked fluorescence enhancement
**3e**	330	662	9.01	Marked fluorescence enhancement
**3f**	330	662	10.13	Marked fluorescence enhancement

**Table 3:** Structure–activity relationship summary of compounds **3a**–**3f** based on cytotoxicity and selectivity profiles in A431 and HaCaT cells.

Compound	Overall Cytotoxic Potency	Selectivity Towards A431 Cells	Most Favorable Time Point (h)	Key Biological Features	Proposed SAR Interpretation
**3a**	Moderate	Moderate	48	Dose-dependent viability reduction with moderate preferential suppression of A431 cells.	Baseline scaffold demonstrates measurable growth-inhibitory activity with moderate cancer-cell selectivity.
**3b**	High	Moderate-High	72	Strong cytotoxicity and sustained suppression of A431 viability during prolonged exposure.	Structural modifications may contribute to enhanced potency and preferential activity toward malignant keratinocytes.
**3c**	High	Moderate	72	Sustained time-dependent cytotoxicity with pronounced suppression of A431 cells.	Structural features may be associated with prolonged growth-inhibitory activity during extended exposure.
**3d**	High	Poor	48	Strong time- and concentration-dependent cytotoxicity.	Structural variation may contribute to broader nonspecific cytotoxicity.
**3e**	Moderate-High	Variable	48	Strong cytotoxic activity with highly time-dependent selectivity.	Structural modifications may partially improve selectivity while maintaining strong growth-inhibitory potency.
**3f**	Moderate-High	Moderate	48	Strong cytotoxicity accompanied by increased HaCaT sensitivity at prolonged exposure.	Structural features preserve cytotoxic potency but with reduced selectivity toward malignant cells.

**Table 4:** IC_50_ values (mM) and selectivity index (*SI*) for compounds **3a**–**3f** in A431 and HaCaT cells.

Compound	Time (h)	A431 IC_50_ (mM)	HaCaT IC_50_ (mM)	SI
**3a**	24	0.763	>1.000	>1.31
	48	0.193	0.661	**3.42**
	72	0.472	0.628	1.33
**3b**	24	0.521	0.119	0.23
	48	0.146	0.412	**2.82**
	72	0.104	0.171	1.64
**3c**	24	>1.000	>1.000	ND
	48	0.211	0.371	1.76
	72	0.148	0.284	1.92
**3d**	24	0.328	0.117	0.36
	48	0.086	0.054	0.62
	72	0.200	0.026	0.13
**3e**	24	0.236	0.614	**2.60**
	48	0.0068	0.248	**36.47**
	72	0.041	0.0071	0.17
**3f**	24	0.417	0.338	0.81
	48	0.083	0.041	0.49
	72	0.051	0.020	0.39

ND: not determinable. SI = IC_50_(HaCaT)/IC_50_(A431).

## Data Availability

The original contributions presented in this study are included in the article/Supplementary Materials. Further inquiries can be directed to the corresponding author.

## Supplementary Materials

The following supporting information can be downloaded at https://www.mdpi.com/article/10.3390/molecules31122031/s1, Figures S1–S6: FTIR spectra; Figures S7–S12: 1H NMR spectra; Figures S13–S18: APT-13C NMR spectra; Figures S19–S24: HRMS spectra; Tables S1–S6: The complete HRMS peak-list tables; Table S7: Conversion of mass-based concentrations (µg/mL) to molar units (µM and mM) for compounds; Table S8: in vitro DPPH free radical scavenging activity of compounds; Table S9: in vitro Ferrous ion chelating activity of compounds; Table S10: Total antioxidant capacity of compounds; Table S11: Total antioxidant capacity of compounds determined by the ferric thiocyanate (FTC) method at 36 h; Figure S25: Time-dependent absorbance profiles of compounds at 10 µg/mL; Table S12: in vitro Sun protection factor (SPF) values of compounds; Table S13: Pearson correlation coefficients among SPF, DPPH radical scavenging, Fe2+ chelation, and FTC; Figures S26–S31: UV-Vis absorption spectra of compounds in the presence of CT-DNA; Figures S32–S35: https://doi.org/10.1007/s11696-020-01106-4 Fluorescence emission spectra of compounds in the presence of CT-DNA; Figures S36–S41: Cell Viability Plots of compounds; Table S14: Three-way ANOVA summary; Table S15: Numerical MTT cell viability data; Figures S42–S47: Wound closure plots for all compounds.
