# Supplementary material for: Heteroaromatic Pyrazole-Based Carbohydrazones: Structure-Dependent Redox Activity, DNA-Associated Spectroscopic Behavior, and Multifunctional Biological Properties
Source: Molecules. 2026 Jun 10;31(12):2031. doi: 10.3390/molecules31122031 (PMC13304666; doi:10.3390/molecules31122031)
Supplement: Supplementary file 1 [file molecules-31-02031-s001.zip › molecules-4338723-supplementary.pdf]

SUPPLEMENTARY INFORMATION

Heteroaromatic Pyrazole-Based Carbohydrazones: Structure-Dependent Redox Activity, DNA-Associated Spectroscopic Behavior, and Multifunctional Biological Properties

Aliye Gediz Erturk*, Ertuğrul Yiğit

Department of Chemistry, Faculty of Science & Arts, Ordu University, 52200 Ordu, Turkiye

Correspondence: aliyeerturk@gmail.com

Table of contents

FTIR spectra of compounds **3a-3f** (Figure S1-S6) 1

^{1}H NMR spectra of compounds **3a-3f** (Figure S7-S12) 5

APT-^{13}C NMR spectra of compounds **3a-3f** (Figure S13-S18) 8

HRMS spectra of compounds **3a-3f** (Figure S19-S24) 11

The complete HRMS peak-list tables of compounds **3a-3f** (Table S1-S6) 13

Conversion of mass-based concentrations (µg/mL) to molar units (µM and mM) for compounds **3a-3f** (Table S7) 19

In vitro DPPH free radical scavenging activity of pyrazole-based carbohydrazones (Table S8) 20

In vitro Ferrous ion chelating activity of pyrazole-based carbohydrazones (Table S9) 20

Total antioxidant capacity of pyrazole-based carbohydrazones (**3a-3f**) determined by the ferric thiocyanate method at the 36 h (Table S10) 21

Total antioxidant capacity of pyrazole-based carbohydrazones (**3a-3f**) determined by the ferric thiocyanate (FTC) method at 36 h(Table S11) 21

Time-dependent absorbance profiles of compounds **3a-3f** at 10 µg/mL in the FTC assay over a 60 h incubation period, measured at 500 nm. (Figure S25) 22

In vitro Sun protection factor (SPF) values of pyrazole-based carbohydrazones (**3a-3f**) at different concentrations. (Table S12) 23

Pearson correlation coefficients among SPF, DPPH radical scavenging, Fe^{2+} chelation, and FTC antioxidant activity values at 10.00 µg/mL for compounds **3a-3f** (n = 6). (Table S13) 23

UV-Vis absorption spectra of compounds **3a-3f** in the presence of CT-DNA (Figure S26-S31) 24

Fluorescence emission spectra of compounds **3c-3f** in the presence of CT-DNA (Figure S32-S35) 30

Cell Viability Plots of compounds **3a-3f** (Figure S36-S41) 34

Three-way ANOVA summary for normalized MTT cell viability in A431 and HaCaT cells treated with compounds **3a-3f** (Table S14) 37

Numerical MTT cell viability data of compouns **3a-3f** (Table S15) 38

Wound closure plots for all compounds **3a-3f** (Figure S42-S47) 39

FTIR spectra of compounds 3a-3f

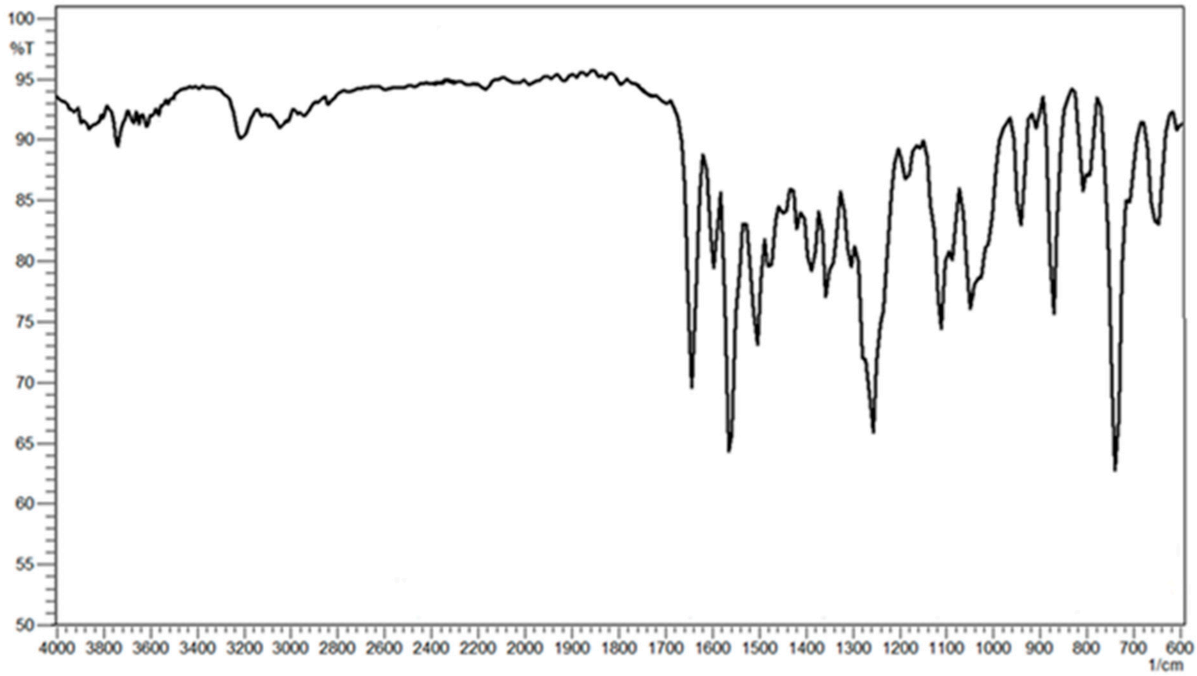

Figure S1. FTIR spectrum of compound **3a**.

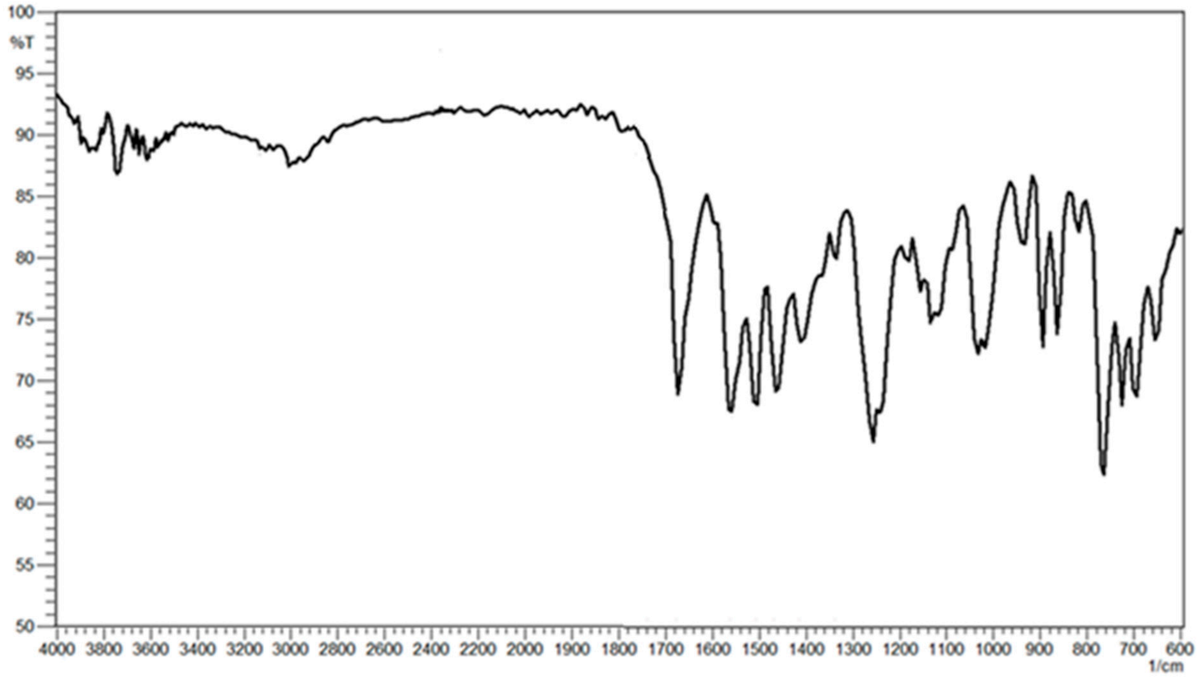

Figure S2. FTIR spectrum of compound **3b**

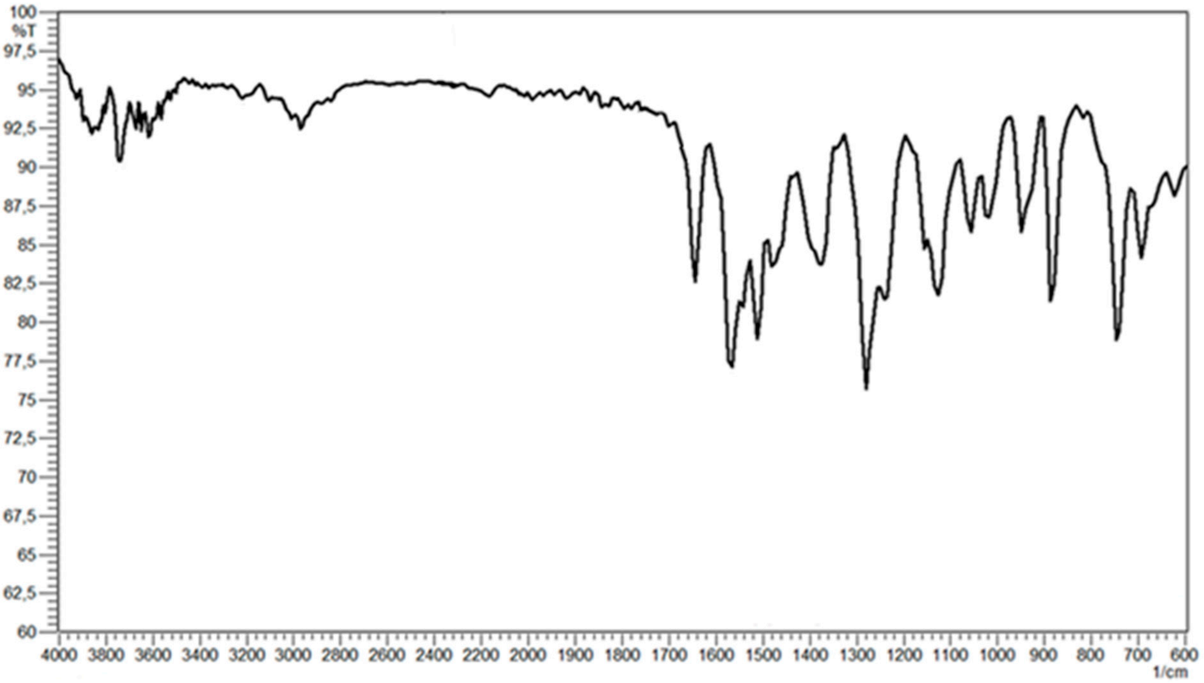

Figure S3. FTIR spectrum of compound **3c**

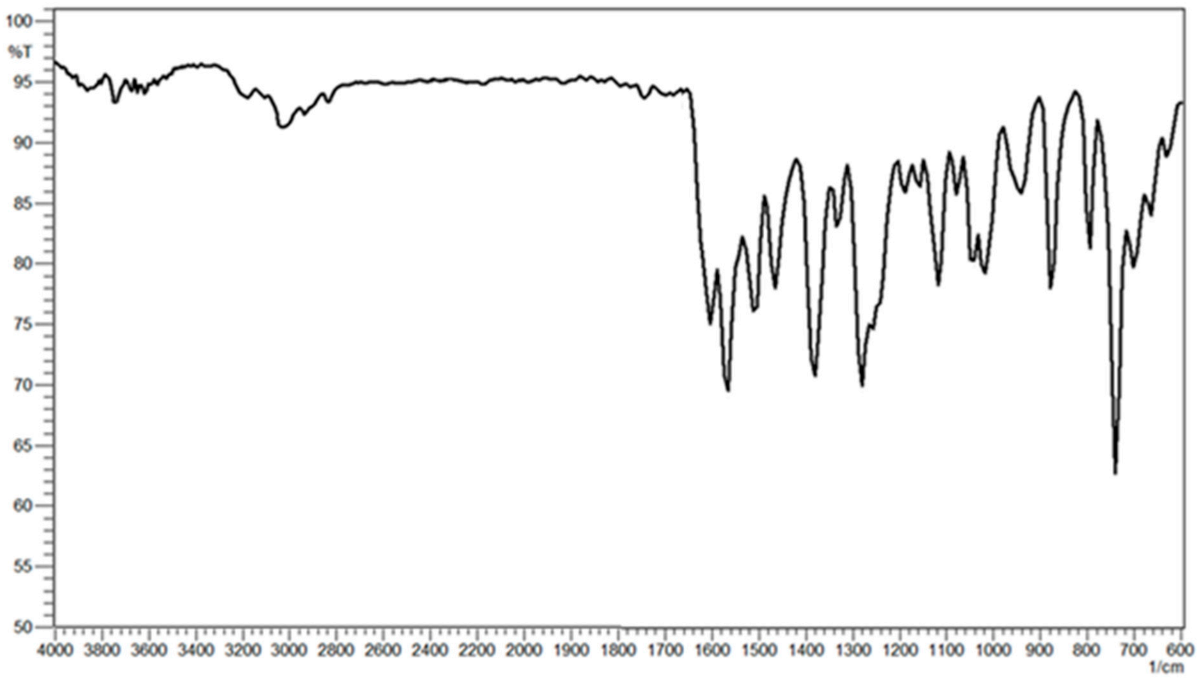

Figure S4. FTIR spectrum of compound **3d**

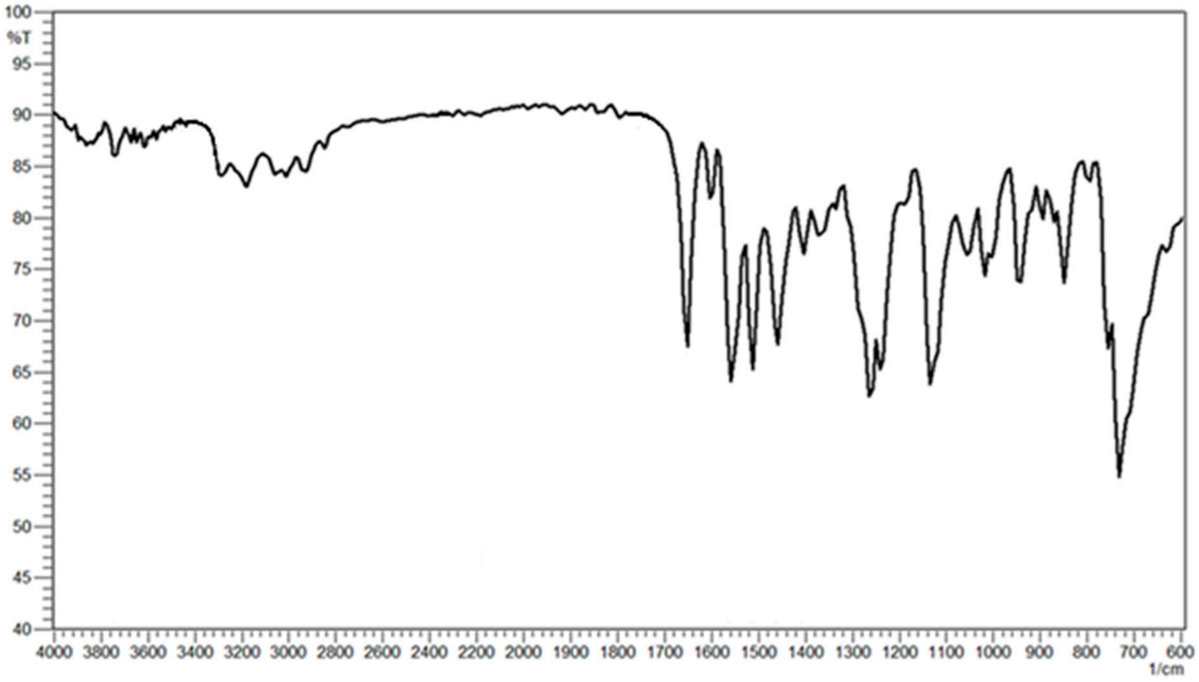

Figure S5. FTIR spectrum of compound **3e**

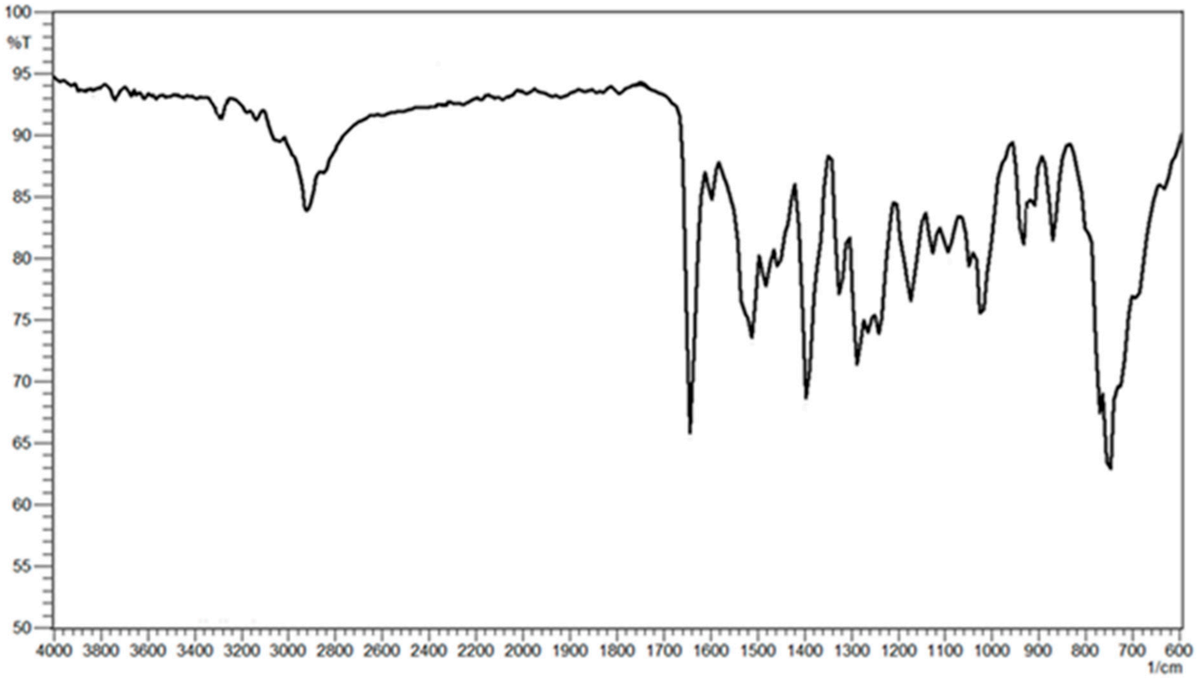

Figure S6. FTIR spectrum of compound **3f**

^{1}H NMR spectra of compounds 3a-3f

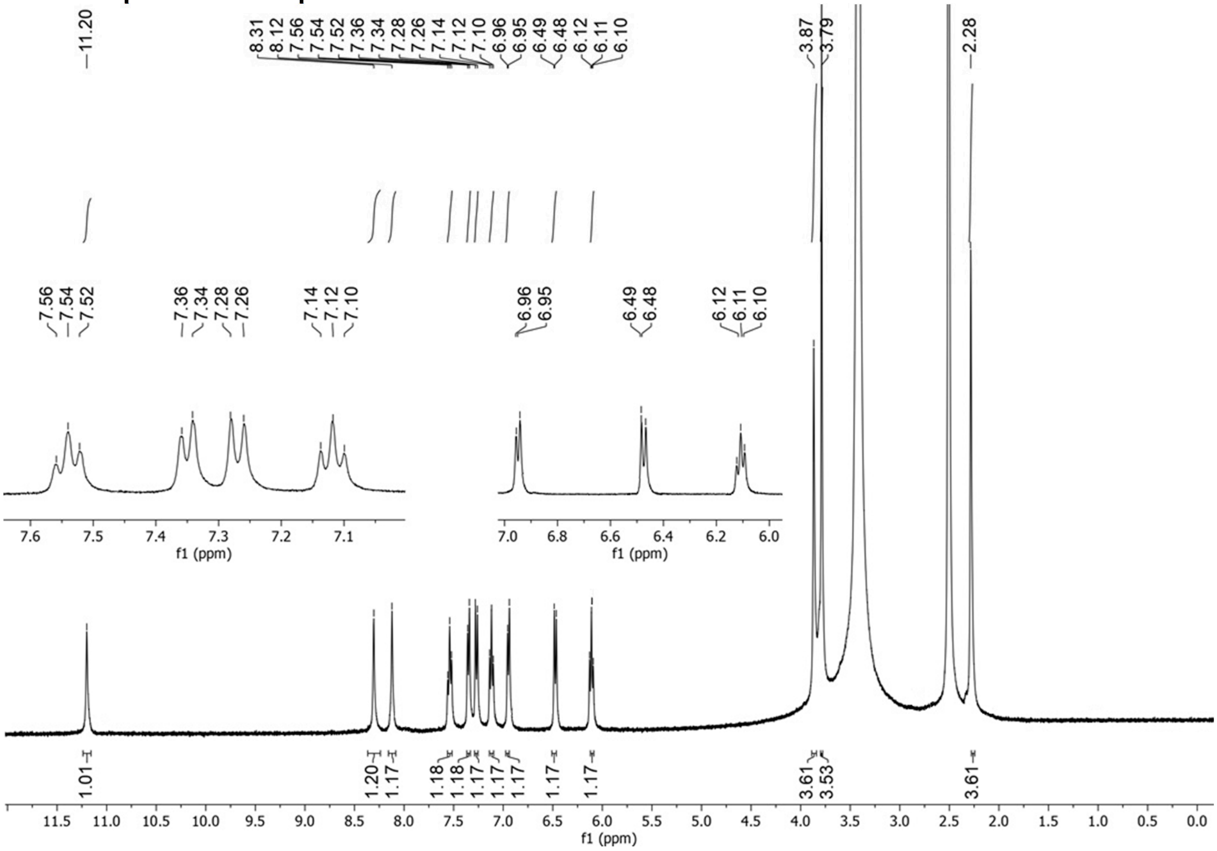

Figure S7. ^{1}H NMR spectrum of compound **3a** in DMSO-d_6.

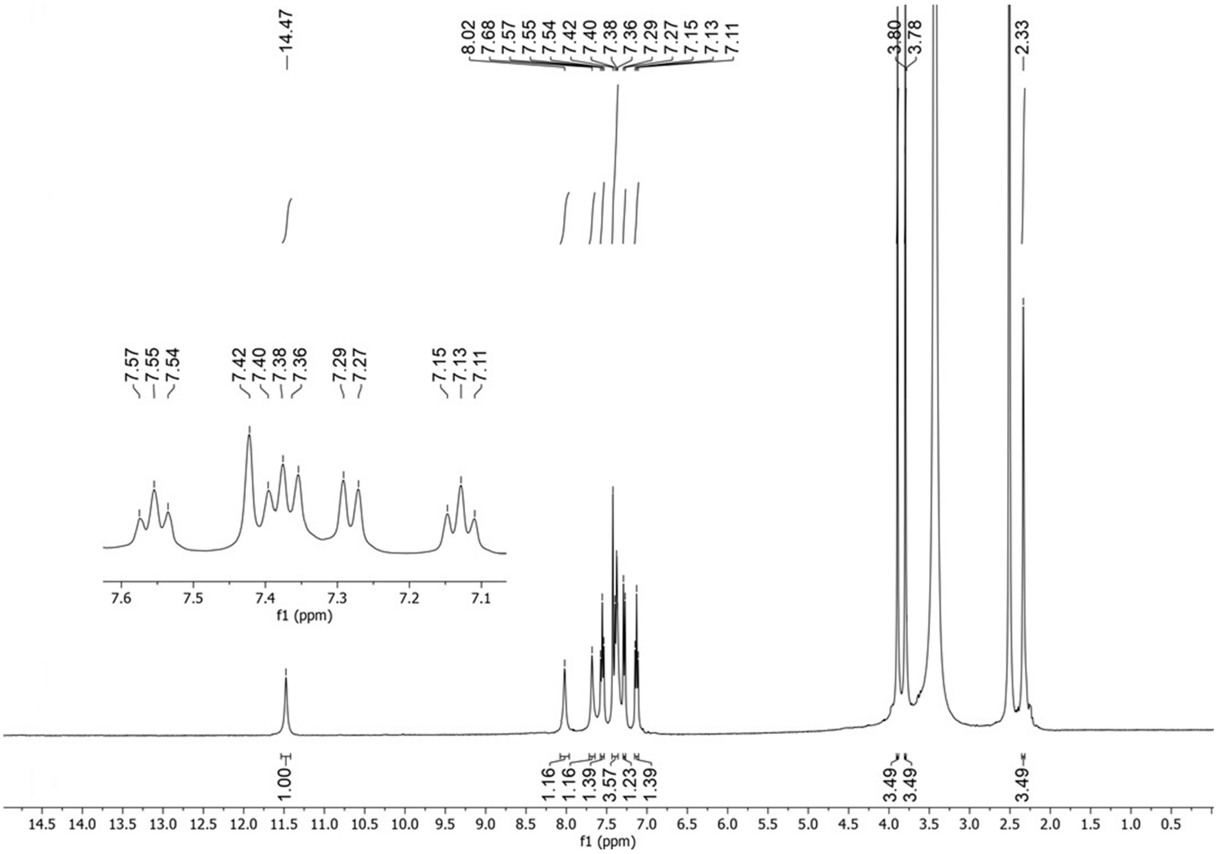

Figure S8. ^{1}H NMR spectrum of compound **3b** in DMSO-d_6.

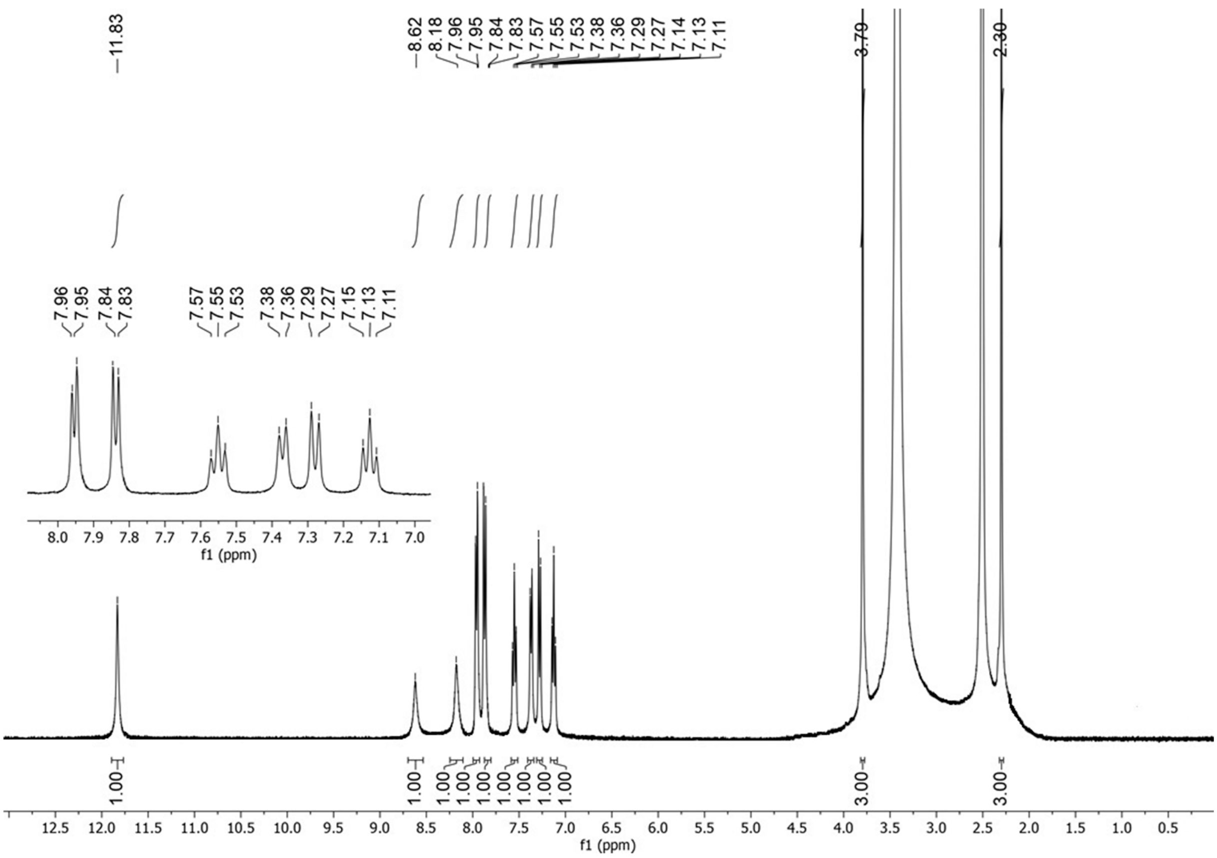

Figure S9. ^{1}H NMR spectrum of compound **3c** in DMSO-d_6.

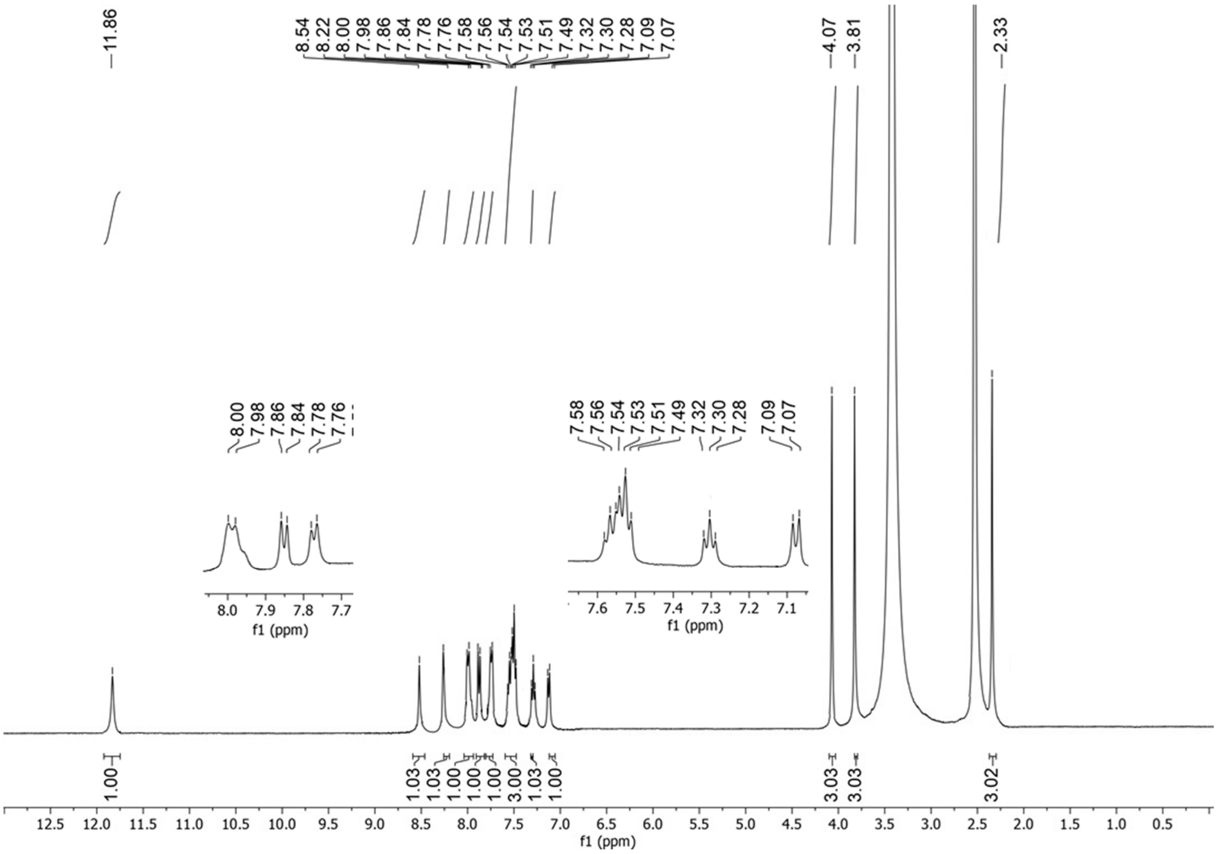

Figure S10. ^{1}H NMR spectrum of compound **3d** in DMSO-d_6.

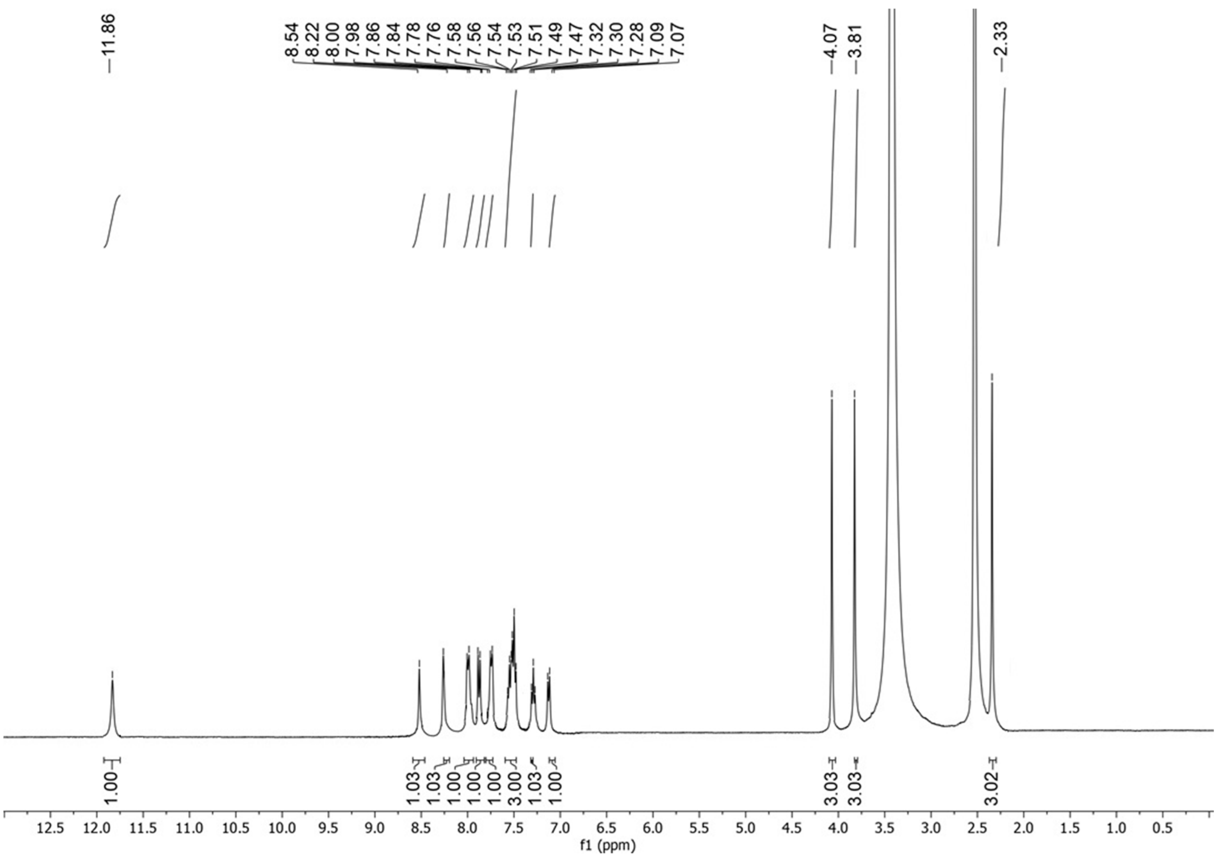

Figure S11. ^{1}H NMR spectrum of compound **3e** in DMSO-d_6.

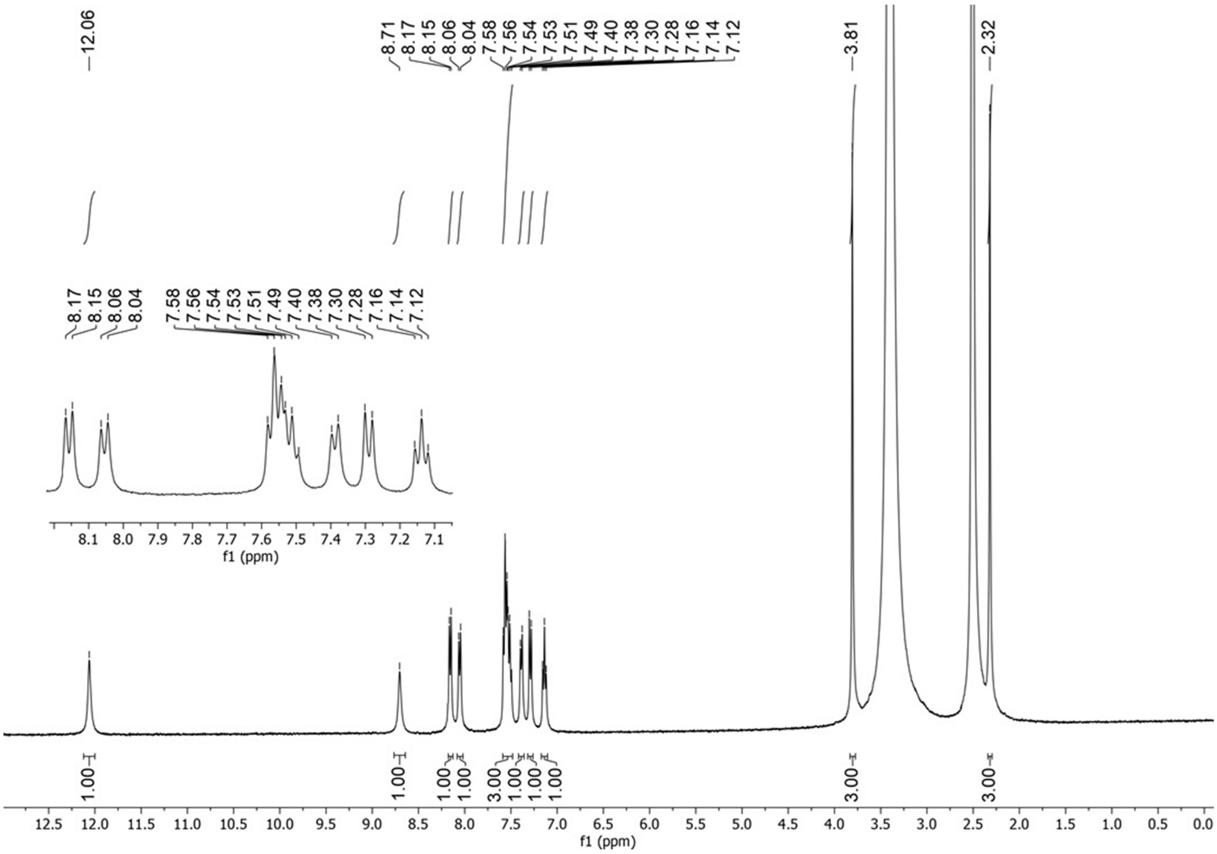

Figure S12. ^{1}H NMR spectrum of compound **3f** in DMSO-d_6.

APT-^{13}C NMR spectra of compounds 3a-3f

The observed APT ^{13}C NMR signals were in agreement with the number and types of carbon atoms expected for the proposed structures.

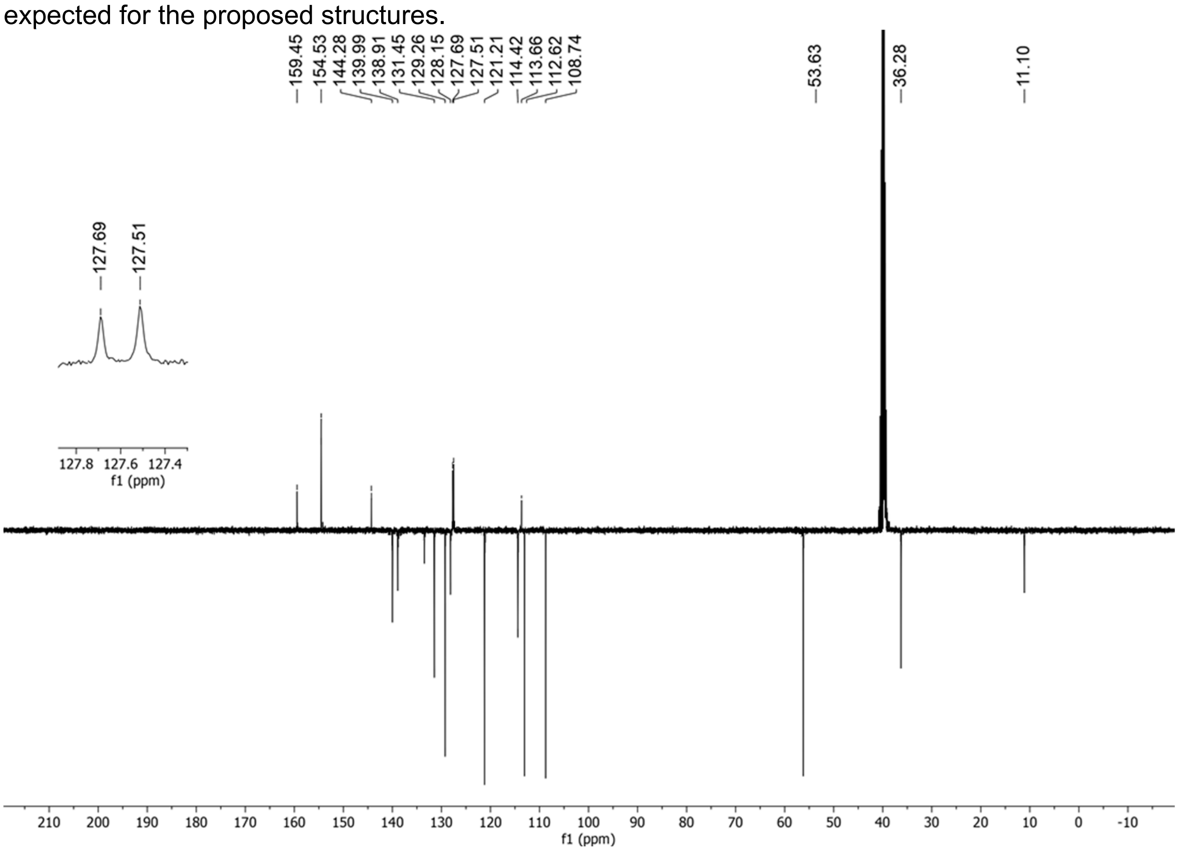

Figure S13. APT-^{13}C NMR spectrum of compound **3a** in DMSO-d_6

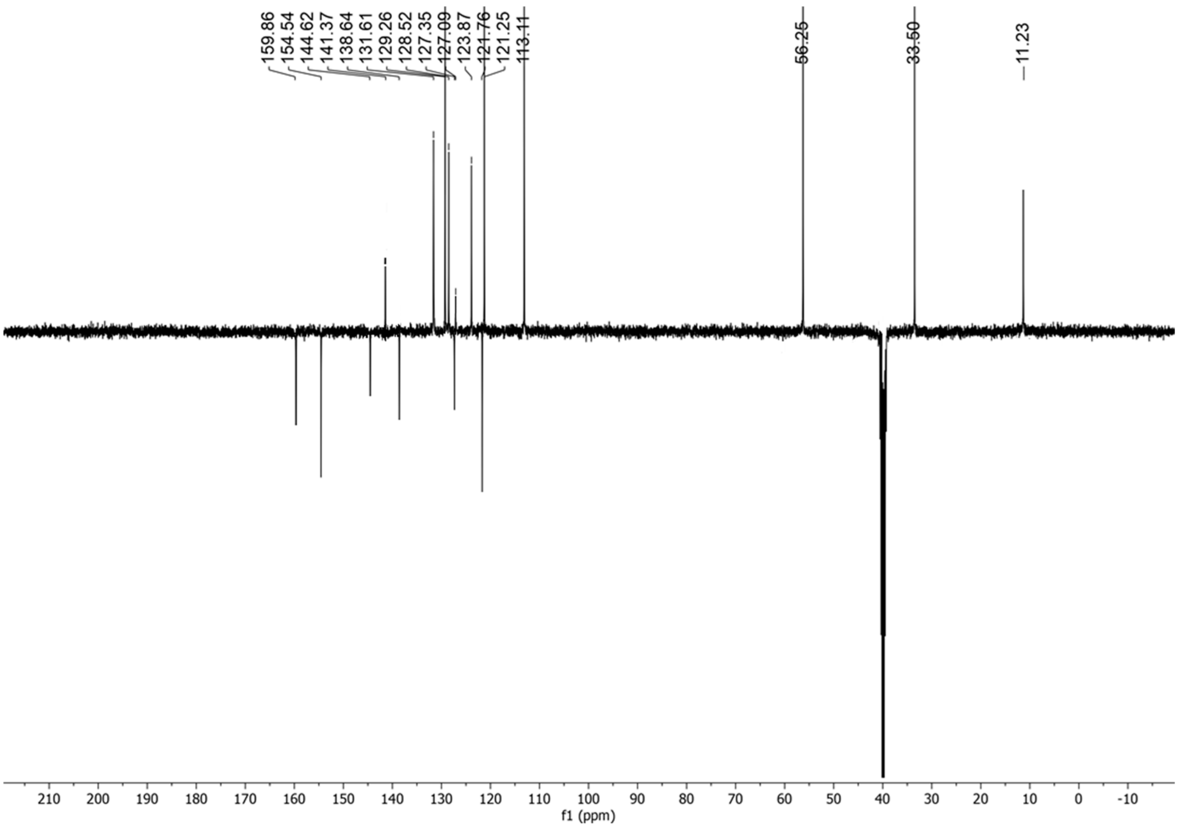

Figure S14. APT-^{13}C NMR spectrum of compound **3b** in DMSO-d_6

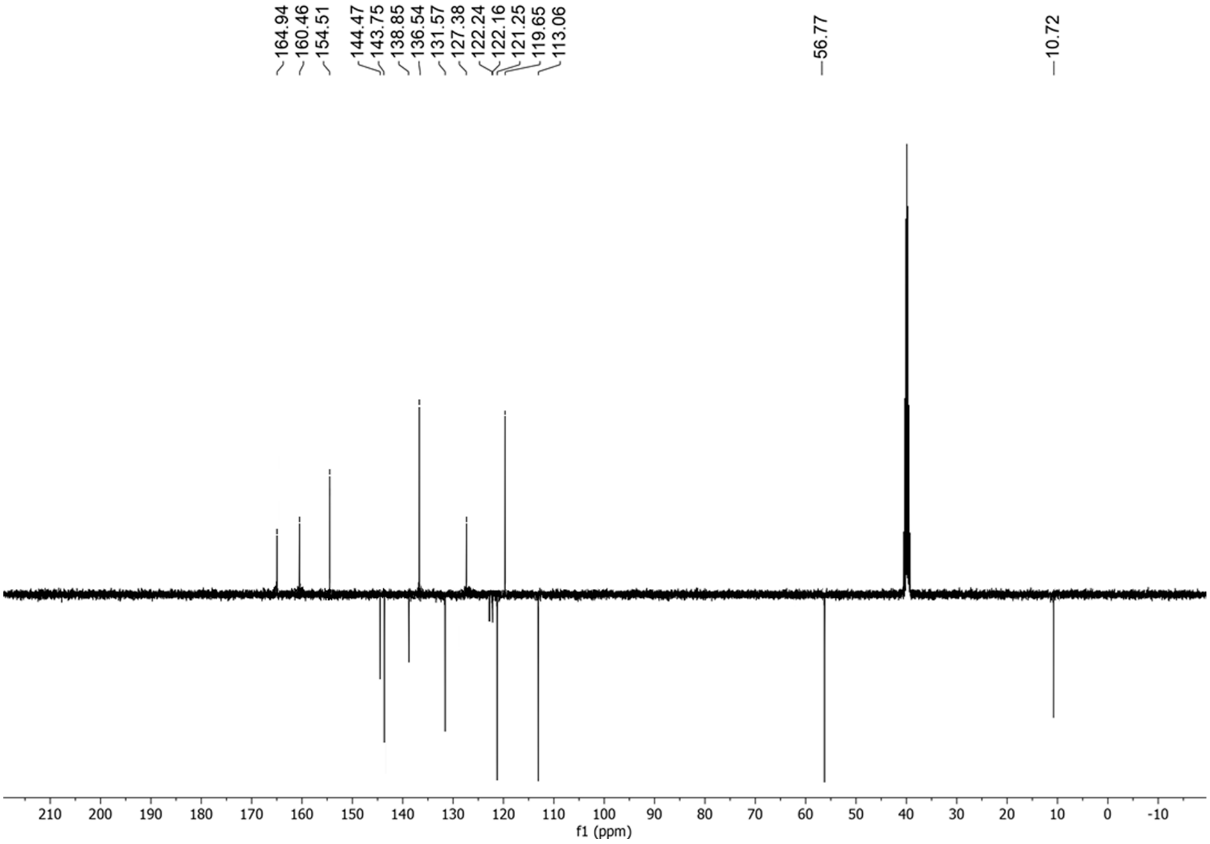

Figure S15. APT-^{13}C NMR spectrum of compound **3c** in DMSO-d_6

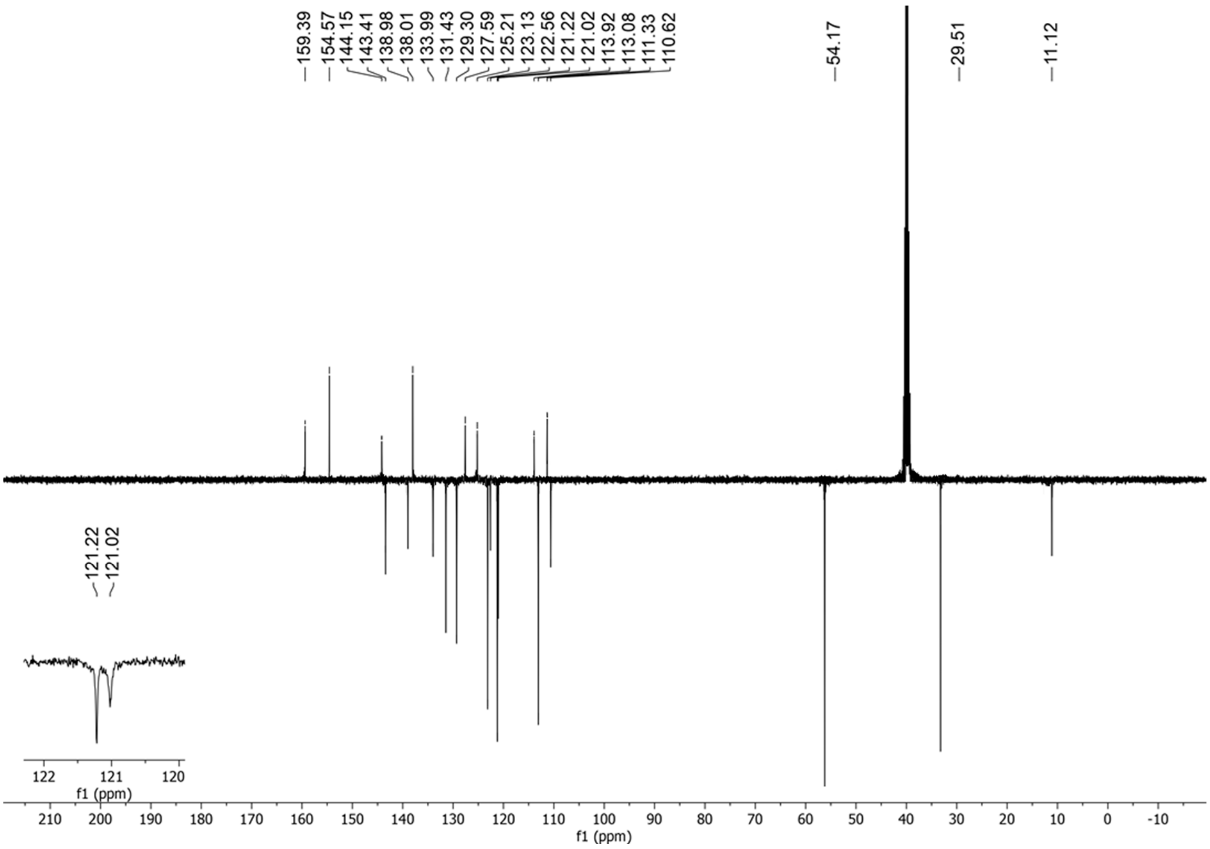

Figure S16. APT-^{13}C NMR spectrum of compound **3d** in DMSO-d_6

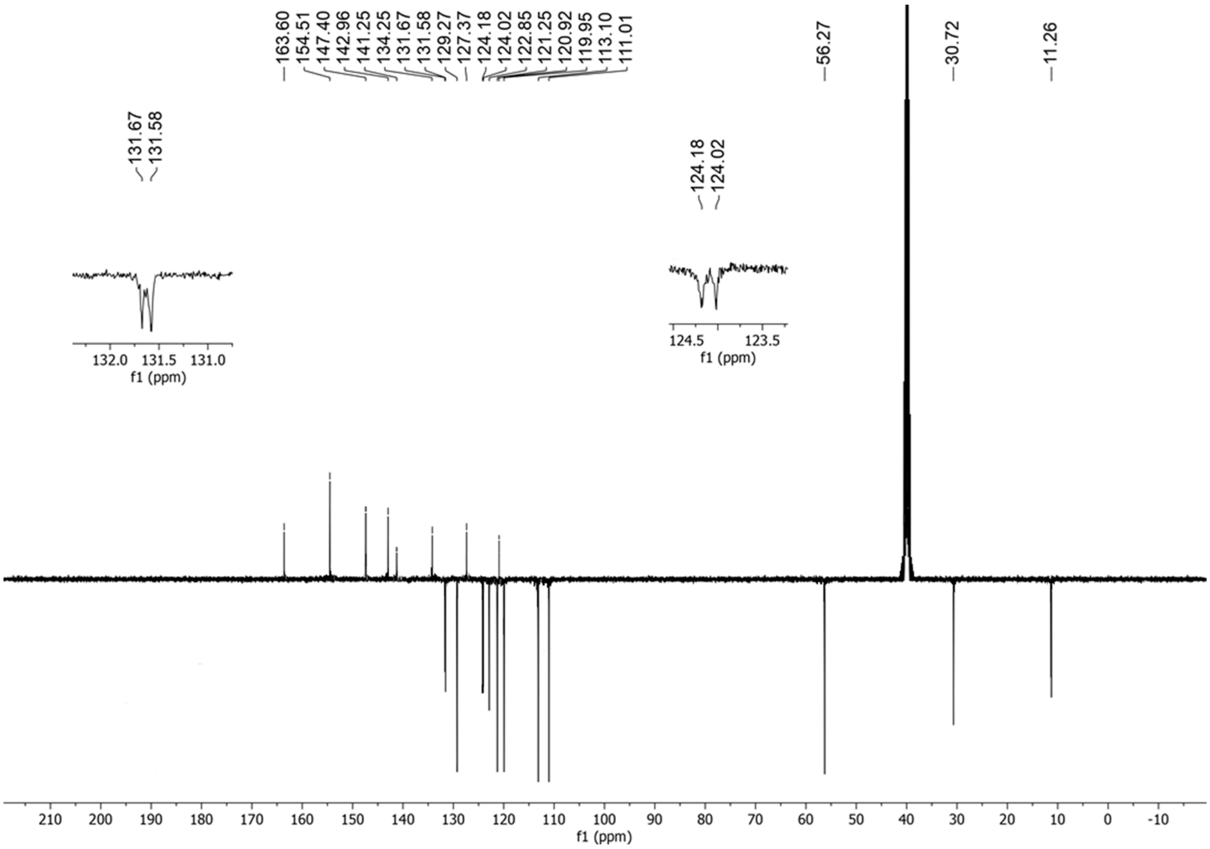

Figure S17. APT-^{13}C NMR spectrum of compound **3e** in DMSO-d_6.

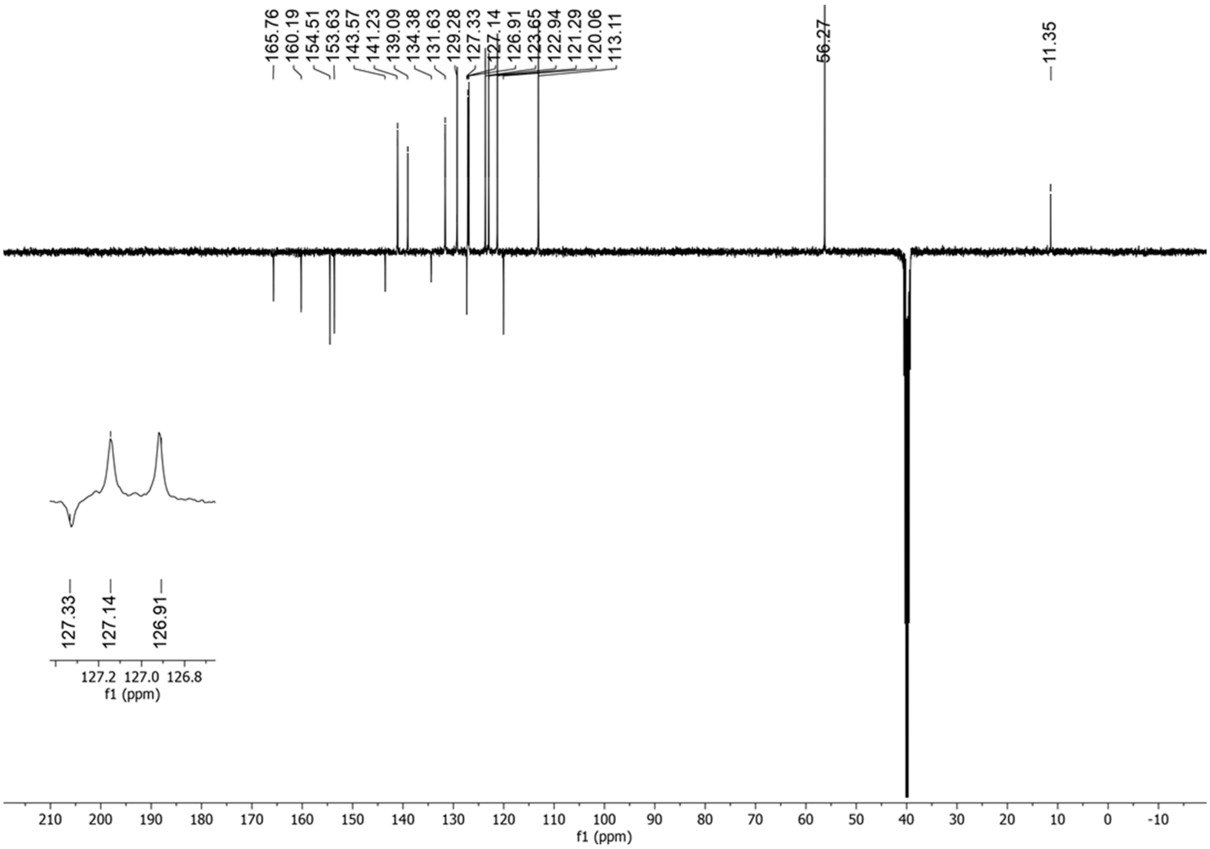

Figure S18. APT-^{13}C NMR spectrum of compound **3f** in DMSO-d_6

HRMS spectra of compounds 3a-3f

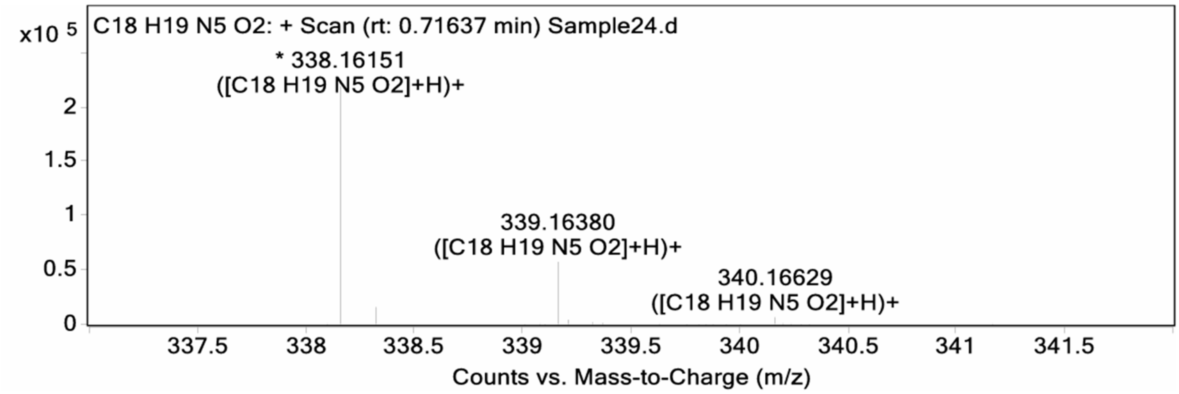

Figure S19. HRMS (ESI) spectrum of compound **3a**. Calculated for $C_{18}H_{20}N_5O_2^+$ $[M+H]^+$: m/z 338.16115; found: m/z 338.16151.

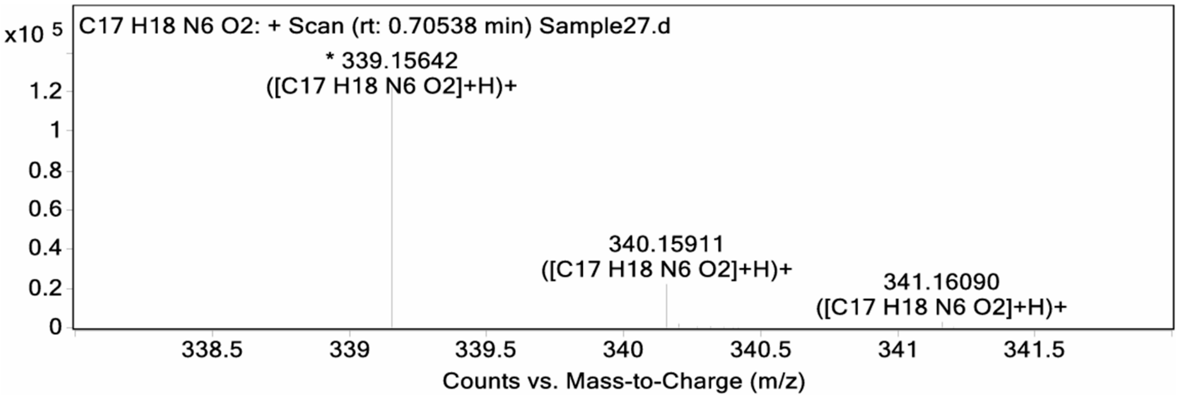

Figure S20. HRMS (ESI) spectrum of compound **3b**. Calculated for $C_{17}H_{19}N_6O_2^+$ $[M+H]^+$: m/z 339.15640; found: m/z 339.15642.

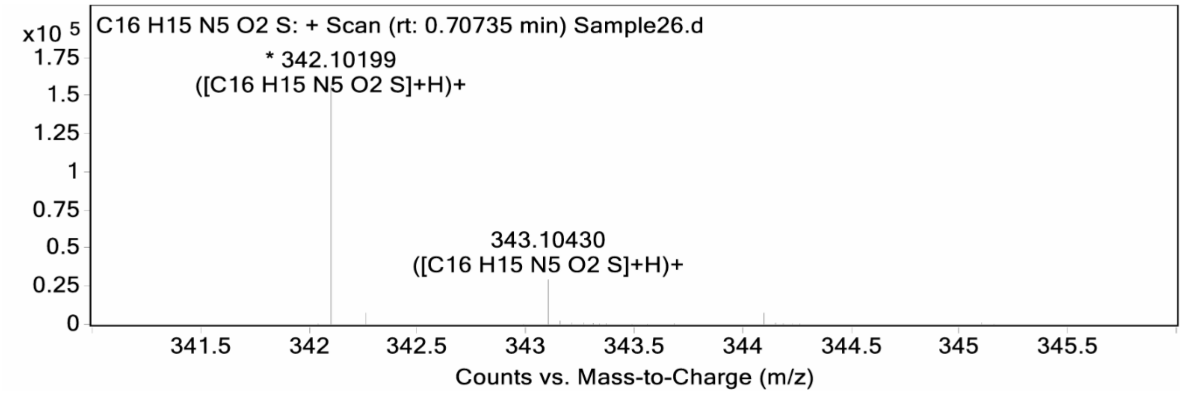

Figure S21. HRMS (ESI) spectrum of compound **3c**. Calculated for $C_{16}H_{16}N_5O_2S^+$ $[M+H]^+$: m/z 342.10192; found: m/z 342.10199.

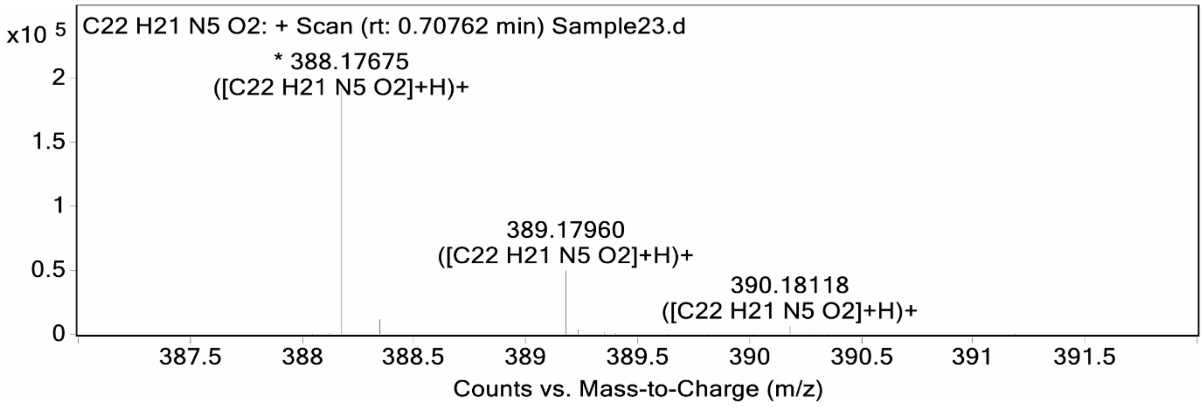

Figure S22. HRMS (ESI) spectrum of compound **3d**. Calculated for $C_{22}H_{22}N_5O_2^+$ $[M+H]^+$: m/z 388.17680; found: m/z 388.17675.

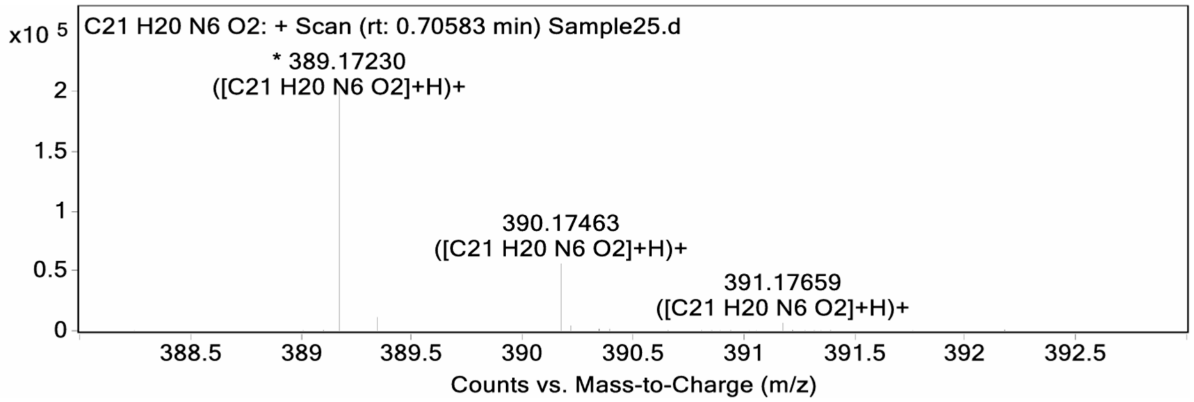

Figure S23. HRMS spectrum of compound **3e**. Calculated m/z for $C_{21}H_{21}N_6O_2^+$ $[M+H]^+$: 389.17205; found: 389.17230.

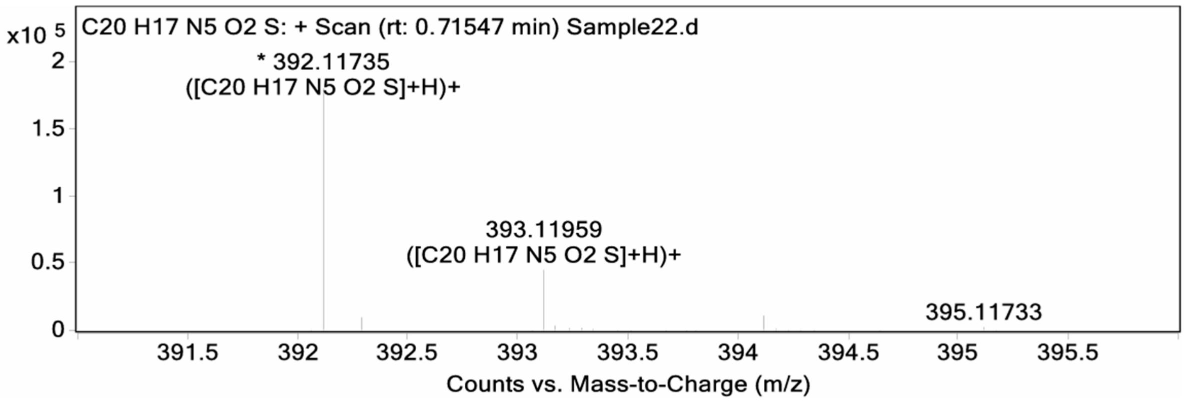

Figure S24. HRMS spectrum of compound **3f**. Calculated m/z for $C_{20}H_{18}N_5O_2S^+$ $[M+H]^+$: 392.11757; found: 392.11735.

The complete HRMS peak-list tables of compounds 3a-3f

Table S1. Complete HRMS peak-list data for compound **3a**

m/z	z	Abund	Formula	Ion
79.02175		56003.24		
79.03999		3919.91		
79.05103		3069.01		
102.12795		27390.07		
157.03503		15168.28		
338.16151	1	218528.08	C18 H19 N5 O2	(M+H)+
338.32166		15538.78		
339.1638	1	56857.37	C18 H19 N5 O2	(M+H)+
339.21247		4028.35		
340.16629	1	6203.69	C18 H19 N5 O2	(M+H)+
360.14231	1	62001.64		
360.18655		4658.56		
360.30788		2355.47		
361.14502	1	11235.23		
376.11714	1	16081.78		
377.12023	1	3657.99		
439.28081		4510.5		
694.2884	2	3243.65		
694.78833	2	3641.03		
697.30039	1	235003.67		
697.52293	1	25259.02		
697.59166		17400.24		
698.29878	1	178870.5		
698.52614	1	8219.92		
699.30076	1	40913.27		
699.36238		4348.08		
700.30371	1	7332.32		
729.23535		4753.24		
731.235		3016.29		
765.20977	1	9372.68		
766.21057	1	4348.47		
767.20799	1	3956.46		
1066.38307	1	3666.7		
1067.38719	1	2884.75		

Table S2. Complete HRMS peak-list data for compound **3b**

m/z	**z**	**Abund**	**Formula**	**Ion**
64.01686		3256.02		
79.02206		26112.16		
102.12787	1	58441.78		
102.15459		3933.87		
102.21748		2270.28		
103.13137	1	4496.11		
157.03437		4439.85		
339.15642	1	122406.26	C17 H18 N6 O2	(M+H)+
340.15911	1	22093.8	C17 H18 N6 O2	(M+H)+
341.1609	1	2873.7	C17 H18 N6 O2	(M+H)+
361.13839	1	24470.12		
361.18267		2667.53		
362.14063	1	4248.9		
440.27616	1	15248.87		
441.27944	1	4644.08		
699.28713	1	208732.23		
699.51424	1	12764.43		
700.28989	1	97639.09		
700.51692	1	3585.41		
701.29038	1	19525.12		
701.35657		2284.51		
702.29506	1	2951.64		
715.26381		3184.48		
767.19973	1	12442.63		
768.20408	1	4748.44		
769.19835	1	5645.41		
770.20189	1	2239.29		
1116.39245		2392.44		

Table S3. Complete HRMS peak-list data for compound **3c**

m/z	z	Abund	Formula	Ion
64.0169		2901.4		
79.02156		24399.2		
79.04008		1941.6		
79.05085		1684.41		
102.12814	1	62487.2		
102.15328		4106.83		
102.2169		2351.53		
103.13066	1	3838.02		
157.03586		5183.98		
215.08107		4436.11		
342.10199	1	156454.72		
342.26338		7495.57		
343.1043	1	29305.27	C16 H15 N5 O2 S	(M+H)+
343.15825		2298.12		
344.1003	1	7531.52	C16 H15 N5 O2 S	(M+H)+
364.08391	1	147671.16		
365.08634	1	26891.8		
365.12988		2583.18		
366.08124	1	7829.13		
380.05869	1	11142.27		
381.05874	1	2451.41		
387.16021		1970.78		
415.19114		3098.8		
705.17781	1	78801.23		
705.2567	1	5972.89		
706.18035	1	30426.51		
706.24739	1	3590.59		
707.17757	1	11003.92		
708.1751	1	3297.14		
737.11525	1	7834.19		
738.11689	1	3630.26		
739.11592	1	2869.5		
1100.19061	1	2769.22		
1101.19219	1	1727.49		
1102.19267	1	1674.11		

Table S4. Complete HRMS peak-list data for compound **3d**

m/z	**z**	**Abund**	**Formula**	**Ion**
64.01728		2512.63		
79.02171		23639.31		
102.12788	1	48225.15		
102.15363		3495.79		
103.13189	1	2674.65		
157.03482		5643.11		
388.17675	1	192417.11	C22 H21 N5 O2	(M+H)+
388.34855		11541.84		
389.1796	1	49733.18	C22 H21 N5 O2	(M+H)+
389.23314		3820.34		
390.18118	1	6358.72	C22 H21 N5 O2	(M+H)+
410.15881	1	10345.83		
411.16011	1	2671.13		
426.13208		4280.13		
489.29639	1	20541.42		
490.30049	1	6254.99		
775.34512	1	4692.4		
776.3478	1	2679.97		
797.32791	1	201117.52		
797.57019		11069.5		
798.32941	1	111301.15		
799.33284	1	27009.02		
799.39836		2655.51		
800.33489	1	4778.31		
813.30509		3326.68		
865.24132		3050.38		

Table S5. Complete HRMS peak-list data for compound **3e**

m/z	**z**	**Abund**	**Formula**	**Ion**
64.01652		3381.77		
79.0219		24615.16		
102.12817	1	50927.86		
102.15349		3840.66		
103.13138	1	3618.81		
157.03478		5815.13		
389.1723	1	202918.52	C21 H20 N6 O2	(M+H)+
389.34354		11275.19		
390.17463	1	55999.68	C21 H20 N6 O2	(M+H)+
390.22048		4353.06		
391.17659	1	6544.14	C21 H20 N6 O2	(M+H)+
411.15407	1	35207.48		
412.15608	1	6684.68		
427.12807		5656.25		
490.29054		3431.72		
799.31783	1	100222.45		
800.32084	1	45001.95		
800.40542		3833.92		
801.3223	1	11780.02		
830.24877		4500.53		
831.2552	1	14466.31		
832.25941	1	6703.75		
833.25786	1	2933.35		
1241.40157	1	3513.81		
1242.40608	1	2512.39		
1243.40674	1	2496.82		

Table S6. Complete HRMS peak-list data for compound **3f**

m/z	z	Abund	Formula	Ion
64.01661		2956.12		
79.02185	1	89449.36		
80.0251	1	1998.62		
81.0176	1	3883.96		
101.00331		6525.83		
102.12789	1	37337.17		
102.15403		2580.34		
103.1308	1	2492.63		
157.03474		21572.21		
159.03214		2312.44		
215.08068		2003.41		
392.11735	1	180365.86	C20 H17 N5 O2 S	(M+H)+
392.28975		9529.42		
393.11959	1	44797.21	C20 H17 N5 O2 S	(M+H)+
393.16728		3464.83		
394.11712	1	10825.91	C20 H17 N5 O2 S	(M+H)+
395.11733		2256.51		
414.09877	1	65858.85		
414.1567		4620.63		
414.27549		1869.76		
415.10185	1	15590.34		
416.09811	1	3658.51		
430.07302	1	10372.39		
431.07624	1	2702.24		
465.20597		2729.14		
493.23862		3322.5		
805.20844	1	155861.34		
806.21049	1	73406.2		
806.29378	1	5617.53		
806.45746		2283.98		
807.20995	1	27960.51		
807.27548	1	3524.28		
808.20931	1	9022.66		
809.20404	1	1828.18		
836.14314		2054.79		
837.14451	1	9089.01		
838.14664	1	4694.54		
839.1461	1	3049.1		
1250.23776	1	3790.41		
1251.24115	1	3051.2		
1252.23848	1	2310.47		

Table S7. Conversion of mass-based concentrations (µg/mL) to molar units (µM and mM) for compounds **3a-3f**

Comp	Unit	0.5 µg/mL	2.5 µg/mL	5.0 µg/mL	7.5 µg/mL	10.0 µg/mL
**3a**	**µM**	1.44	7.18	14.35	21.53	28.71
	mM	0.00144	0.00718	0.01435	0.02153	0.02871
**3b**	**µM**	1.29	6.44	12.87	19.31	25.75
	mM	0.00129	0.00644	0.01287	0.01931	0.02575
**3c**	**µM**	1.14	5.72	11.43	17.15	22.86
	mM	0.00114	0.00572	0.01143	0.01715	0.02286
**3d**	**µM**	1.33	6.64	13.28	19.92	26.56
	mM	0.00133	0.00664	0.01328	0.01992	0.02656
**3e**	**µM**	1.33	6.64	13.28	19.92	26.56
	mM	0.00133	0.00664	0.01328	0.01992	0.02656
**3f**	**µM**	1.27	6.36	12.73	19.09	25.46
	mM	0.00127	0.00636	0.01273	0.01909	0.02546

Table S8. *In vitro* DPPH free radical scavenging activity of pyrazole-based carbohydrazones (**3a-3f**)

Compound	Inhibition (%)				
	**0.50 µg/mL**	**2.50 µg/mL**	**5.00 µg/mL**	**7.50 µg/mL**	**10.00 µg/mL**
**3a**	62.214±0.191^{f}	65.776±0.220^{e}	69.275±0.191^{e}	71.310±0.110^{d}	74.046±0.001^{d}
**3b**	63.422±0.292^{e}	65.013±0.292^{e}	66.985±0.191^{f}	68.702±0.001^{f}	72.074±0.110^{e}
**3c**	68.066±0.220^{d}	69.975±0.220^{c}	70.674±0.110^{c}	71.883±0.110^{d}	70.611±0.331^{g}
**3d**	63.613±0.110^{e}	67.557±0.001^{d}	68.066±0.110^{e}	71.247±0.110^{d}	74.046±0.001^{d}
**3e**	62.786±0.001^{f}	64.822±0.110^{f}	66.985±0.191^{f}	69.084±0.001^{e}	71.438±0.110^{f}
**3f**	70.293±0.220^{b}	73.092±0.001^{b}	75.064±0.110^{b}	76.463±0.110^{b}	78.499±0.480^{a}
BHA	71.438±0.110^{c}	72.710±0.001^{a}	74.300±0.110^{a}	75.382±0.191^{a}	76.908±0.191^{b}
BHT	66.349±0.480^{a}	68.957±0.110^{c}	70.611±0.001^{c}	73.219±0.110^{c}	74.809±0.001^{c}

Values are expressed as mean ± SD of three replicate measurements (n = 3). Different superscript letters (a-g) within the same column indicate statistically significant differences between groups ($p < 0.05$), as determined by one-way ANOVA followed by Tukey's post hoc test.

Table S9. *In vitro* Ferrous ion chelating activity of pyrazole-based carbohydrazones (**3a-3f**)

Compound	Inhibition (%)				
	**0.50 µg/mL**	**2.50 µg/mL**	**5.00 µg/mL**	**7.50 µg/mL**	**10.00 µg/mL**
**3a**	70.091±0.262^{de}	73.973±0.171^{d}	75.913±0.262^{d}	76.941±0.356^{e}	79.737±0.262^{e}
**3b**	66.039±0.099^{f}	68.836±0.453^{e}	70.833±0.099^{e}	74.372±0.989^{f}	79.852±0.431^{e}
**3c**	69.749±0.431^{e}	75.970±0.356^{c}	77.397±0.297^{c}	80.993±0.297^{c}	82.705±0.171^{c}
**3d**	71.632±0.356^{cd}	73.801±0.171^{d}	76.313±0.198^{d}	79.509±0.099^{d}	81.678±0.297^{d}
**3e**	72.317±0.262^{c}	73.288±0.171^{d}	76.598±0.356^{d}	82.477±0.099^{b}	86.244±0.198^{b}
**3f**	61.644±1.633^{g}	66.495±0.772^{f}	67.979±0.171^{f}	69.178±0.171^{g}	75.685±0.746^{f}
EDTA	79.966±0.342^{b}	85.502±0.099^{a}	90.525±0.099^{a}	91.610±0.000^{a}	92.295±0.171^{a}
BHT	86.187±0.099^{a}	82.648±0.262^{b}	79.053±0.431^{b}	76.256±0.431^{e}	72.717±0.099^{g}

Values are expressed as mean ± SD of three replicate measurements (n = 3). Within each column, values sharing at least one common superscript letter are not significantly different, whereas values with no common superscript letters differ significantly ($p < 0.05$), as determined by one-way ANOVA followed by Tukey's HSD post hoc test.

Table S10. Total antioxidant capacity of pyrazole-based carbohydrazones (**3a-3f**) determined by the ferric thiocyanate method at the 36 h.

Compound	Inhibition (%)				
36th hour	0.50 μg/mL	2.50 μg/mL	5.00 μg/mL	7.50 μg/mL	10.00 μg/mL
**3a**	33.253±0.093^{f}	36.763±0.202^{e}	39.764±0.123^{c}	39.764±0.123^{c}	41.506±0.123^{c}
**3b**	45.713±0.232^{b}	39.657±0.046^{c}	37.165±0.123^{d}	33.521±0.241^{d}	29.662±0.080^{f}
**3c**	48.473±0.161^{a}	38.505±0.161^{d}	33.574±0.123^{f}	28.108±0.202^{f}	22.106±0.161^{h}
**3d**	34.084±0.000^{e}	36.522±0.123^{e}	40.782±0.167^{b}	44.346±0.093^{b}	48.794±0.139^{b}
**3e**	34.325±0.000^{e}	35.745±0.123^{f}	37.138±0.139^{d}	39.603±0.093^{c}	41.024±0.123^{d}
**3f**	37.062±0.171^{d}	43.556±0.000^{a}	47.827±0.186^{a}	52.173±0.154^{a}	55.877±0.086^{a}
Ascorbic acid	26.849±0.080^{g}	28.296±0.080^{g}	29.555±0.093^{g}	31.538±0.167^{e}	32.771±0.123^{e}
α-Tocopherol	44.802±0.123^{c}	42.149±0.093^{b}	36.683±0.046^{e}	33.923±0.000^{d}	26.581±0.123^{g}

Values are expressed as mean ± SD of three replicate measurements (n = 3). Within each column, values sharing at least one common superscript letter are not significantly different, whereas values with no common superscript letters differ significantly (p < 0.05), as determined by one-way ANOVA followed by Tukey's HSD post hoc test.

Table S11. Total antioxidant capacity of pyrazole-based carbohydrazones (**3a-3f**) determined by the ferric thiocyanate (FTC) method at 36 h. Values are expressed as mean ± SD of three replicate measurements (n = 3). Different superscript letters within the same column indicate statistically significant differences between groups (p < 0.05), as determined by one-way ANOVA followed by Tukey's HSD post hoc test.

	Absorbances at 10 μg/mL concentration								
Hour	3a	3b	3c	3d	3e	3f	AA	α-Toc	Control
**12**	0.386	0.447	0.437	0.384	0.447	0.329	0.238	0.408	0.539
**24**	0.464	0.748	0.575	0.419	0.513	0.356	0.490	0.687	0.911
**36**	0.728	0.875	0.969	0.637	0.734	0.596	0.836	0.913	1.244
**48**	0.691	0.825	0.885	0.614	0.563	0.557	0.661	0.868	1.171
**60**	0.489	0.612	0.690	0.571	0.373	0.521	0.506	0.766	0.938

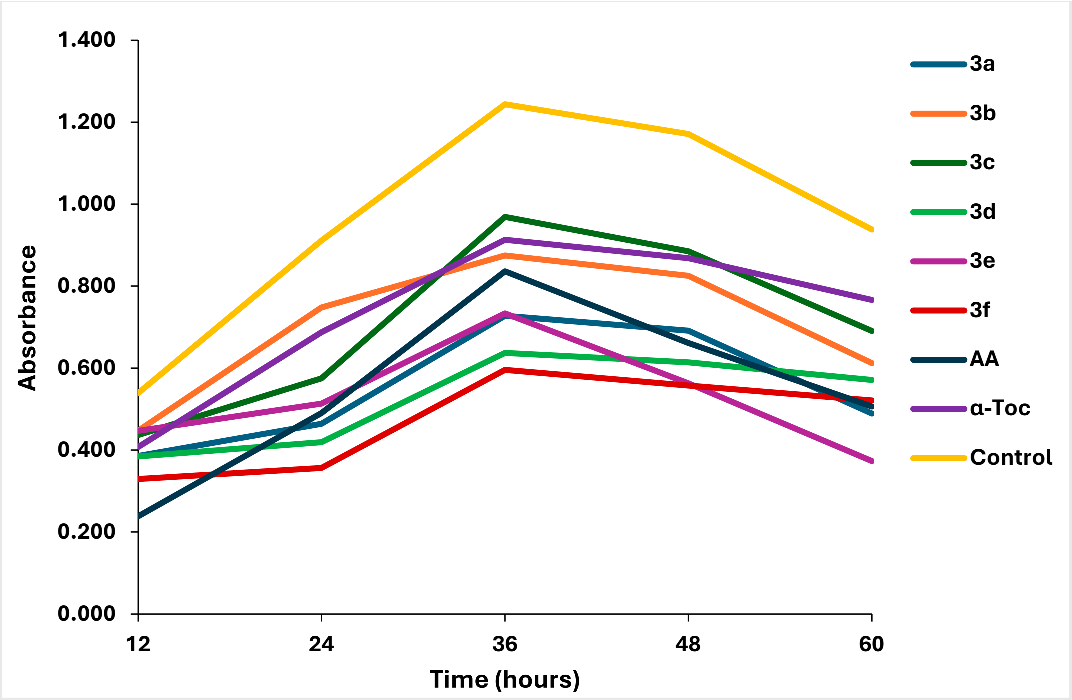

Figure S25. Time-dependent absorbance profiles of compounds **3a-3f** at 10 µg/mL in the FTC assay over a 60 h incubation period, measured at 500 nm. The control showed the highest absorbance values, while all tested compounds exhibited lower absorbance than the control, indicating inhibition of primary lipid peroxidation.

Table S12. *In vitro* Sun protection factor (SPF) values of pyrazole-based carbohydrazones (**3a-3f**) at different concentrations.

Compound	Sun Protection Factor				
	0.50 µg/mL	2.50 µg/mL	5.00 µg/mL	7.50 µg/mL	10.00 µg/mL
**3a**	2.183±0.150^{b}	2.640±0.021^{d}	2.931±0.018^{d}	3.720±0.144^{e}	4.193±0.171^{e}
**3b**	2.566±0.079^{a}	3.067±0.124^{b}	3.352±0.116^{c}	3.971±0.023^{d}	3.992±0.089^{d}
**3c**	2.174±0.081^{b}	2.557±0.087^{d}	3.572±0.118^{b}	3.931±0.064^{c}	4.575±0.087^{c}
**3d**	2.465±0.060^{ab}	4.597±0.042^{a}	4.597±0.025^{ab}	4.767±0.121^{b}	5.169±0.099^{b}
**3e**	2.410±0.146^{a}	2.762±0.078^{c}	3.334±0.074^{c}	3.811±0.132^{c}	4.743±0.141^{c}
**3f**	2.404±0.177^{a}	3.382±0.167^{b}	4.648±0.181^{a}	5.370±0.118^{a}	5.975±0.064^{a}
Carrot seed oil	1.981±0.065^{c}	2.146±0.049^{e}	2.255±0.040^{e}	2.595±0.121^{f}	2.895±0.040^{f}

Values are expressed as mean ± SD of three replicate measurements (n = 3). Within each column, values sharing at least one common superscript letter are not significantly different, whereas values with no common superscript letters differ significantly ($p < 0.05$), as determined by one-way ANOVA followed by Tukey's HSD post hoc test.

Table S13. Pearson correlation coefficients among SPF, DPPH radical scavenging, Fe^{2+} chelation, and FTC antioxidant activity values at 10.00 µg/mL for compounds **3a-3f** (n = 6).

Variable Pairs	Pearson's r	p (two-tailed)
DPPH vs. FTC	0.854	0.030
DPPH vs. Fe^{2+} chelation	-0.821	0.045
FTC vs. Fe^{2+} chelation	-0.431	0.393
SPF vs. DPPH	0.751	0.086
SPF vs. FTC	0.751	0.086
SPF vs. Fe^{2+} chelation	-0.394	0.440

Correlations were calculated using mean values obtained at 10.00 µg/mL for the six synthesized compounds **3a-3f** only; reference standards were excluded because they represent chemically distinct comparators across the different assays. Due to the small dataset (n = 6), these correlations should be interpreted as preliminary indicators of structure-activity trends rather than statistically robust generalizations. All *p* values are two-tailed.

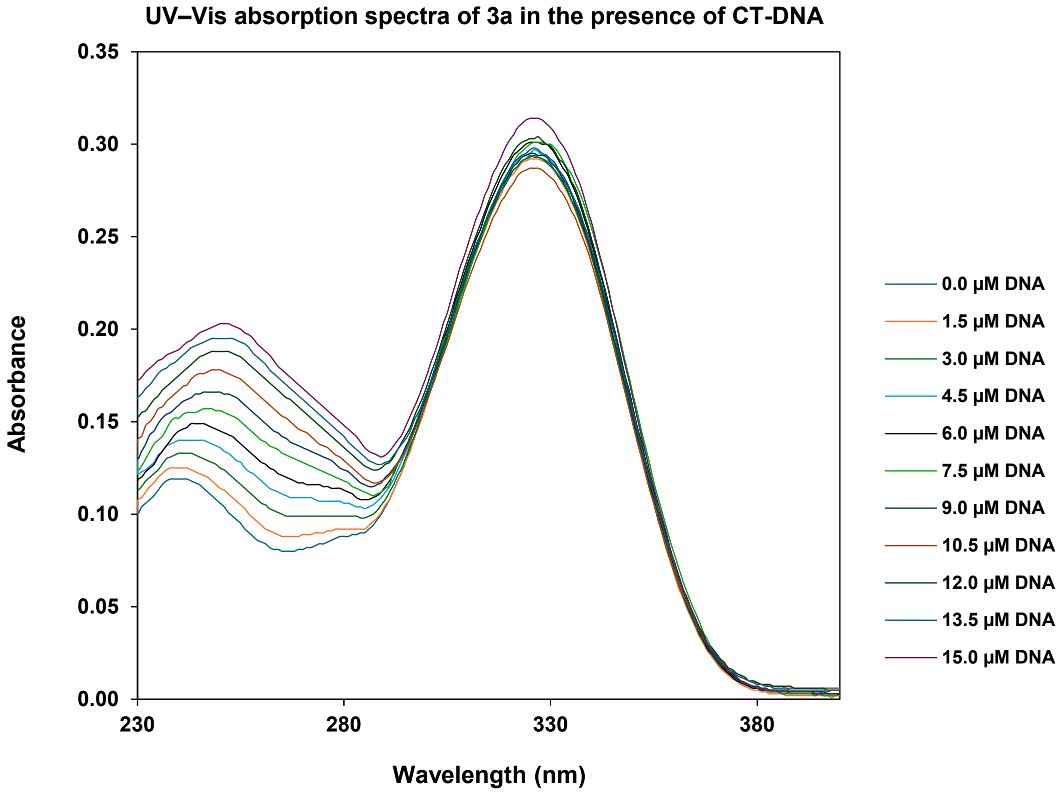

Figure S26. UV-Vis absorption spectra of compound **3a** recorded upon incremental addition of CT-DNA (0.0-15.0 µM) in Tris-HCl buffer (10 mM, pH 7.4) containing 50 mM NaCl at 37 °C. The concentration of **3a** was fixed at 30 µM. A concentration-dependent increase in absorbance was observed in the low-wavelength region; however, this region partially overlaps with intrinsic CT-DNA absorbance and was therefore interpreted qualitatively.

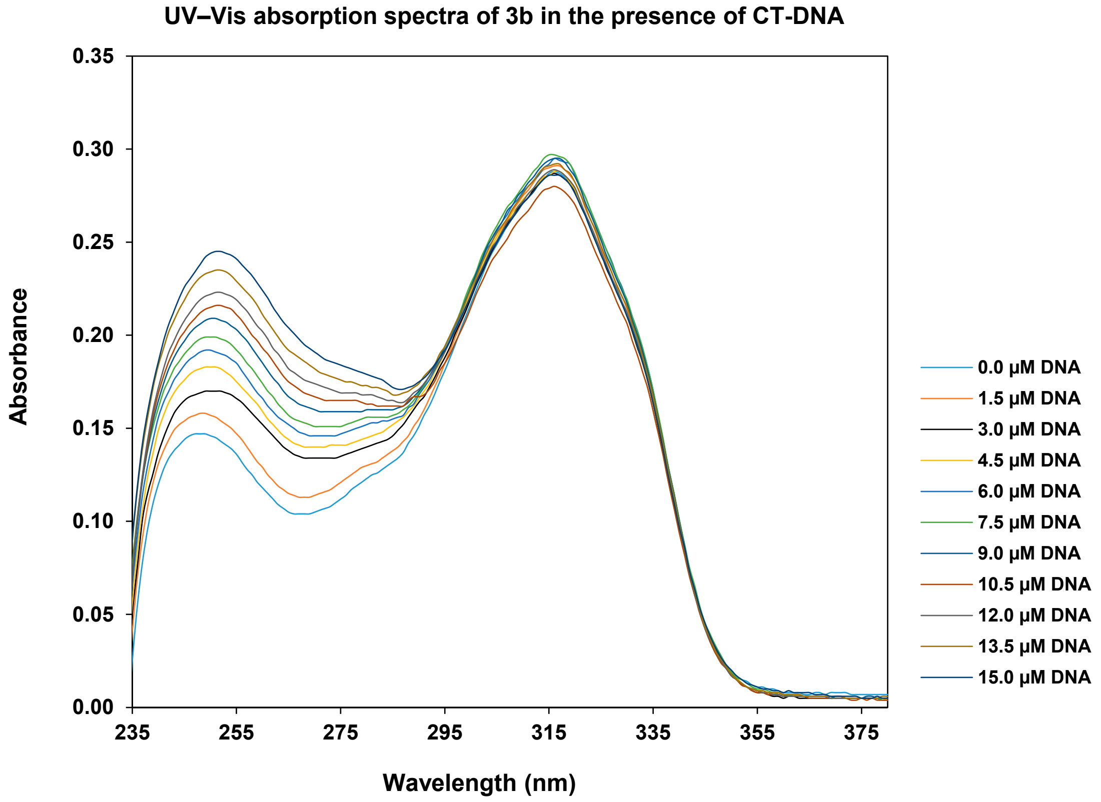

Figure S27. UV-Vis absorption spectra of compound **3b** recorded upon incremental addition of CT-DNA (0.0-15.0 µM) in Tris-HCl buffer (10 mM, pH 7.4) containing 50 mM NaCl at 37 °C. The concentration of **3b** was fixed at 35 µM. A concentration-dependent increase in absorbance was observed following CT-DNA addition. Because the monitored spectral region partially overlaps with CT-DNA absorbance, the observed changes were evaluated qualitatively.

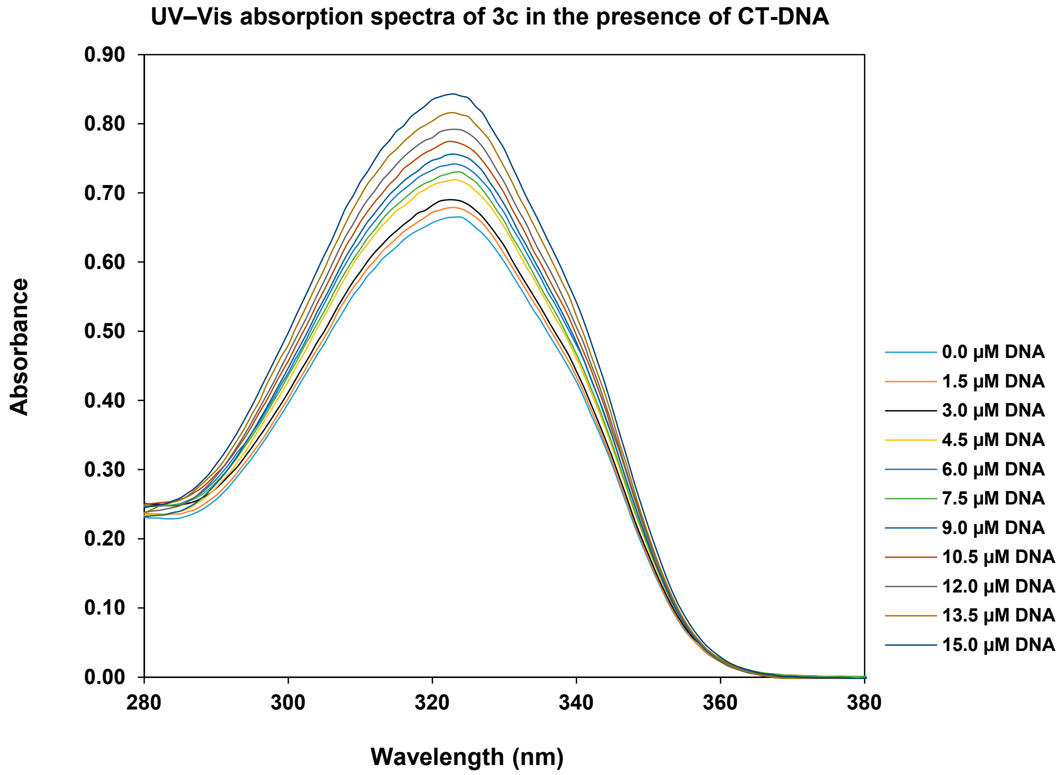

Figure S28. UV-Vis absorption spectra of compound **3c** recorded upon incremental addition of CT-DNA (0.0-15.0 μM) in Tris-HCl buffer (10 mM, pH 7.4) containing 50 mM NaCl at 37 °C. The concentration of **3c** was fixed at 45 μM. A modest concentration-dependent increase in absorbance was observed upon CT-DNA addition.

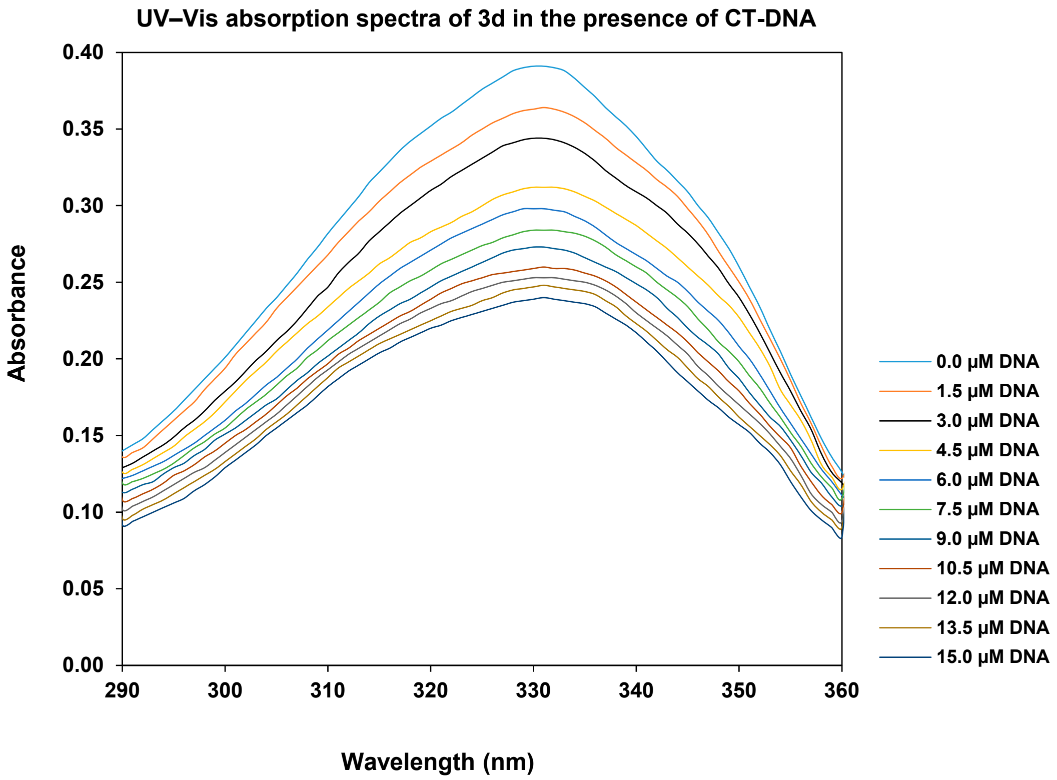

Figure S29. UV-Vis absorption spectra of compound **3d** recorded upon incremental addition of CT-DNA (0.0-15.0 μM) in Tris-HCl buffer (10 mM, pH 7.4) containing 50 mM NaCl at 37 °C. The concentration of **3d** was fixed at 25 μM. A concentration-dependent decrease in absorbance accompanied by a bathochromic shift was observed upon increasing CT-DNA concentration.

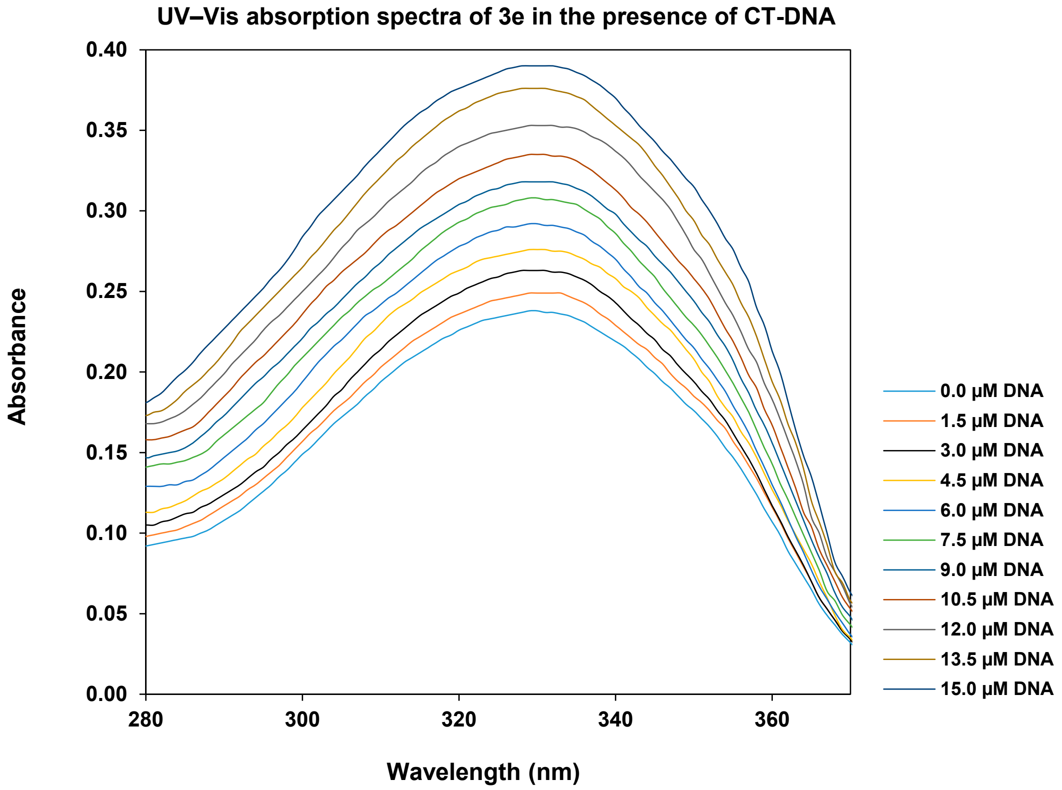

Figure S30. UV-Vis absorption spectra of compound **3e** recorded upon incremental addition of CT-DNA (0.0-15.0 µM) in Tris-HCl buffer (10 mM, pH 7.4) containing 50 mM NaCl at 37 °C. The concentration of **3e** was fixed at 40 µM. CT-DNA addition produced a mild increase in absorbance accompanied by minimal displacement of the absorption maximum.

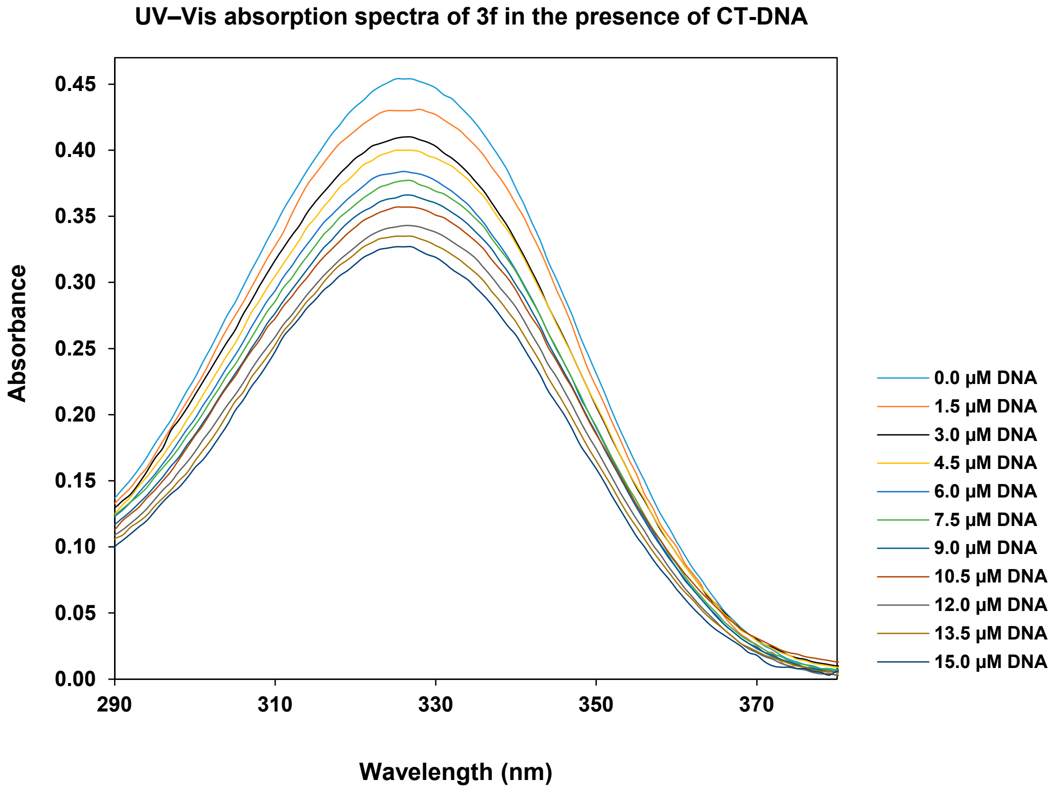

Figure S31. UV-Vis absorption spectra of compound **3f** recorded upon incremental addition of CT-DNA (0.0-15.0 µM) in Tris-HCl buffer (10 mM, pH 7.4) containing 50 mM NaCl at 37 °C. The concentration of **3f** was fixed at 25 µM. A concentration-dependent decrease in absorbance accompanied by a bathochromic shift was observed upon increasing CT-DNA concentration.

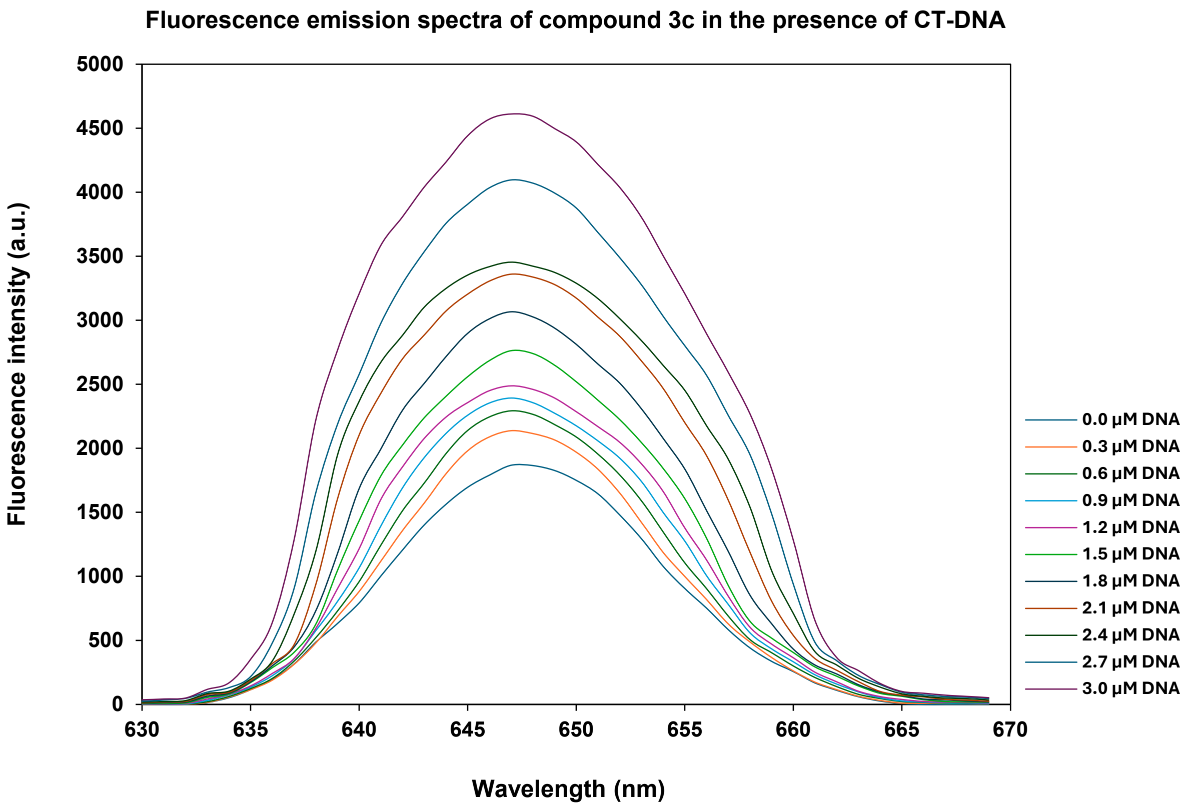

Figure S32. Fluorescence emission spectra of compound **3c** recorded upon incremental addition of CT-DNA (0.0-3.0 μM) in Tris-HCl buffer (10 mM, pH 7.4) containing 50 mM NaCl at 37 °C. The concentration of **3c** was fixed at 45 μM, and fluorescence emission was monitored around $\lambda_{em} \approx 647$ nm following excitation at $\lambda_{ex} = 323$ nm. The spectra show moderate fluorescence enhancement upon increasing CT-DNA concentration.

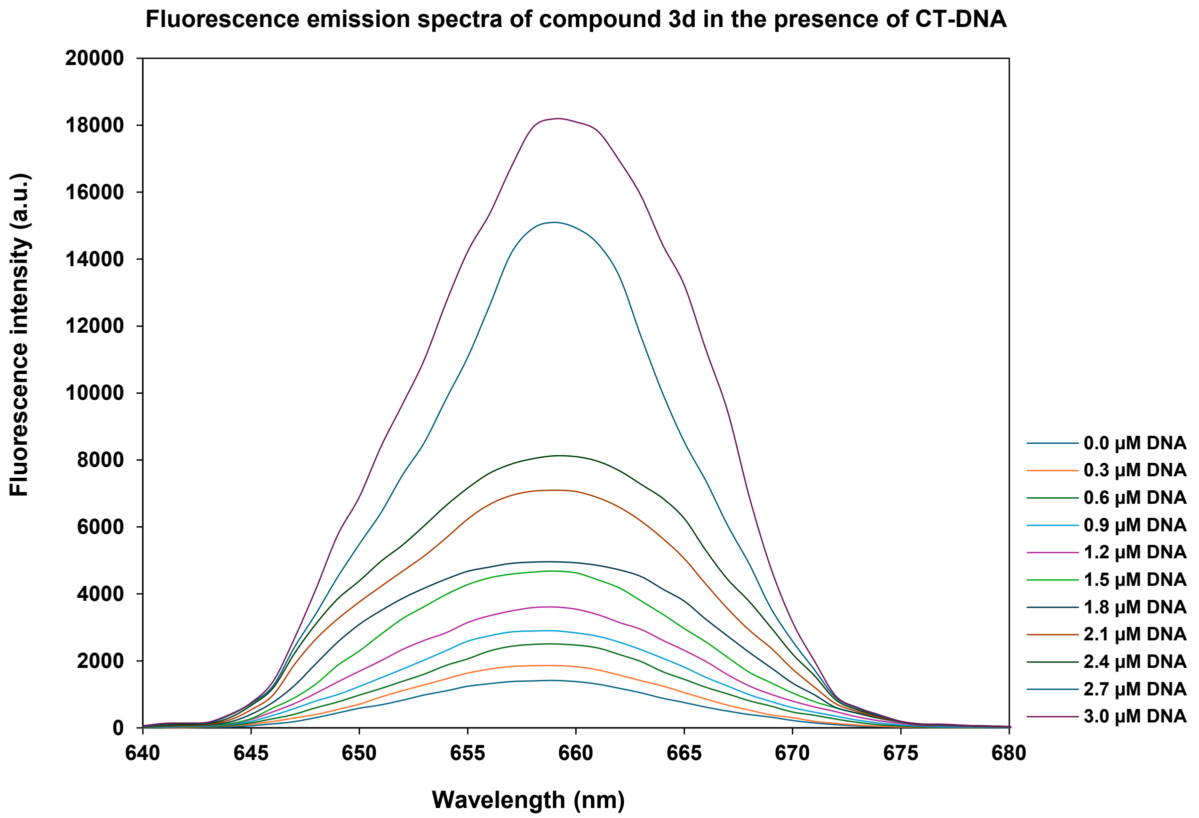

Figure S33. Fluorescence emission spectra of compound **3d** recorded upon incremental addition of CT-DNA (0.0-3.0 µM) in Tris-HCl buffer (10 mM, pH 7.4) containing 50 mM NaCl at 37 °C. The concentration of **3d** was fixed at 25 µM, and fluorescence emission was monitored around λ_{em} ≈ 659 nm following excitation at λ_{ex} = 330 nm. The spectra show pronounced fluorescence enhancement upon increasing CT-DNA concentration.

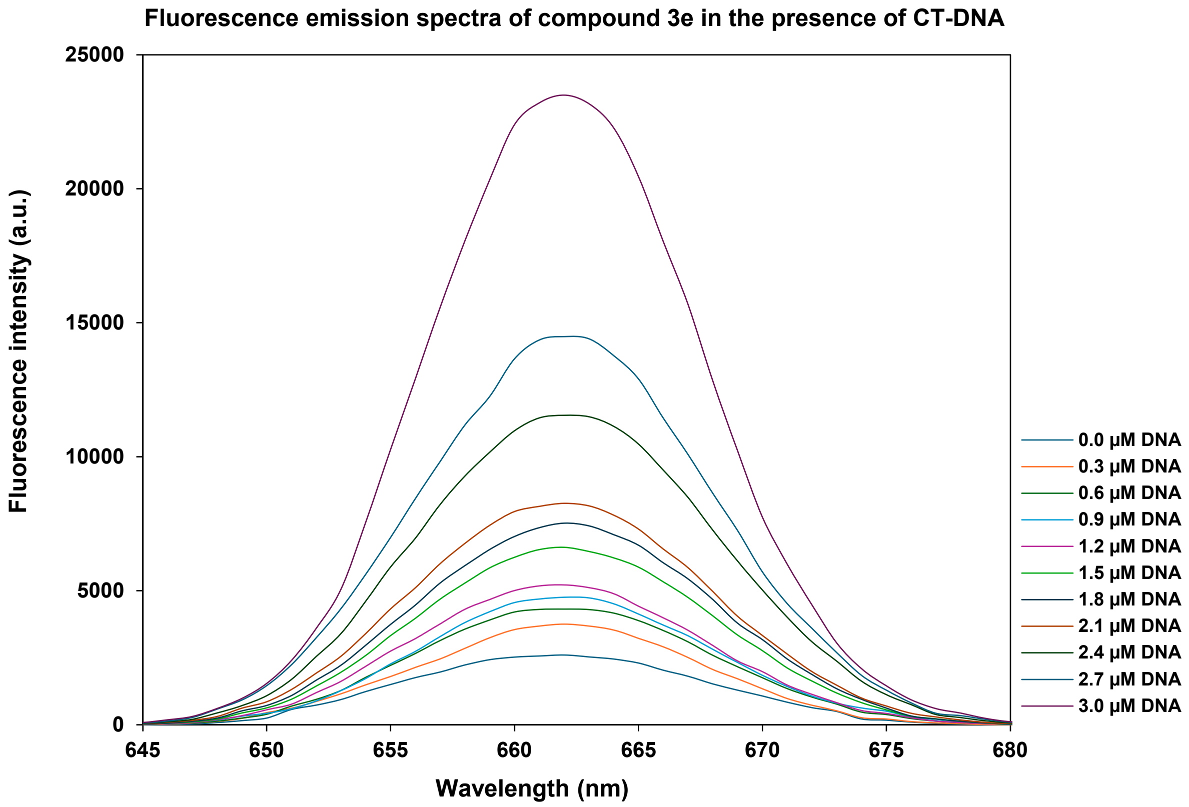

Figure S34. Fluorescence emission spectra of compound **3e** recorded upon incremental addition of CT-DNA (0.0-3.0 μM) in Tris-HCl buffer (10 mM, pH 7.4) containing 50 mM NaCl at 37 °C. The concentration of **3e** was fixed at 40 μM, and fluorescence emission was monitored around $\lambda_{em} \approx 662$ nm following excitation at λ_{ex} = 330 nm. The spectra show strong fluorescence enhancement upon increasing CT-DNA concentration.

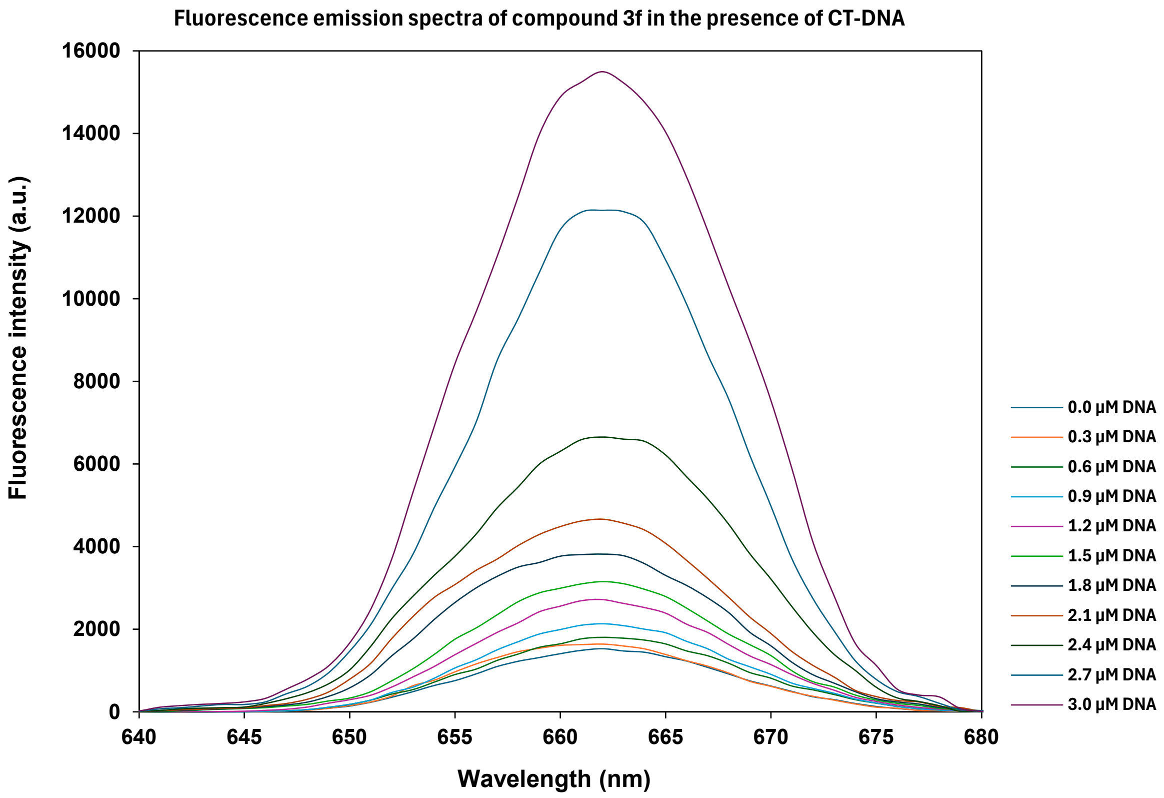

Figure S35. Fluorescence emission spectra of compound **3f** recorded upon incremental addition of CT-DNA (0.0-3.0 μM) in Tris-HCl buffer (10 mM, pH 7.4) containing 50 mM NaCl at 37 °C. The concentration of **3f** was fixed at 25 μM, and fluorescence emission was monitored around $\lambda_{em} \approx 662$ nm following excitation at $\lambda_{ex} = 330$ nm. The spectra show pronounced fluorescence enhancement upon increasing CT-DNA concentration.

Cell Viability Plots of compounds (3a-3f)

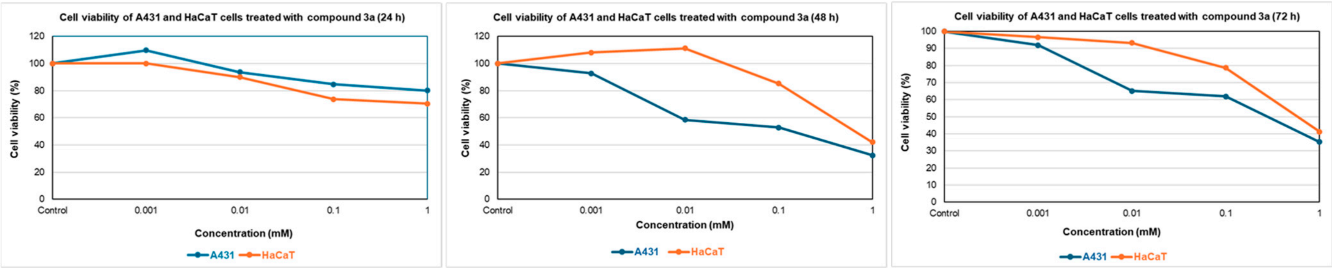

Figure S36. Effect of compound **3a** on cell viability in A431 and HaCaT cells after 24, 48, and 72 h exposure, determined using the MTT assay. Values are expressed as mean ± SD (n = 4)

Compound **3a** caused concentration- and time-dependent reductions in viability in both A431 and HaCaT cells, with greater cytotoxicity seen in A431 cells. Three-way ANOVA showed significant effects of concentration ($p < 0.0001$) and exposure time ($p = 0.0113$), together with a significant cell line × time interaction ($p = 0.0143$). Cytotoxicity was most pronounced at 1 mM during 48-72 h exposure. IC_{50} and selectivity index (*SI*) analyses indicated moderate cancer-cell selectivity, with the most favorable therapeutic window at 48 h (A431 $IC_{50} \approx 0.193$ mM; HaCaT $IC_{50} \approx 0.661$ mM; $SI \approx 3.42$; Table 4). Low-dose hormetic-like responses were occasionally seen, especially in A431 cells at later time points. Overall, **3a** showed moderate growth-inhibitory activity and moderate selectivity (Figure S36). Because MTT reduction reflects metabolic activity rather than direct cell number, viability changes should be interpreted as relative alterations in cellular metabolic viability.

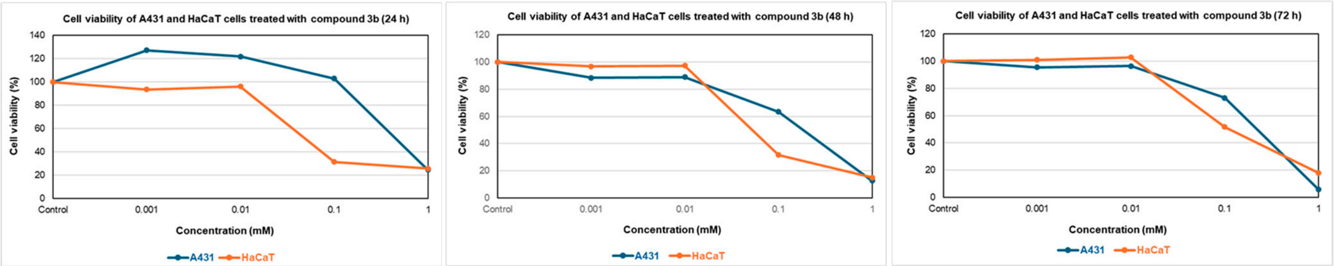

Figure S37. Effect of compound **3b** on cell viability in A431 and HaCaT cells after 24, 48, and 72 h exposure, determined using the MTT assay. Values are expressed as mean ± SD (n = 4)

Compound **3b** demonstrated strong concentration-dependent cytotoxicity in both cell lines, with significantly greater suppression of A431 viability relative to HaCaT cells. Three-way ANOVA revealed significant effects of concentration ($p < 0.0001$), exposure time ($p < 0.0001$), and cell line ($p = 0.0021$), together with a significant concentration × cell line interaction ($p = 0.0048$). At 1 mM, compound 3b produced significant viability reductions across all exposure periods in A431 cells ($p < 0.0001$). HaCaT cells also exhibited substantial viability loss at high concentration, although suppression remained less pronounced than in A431 cells during extended exposure. IC_{50} and *SI* analyses demonstrated improved selectivity with prolonged treatment, with the most favorable therapeutic profile observed at 72 h. Among

the tested compounds, **3b** exhibited one of the strongest combinations of cytotoxic potency and cancer-cell selectivity (Figure S37).

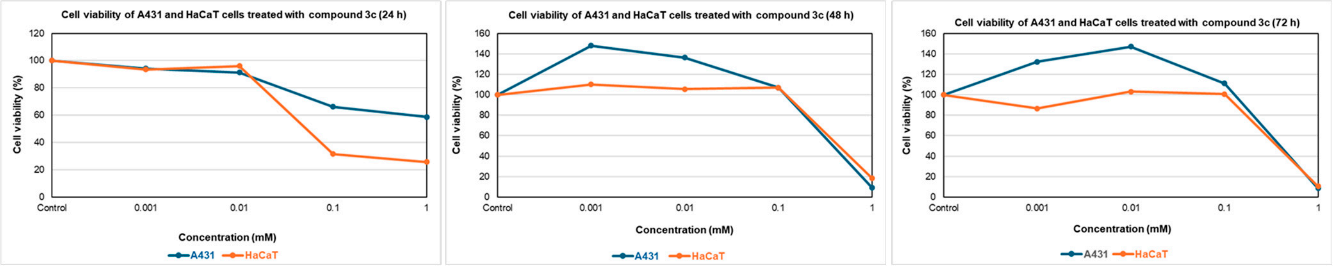

Figure S38. Effect of compound **3c** on cell viability in A431 and HaCaT cells after 24, 48, and 72 h exposure, determined using the MTT assay. Values are expressed as mean ± SD (n = 4)

Compound **3c** displayed strong growth-inhibitory activity, with pronounced time- and concentration-dependent viability reductions, especially in A431 cells. Significant effects of concentration ($p < 0.0001$), exposure time ($p < 0.0001$), and cell line ($p = 0.0017$) were seen, together with a significant concentration × cell line interaction ($p = 0.0032$). The strongest effects occurred at 1 mM during 48-72 h exposure, where A431 viability approached near-background absorbance levels. HaCaT cells also showed significant viability loss at high concentration, although suppression remained greater in A431 cells. IC_{50} and *SI* analyses indicated moderate-to-high cancer-cell selectivity, with the most favorable therapeutic window at 72 h. Overall, **3c** combined strong cytotoxic potency with sustained preferential activity (Figure S38).

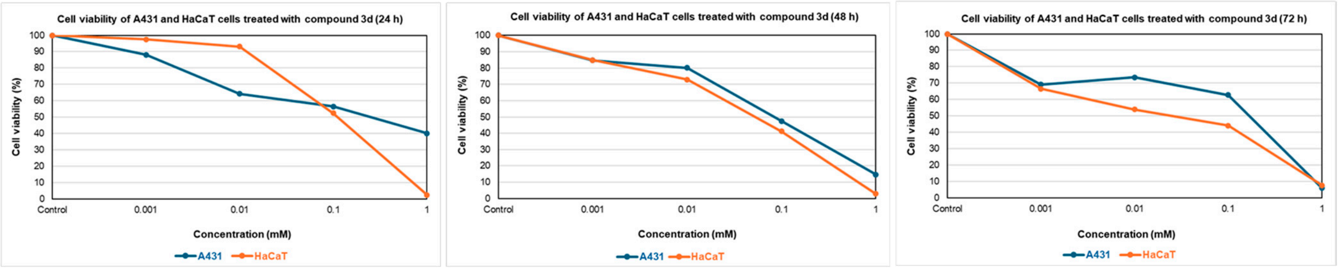

Figure S39. Effect of compound **3d** on cell viability in A431 and HaCaT cells after 24, 48, and 72 h exposure, determined using the MTT assay. Values are expressed as mean ± SD (n = 4).

Compound **3d** exhibited marked growth-inhibitory activity, with clear concentration- and time-dependent viability reductions in both A431 and HaCaT cells. Statistical analysis showed a highly significant concentration effect ($p < 0.0001$), together with significant effects of exposure time and cell line, reflecting progressive cytotoxicity and differential cellular sensitivity. The strongest suppression occurred at 1 mM during 48-72 h exposure, where absorbance values in both cell lines approached near-background levels. Although A431 viability declined at higher concentrations, HaCaT cells showed equal or greater sensitivity, especially after prolonged exposure (Figure S39).

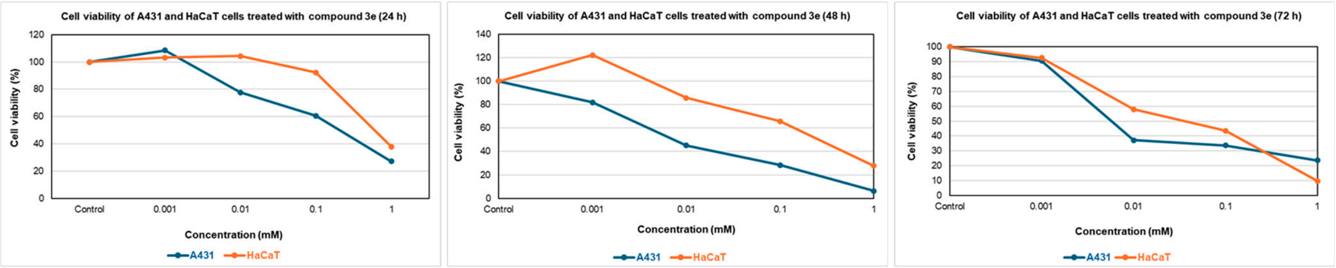

Figure S40. Effect of compound **3e** on cell viability in A431 and HaCaT cells after 24, 48, and 72 h exposure, determined using the MTT assay. Values are expressed as mean ± SD (n = 4)

Compound **3e** exhibited pronounced concentration- and time-dependent cytotoxicity with variable selectivity across exposure periods. Three-way ANOVA showed significant effects of concentration ($p < 0.0001$), exposure time ($p < 0.0001$), and cell line ($p = 0.0063$), as well as a significant concentration × cell line interaction ($p = 0.0027$). The strongest cytotoxic effects were seen at 1 mM, especially during 48 h exposure, when A431 viability approached near-background levels in several replicates. IC_{50} and *SI* analyses showed the most favorable selectivity at 48 h, with strong preferential suppression of A431 cells relative to HaCaT cells. However, this selectivity was not maintained at later exposure periods, suggesting reduced selectivity during prolonged exposure and possible delayed HaCaT sensitivity. Overall, **3e** combined strong growth-inhibitory activity with context-dependent selectivity (Figure S40).

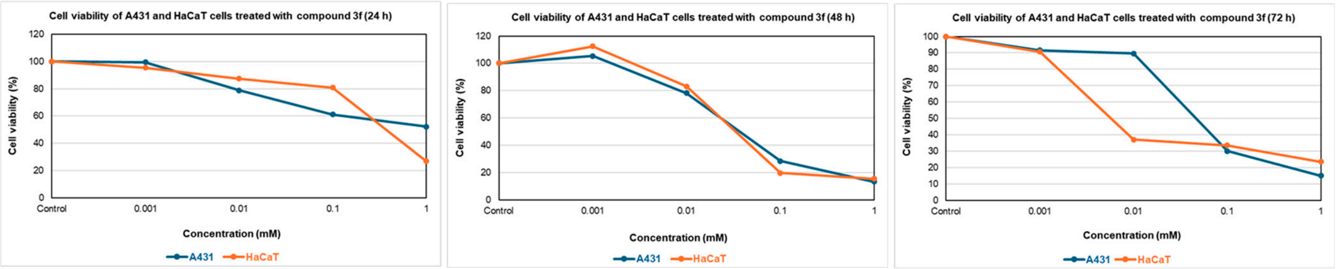

Figure S41. Effect of compound **3f** on cell viability in A431 and HaCaT cells after 24, 48, and 72 h exposure, determined using the MTT assay. Values are expressed as mean ± SD (n = 4)

Compound **3f** showed strong concentration-dependent cytotoxicity in both A431 and HaCaT cells, although its selectivity was weaker than that of compounds **3b** and **3c**. Three-way ANOVA identified significant effects of concentration ($p < 0.0001$), exposure time ($p < 0.0001$), and cell line ($p = 0.0091$), together with a significant concentration × time interaction ($p = 0.0018$). High-dose treatment markedly reduced viability in both cell lines, with substantial concurrent HaCaT toxicity limiting the therapeutic window. IC_{50} and *SI* analyses indicated moderate selectivity at 48 h, although preferential suppression of A431 cells was less pronounced than for **3b** and **3c**. These findings suggest that **3f** retains strong cytotoxic potency but shows broader nonspecific toxicity toward non-malignant keratinocytes (Figure S41).

Table S14. Three-way ANOVA summary for the effects of cell line, exposure time, and concentration on normalized MTT cell viability in A431 and HaCaT cells treated with compounds **3a-3f.** Values are reported as F-statistics with corresponding degrees of freedom and p-values. Significant effects were defined as $p < 0.05$. Concentration was significant for all compounds, indicating dose-dependent cytotoxicity across the series. Data were analyzed from the normalized viability dataset.

Compd.	Effect	df	F	*p*-value	Sig
**3a**	Cell line	1.90	2.27	0.136	ns
	Time	2.90	4.71	0.0113	*
	Concentration	4.90	19.60	<0.001	***
	Cell line × Time	2.90	4.45	0.0143	*
	Cell line × Concentration	4.90	0.80	0.531	ns
	Time × Concentration	8.90	0.58	0.790	ns
	Cell line × Time × Concentration	8.90	0.65	0.735	ns
**3b**	Cell line	1.90	2.82	0.0966	ns
	Time	2.90	1.19	0.310	ns
	Concentration	4.90	52.56	<0.001	***
	Cell line × Time	2.90	6.05	0.00343	**
	Cell line × Concentration	4.90	1.39	0.243	ns
	Time × Concentration	8.90	0.45	0.891	ns
	Cell line × Time × Concentration	8.90	1.86	0.0761	ns
**3c**	Cell line	1.90	11.74	<0.001	***
	Time	2.90	3.14	0.0481	*
	Concentration	4.90	53.24	<0.001	***
	Cell line × Time	2.90	0.81	0.449	ns
	Cell line × Concentration	4.90	0.90	0.467	ns
	Time × Concentration	8.90	4.82	<0.001	***
	Cell line × Time × Concentration	8.90	1.34	0.233	ns
**3d**	Cell line	1.90	2.04	0.156	ns
	Time	2.90	1.70	0.188	ns
	Concentration	4.90	44.54	<0.001	***
	Cell line × Time	2.90	0.31	0.732	ns
	Cell line × Concentration	4.90	0.55	0.701	ns
	Time × Concentration	8.90	0.60	0.772	ns
	Cell line × Time × Concentration	8.90	1.00	0.443	ns
**3e**	Cell line	1.90	24.01	<0.001	***
	Time	2.90	15.95	<0.001	***
	Concentration	4.90	80.47	<0.001	***
	Cell line × Time	2.90	7.20	0.00126	**
	Cell line × Concentration	4.90	3.16	0.0177	*
	Time × Concentration	8.90	2.47	0.0180	*
	Cell line × Time × Concentration	8.90	1.05	0.403	ns
**3f**	Cell line	1.90	1.75	0.189	ns
	Time	2.90	15.20	<0.001	***
	Concentration	4.90	109.41	<0.001	***
	Cell line × Time	2.90	0.81	0.450	ns
	Cell line × Concentration	4.90	1.44	0.229	ns
	Time × Concentration	8.90	5.02	<0.001	***
	Cell line × Time × Concentration	8.90	3.03	0.00463	**

Normalized MTT viability (%) was analyzed separately for each compound using a three-way ANOVA with cell line, exposure time, and concentration as fixed factors. Values shown are degrees of freedom (df), F statistic, p-value, and significance code. Significance codes: ns, not significant; * $p < 0.05$; ** $p < 0.01$; *** $p < 0.001$. Tukey's HSD post hoc comparisons were performed within each cell line and time point, comparing each treatment concentration with its corresponding control.

Table S15. Numerical MTT cell viability data corresponding to Figures 9-11 and Figures S36-S41. A431 epidermoid carcinoma cells and HaCaT immortalized human keratinocytes were treated with compounds 3a–3f at concentrations ranging from 0.001 to 1 mM for 24, 48, and 72 h. Cell viability is expressed as mean ± SD (n = 4) relative to the corresponding untreated control (100% viability). Standard deviation values are provided to document experimental variability across independent replicates.

		A431				HaCaT			
		Concentration (mM)							
**Comp**	**Time (h)**	**0.001**	**0.01**	**0.1**	**1**	**0.001**	**0.01**	**0.1**	**1**
**3a**	**24**	109.723 ± 0.037	93.652 ± 0.056	84.653 ± 0.053	80.153 ± 0.353	100.152 ± 0.008	90.025 ± 0.009	73.722 ± 0.008	70.430 ± 0.099
	**48**	92.729 ± 0.239	58.468 ± 0.064	52.829 ± 0.090	32.387 ± 0.026	108.105 ± 0.041	111.269 ± 0.047	85.173 ± 0.020	42.043 ± 0.023
	**72**	91.888 ± 0.240	65.115 ± 0.089	61.947 ± 0.065	35.298 ± 0.028	96.551 ± 0.097	93.277 ± 0.065	78.599 ± 0.135	41.148 ± 0.142
**3b**	**24**	127.075 ± 0.063	121.766 ± 0.145	102.944 ± 0.152	24.566 ± 0.018	93.455 ± 0.024	96.090 ± 0.024	31.492 ± 0.034	25.669 ± 0.012
	**48**	88.358 ± 0.152	88.964 ± 0.188	63.354 ± 0.167	12.674 ± 0.030	96.759 ± 0.005	97.310 ± 0.014	31.690 ± 0.117	15.104 ± 0.014
	**72**	95.541 ± 0.081	96.460 ± 0.256	73.130 ± 0.167	5.919 ± 0.018	101.007 ± 0.095	102.786 ± 0.083	51.704 ± 0.065	17.934 ± 0.019
**3c**	**24**	94.284 ± 0.182	91.352 ± 0.147	66.105 ± 0.078	58.630 ± 0.056	93.455 ± 0.024	96.090 ± 0.034	31.492 ± 0.010	25.670 ± 0.012
	**48**	147.981 ± 0.049	136.446 ± 0.292	107.121 ± 0.192	9.361 ± 0.006	110.069 ± 0.020	105.517 ± 0.039	106.944 ± 0.035	18.480 ± 0.006
	**72**	132.274 ± 0.294	147.016 ± 0.094	111.068 ± 0.081	8.541 ± 0.031	86.699 ± 0.087	103.206 ± 0.080	100.797 ± 0.093	10.432 ± 0.014
**3d**	**24**	88.042 ± 0.180	64.310 ± 0.280	56.471 ± 0.087	40.055 ± 0.028	97.554 ± 0.020	93.167 ± 0.135	52.341 ± 0.077	2.446 ± 0.009
	**48**	84.701 ± 0.173	80.199 ± 0.255	47.546 ± 0.164	14.710 ± 0.069	84.873 ± 0.051	72.976 ± 0.049	41.133 ± 0.104	2.881 ± 0.018
	**72**	69.107 ± 0.131	73.474 ± 0.201	62.786 ± 0.1041	5.919 ± 0.018	66.549 ± 0.051	53.877 ± 0.065	43.972 ± 0.1036	7.565 ± 0.018
**3e**	**24**	108.456 ± 0.062	77.620 ± 0.066	60.524 ± 0.037	27.298 ± 0.015	103.192 ± 0.022	104.285 ± 0.011	92.261 ± 0.008	37.954 ± 0.012
	**48**	81.920 ± 0.042	45.255 ± 0.075	28.452 ± 0.020	6.258 ± 0.006	122.324 ± 0.040	85.714 ± 0.029	65.858 ± 0.062	27.855 ± 0.046
	**72**	90.678 ± 0.190	37.132 ± 0.049	33.564 ± 0.053	23.594 ± 0.080	92.646 ± 0.186	58.044 ± 0.091	43.621 ± 0.091	9.623 ± 0.051
**3f**	**24**	99.370 ± 0.176	78.799 ± 0.094	61.181 ± 0.084	52.223 ± 0.042	95.475 ± 0.045	87.445 ± 0.018	80.790 ± 0.005	26.841 ± 0.016
	**48**	105.331 ± 0.085	78.251 ± 0.046	28.520 ± 0.093	13.382 ± 0.011	112.539 ± 0.112	83.202 ± 0.091	19.787 ± 0.0115	15.424 ± 0.015
	**72**	91.549 ± 0.091	89.634 ± 0.229	30.017 ± 0.089	14.969 ± 0.091	90.678 ± 0.190	37.132 ± 0.049	33.564 ± 0.054	23.593 ± 0.080

Wound closure results for all compounds

The effects of compounds **3a-3f** on wound closure were evaluated in A431 and HaCaT cells using a scratch assay. Wound closure was quantified at 14 h and 18 h relative to the initial wound area at 0 h for each replicate. Data are expressed as mean ± SD from three independent biological replicates. Across all compounds, no consistent inhibition of wound closure was seen at the tested concentration (10 μM). These findings suggest that, under these conditions, the compounds do not exhibit migration-inhibitory activity and may influence cellular processes contributing to wound closure.

Compound **3a** did not produce sustained inhibition of wound closure. In A431 cells, closure decreased significantly at 14 h (p = 0.0408), with treated cells reaching 36.9 ± 6.5% compared with 51.7 ± 2.9% in control. However, no significant difference was detected at 18 h (p = 0.1438), when closure in treated cells (68.1 ± 4.6%) exceeded control (61.5 ± 4.3%). In HaCaT cells, closure was higher than control at both time points, although differences were not significant ($p > 0.05$). Thus, **3a** induced a transient reduction in A431 wound closure that was not maintained.

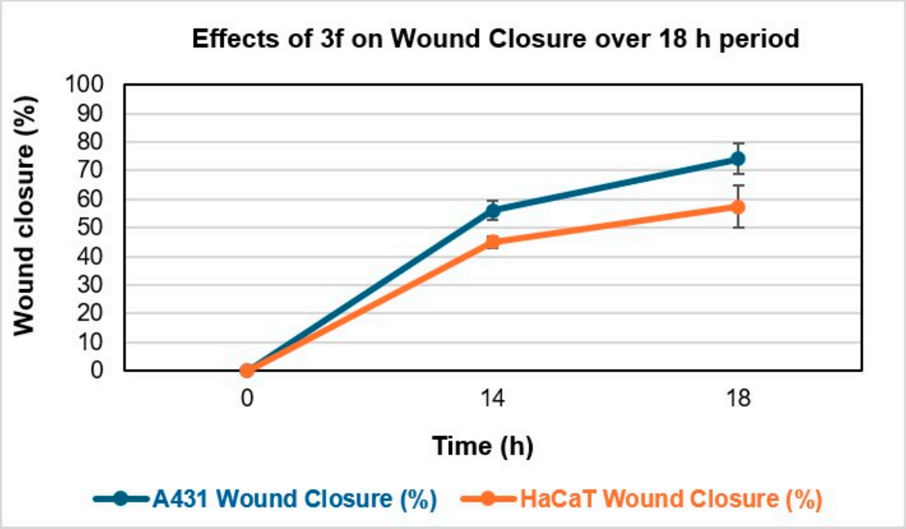

Figure S42. Time-dependent wound closure responses of A431 and HaCaT cells treated with compound **3a**

Compound **3b** did not inhibit wound closure. In A431 cells, no significant difference was seen at 14 h (p = 0.571), whereas closure increased significantly at 18 h (p = 0.0046), reaching 81.2 ± 2.8% compared with 61.5 ± 4.3% in control. In HaCaT cells, closure was higher than control at both time points but not significant (p > 0.05). These results indicate increased late-stage wound closure in A431 cells.

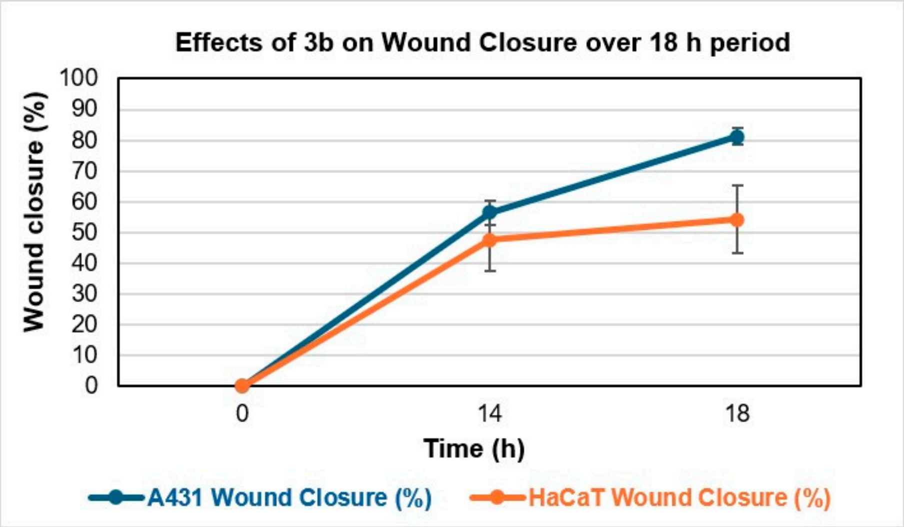

Figure S43. Time-dependent wound closure responses of A431 and HaCaT cells treated with compound **3b**

Compound **3c** increased wound closure in A431 cells at both 14 h (69.4 ± 4.0%, p = 0.0045) and 18 h (81.6 ± 1.9%, p = 0.0070) compared with control. In HaCaT cells, closure values were higher than control at both time points but not significant (p > 0.05). These findings indicate increased A431 wound closure under these conditions.

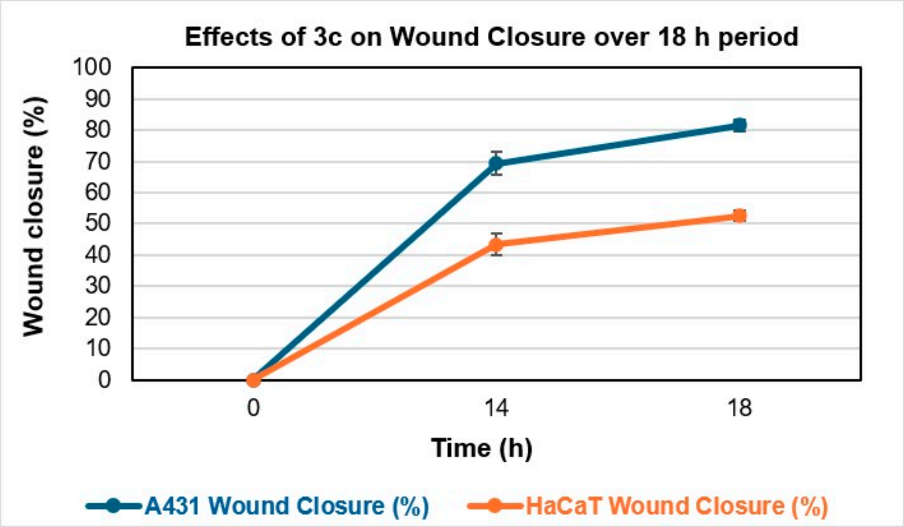

Figure S44. Time-dependent wound closure responses of A431 and HaCaT cells treated with compound **3c**

Compound **3d** increased wound closure in A431 cells at both 14 h (p = 0.012) and 18 h (p = 0.0055), with closure values of 65.5 ± 4.2% and 79.6 ± 3.6%, respectively. In HaCaT cells, closure was higher than control, especially at 18 h, although this effect did not reach significance (p = 0.057). These results suggest enhanced A431 wound closure.

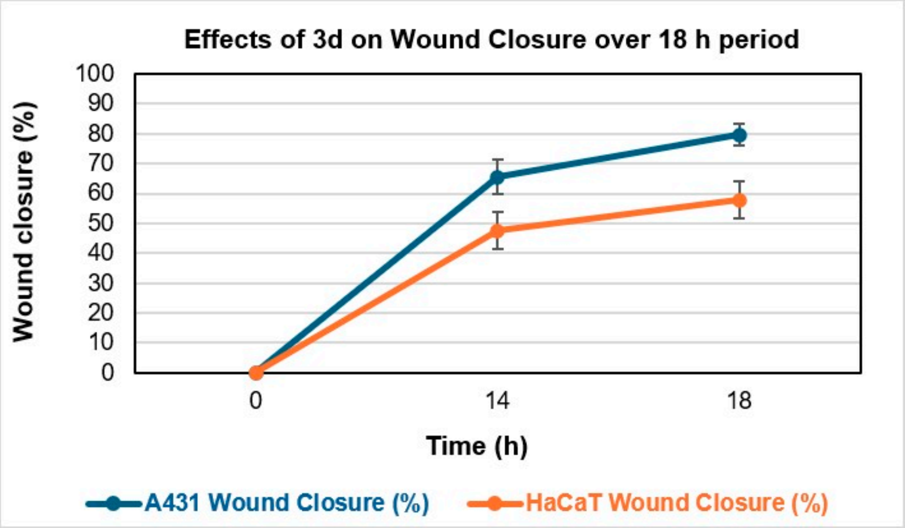

Figure S45. Time-dependent wound closure responses of A431 and HaCaT cells treated with compound **3d**

Compound **3e** increased wound closure in A431 cells at both 14 h (60.8 ± 3.7%, p = 0.033) and 18 h (76.6 ± 4.4%, p = 0.013). In HaCaT cells, closure values were higher than control at both time points, with a trend toward increased wound closure at 18 h (p = 0.053). These findings indicate increased wound closure,especially in A431 cells.

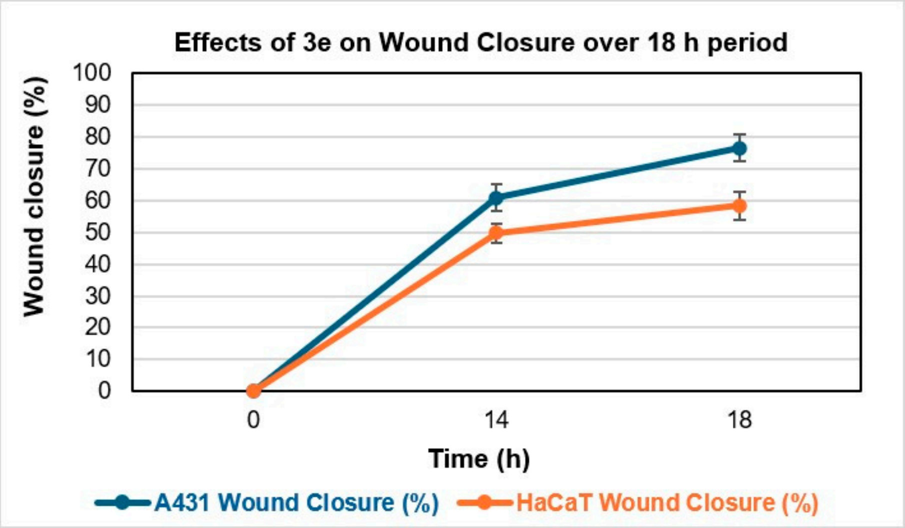

Figure S46. Time-dependent wound closure responses of A431 and HaCaT cells treated with compound **3e**

Compound **3f** did not significantly affect A431 closure at 14 h (p = 0.187), but increased closure at 18 h (p = 0.036), reaching 74.1 ± 5.4% compared with 61.5 ± 4.3% in control. In HaCaT cells, closure values were consistently higher than control but not significant (p > 0.05). These results indicate delayed enhancement of A431 wound closure.

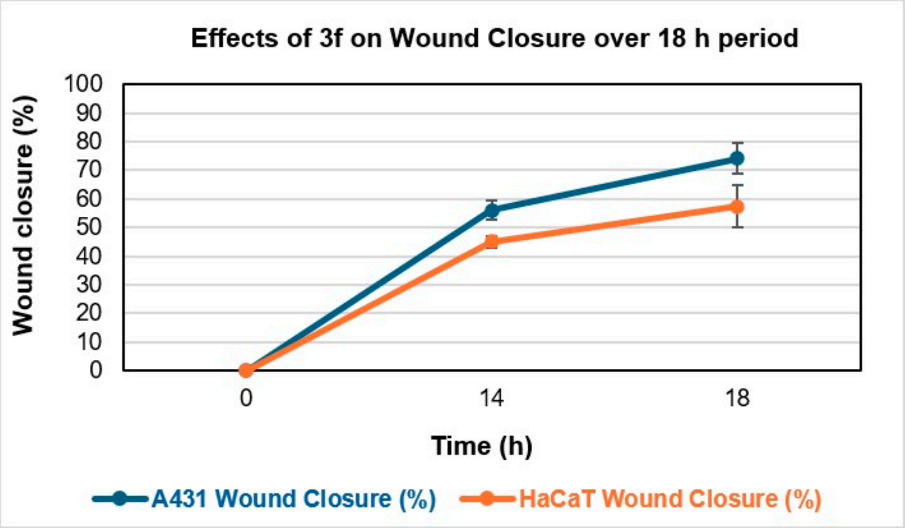

Figure S47. Time-dependent wound closure responses of A431 and HaCaT cells treated with compound **3f**
